# Gene reactivation upon erosion of X chromosome inactivation in female hiPSCs is predictable yet variable and persists through differentiation

**DOI:** 10.1016/j.stemcr.2025.102472

**Published:** 2025-04-03

**Authors:** Ana Cláudia Raposo, Paulo Caldas, Joana Jeremias, Maria Arez, Francisca Cazaux Mateus, Pedro Barbosa, Rui Sousa-Luís, Frederico Água, David Oxley, Annalisa Mupo, Melanie Eckersley-Maslin, Miguel Casanova, Ana Rita Grosso, Simão Teixeira da Rocha

**Affiliations:** 1 iBB - Institute for Bioengineering and Biosciences and Department of Bioengineering, Instituto Superior Técnico, Universidade de Lisboa, Lisbon, Portugal; 2 Associate Laboratory i4HB Institute for Health and Bioeconomy, Instituto Superior Técnico, Universidade de Lisboa, Lisbon, Portugal; 3 Instituto de Medicina Molecular, João Lobo Antunes, Faculdade de Medicina, Universidade de Lisboa, Lisbon, Portugal; 4 Department of Life Sciences, UCIBIO - Applied Molecular Biosciences Unit, NOVA School of Science and Technology, NOVA University Lisbon, Caparica, Portugal; 5 LASIGE, Departamento de Informática, Faculdade de Ciências, Universidade de Lisboa, 1749-016 Lisbon, Portugal; 6 Sir William Dunn School of Pathology, University of Oxford, Oxford, UK; 7 Mass Spectrometry Facility, The Babraham Institute, Cambridge, UK; 8 Epigenetics Programme, Babraham Institute, Cambridge CB22 3AT, UK; 9 Altos Labs, Cambridge, UK; 10 Peter MacCallum Cancer Centre, Melbourne, VIC 3000, Australia; 11 Sir Peter MacCallum Department of Oncology, The University of Melbourne, Melbourne, VIC 3010, Australia; 12 Department of Anatomy and Physiology, The University of Melbourne, Melbourne, VIC 3010, Australia

**Keywords:** epigenetics, X-chromosome inactivation, XCI, DNA methylation, induced pluripotent stem cells, hiPSCs

## Abstract

Female human induced pluripotent stem cells frequently undergo X-chromosome inactivation (XCI) erosion, marked by X-inactive specific transcript (XIST) RNA loss and partial reactivation of the inactive X (Xi). This overlooked phenomenon limits our understanding of its impact on stem cell applications. Here, we show that XCI erosion is frequent and heterogeneous, leading to the reactivation of several X-linked genes. These are primarily located on the short arm of the X chromosome, particularly near escape genes and within H3K27me3-enriched domains, with reactivation linked to reduced promoter DNA methylation. Interestingly, escape genes further increase their expression from Xi upon XCI erosion, highlighting the critical role of XIST in their dosage regulation. Importantly, global (hydroxy)methylation levels and imprinted regions remain unaffected, and analysis of trilineage commitment and cardiomyocyte formation reveals that XCI erosion persists across differentiation. These findings underscore the need for greater awareness of the implications of XCI erosion for stem cell research and clinical applications.

## Highlights

• XCI erosion is frequent and heterogeneous in female hiPSCs
• Some genes, including escape genes, are more prone to XCI erosion
• XCI erosion typically does not alter global DNA methylation or imprinting profiles
• Heterogeneous XCI erosion persists in differentiated hiPSC derivatives

## Teaser

In this article, da Rocha and colleagues show that XCI erosion is frequent yet variable in female human iPSCs, leading to reactivation of some X-linked genes, including escapees. This reactivation is tied to specific epigenetic features but occurs without global DNA methylation changes and persists through differentiation, highlighting the need to consider XCI erosion in stem cell research and applications.

## Introduction

X-chromosome inactivation (XCI) in female mammals ensures dosage compensation between females (XX) and males (XY) through transcriptional silencing of one X chromosome (Loda et al., 2022; Patrat et al., 2020). This process is indispensable for female survival (Penny et al., 1996; Marahrens et al., 1997), being established in post-implantation embryos and faithfully maintained throughout life (Werner et al., 2022). The key regulator of XCI is the X-inactive specific transcript (XIST), a long non-coding RNA (lncRNA) that remains expressed only from the inactive X chromosome (Xi), which is randomly chosen for inactivation (reviewed in Patrat et al., 2020). XIST RNA coats the Xi and recruits several RNA binding proteins (RBPs) and chromatin modifiers, inducing a stable transcriptional silencing across the entire chromosome (da Rocha and Heard, 2017; Raposo et al., 2021). *XIST* remains expressed in all somatic cells, maintaining XCI with cell-specific nuances (Richart et al., 2022; Yu et al., 2021). Importantly, more than 15% of X-linked genes, known as escapees, evade inactivation on the Xi (Carrel and Willard, 2005; Tukiainen et al., 2017; Werner et al., 2022). Escapees could either constitutively evade inactivation (named escape genes or escapees in this article) or escape only in certain cell types or individuals (named variable genes).

Distinct from the *in vivo* situation, in clinically relevant female human pluripotent stem cells (hPSCs), namely embryonic stem cells (hESCs) and induced pluripotent stem cells (hiPSCs) derived and cultured in conventional/primed conditions, *XIST* expression is recurrently lost upon cell passage (Mekhoubad et al., 2012; Vallot et al., 2015). Loss of *XIST* expression leads to the progressive re-activation of the Xi, a process commonly known as XCI erosion (Bansal et al., 2021; Mekhoubad et al., 2012; Nazor et al., 2012; Sahakyan et al., 2018). The eroded X chromosome (Xe) is partially reactivated and is characterized by the loss of H3K27me3 histone modification across the Xi (Mekhoubad et al., 2012; Vallot et al., 2015) and DNA demethylation at the reactivated X-linked gene promoters (Geens et al., 2016; Patel et al., 2017; Bansal et al., 2021; Fukuda et al., 2021). XCI erosion can be easily tracked using genetic variants since hPSCs are clonal, meaning all cells share the same Xi, rather than being a mix of maternal or paternal X-inactivated cells (Tchieu et al., 2010).

Cell passage is the strongest driver for XCI erosion (Silva et al., 2008; Geens et al., 2016; Patel et al., 2017; Fukuda et al., 2021), with the rate of erosion being influenced by the culture conditions (Cloutier et al., 2022). Preventing or correcting XCI erosion is essential for maintaining proper X-linked gene dosage in female hPSCs. While xenofree, feeder-dependent hESC medium minimizes erosion (Cloutier et al., 2022), prolonged culture may still increase vulnerability in these conditions. Targeting *XIST* promoter region by CRISPR-Cas9 gene editing can restore *XIST* expression and overcome erosion; however, this depends on the error-prone mechanism of non-homologous end joining (NHEJ) (Motosugi et al., 2022). XCI erosion can also be reversed by resetting hPSCs to a naive pre-XCI state, followed by a transition back to the primed state, but maintenance of XCI is limited to a certain number of passages (Patel et al., 2017; Agostinho de Sousa et al., 2023). Therefore, no current methods ensure permanent prevention or correction of XCI erosion.

X-linked gene activity of the Xe typically falls between the levels observed for Xa and the Xi (Bansal et al., 2021). In female naive hPSCs, besides the presence of two active X chromosomes (XaXa), these cells have a pronounced global decrease in DNA methylation levels and erasure of methylation marks at imprinted regions (Klobučar et al., 2020; Theunissen et al., 2016). While direct causality between the XaXa status and global demethylation has not yet been made for human PSCs, this association has been proven for female mouse XaXa ESCs (Choi et al., 2017). Interestingly, global DNA demethylation has been linked to advanced stages of XCI erosion in female hiPSCs (Bansal et al., 2021). Given the potential link between X-linked gene dosage and global DNA methylation, it is important to define how the degree of erosion impacts DNA methylation-dependent processes. This is particularly important for imprinted loci, which are often irreversibly dysregulated in hPSCs (Nazor et al., 2012; Bar and Benvenisty, 2019), with potentially impacting the functionality and fitness of their derivatives.

A key concern for hPSC clinical use is how XCI erosion affects differentiation. Research shows differences in differentiation ability and cell fate decisions between eroded and non-eroded hiPSCs. For instance, hiPSCs with no *XIST* expression exhibit more immature differentiation in teratoma assays (Anguera et al., 2012). Moreover, XCI erosion in hiPSCs influences neuronal maturation in cortical brain organoids (Motosugi et al., 2022) and cell fate decisions during cardiac differentiation (D’Antonio-Chronowska et al., 2019). XCI erosion also impacts the use of female hiPSCs with X-linked mutations for disease modeling by inadvertently activating the non-mutated allele on the Xi (Mekhoubad et al., 2012). It also remains unclear whether Xi erosion changes during differentiation, as studies show inconsistent findings on its maintenance, rescue, or amplification (Mekhoubad et al., 2012; Vallot et al., 2015; Patel et al., 2017; Motosugi et al., 2022; Cloutier et al., 2022).

Here, we explore the dynamics of erosion in a collection of female hiPSCs, including isogenic pairs with high levels (XIST+) or low levels of *XIST* (XIST−) expression. We employ RNA fluorescence *in situ* hybridization (FISH), Sanger sequencing, targeted amplicon-based allelic quantification, and RNA-seq to quantify allelic expression in these cells before and after differentiation. We unveiled that XCI erosion is frequent yet heterogeneous among our hiPSCs with no impact on the global (hydroxy)methylome and genomic imprinting. While not all X-linked genes are affected by erosion, specific features increase susceptibility, with escapees being particularly hypersensitive. Importantly, the variability of XCI status among hiPSCs persists through differentiation. Our findings emphasize the importance of drawing attention to XCI erosion within the stem cell community and advocate its inclusion in hiPSC quality control given their implications for their basic, translational, and clinical applications.

## Results

### Impaired XCI is frequent in female hiPSCs

**Figure 1:**
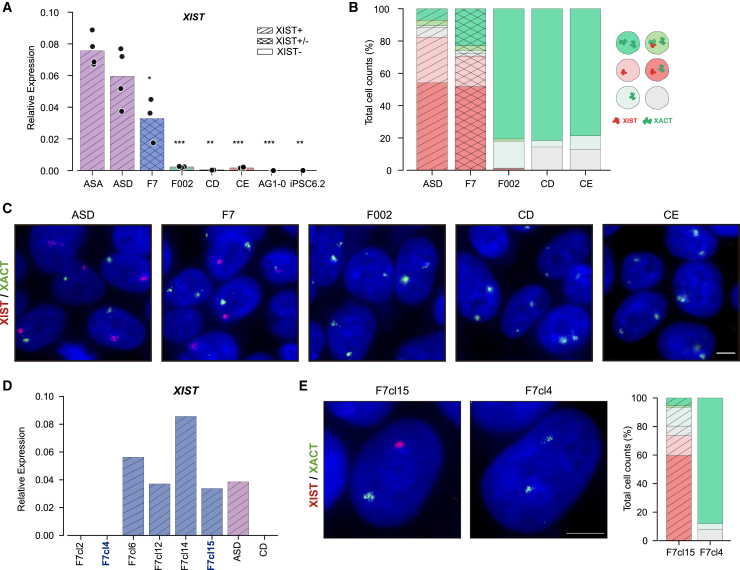
XCI status in female hiPSCs (A) Barplot showing RT-qPCR analysis of *XIST* expression normalized to *GAPDH* housekeeping gene in female hiPSCs. Bars represent the average *XIST/GAPDH* relative expression. ASA, ASD, and AG1-0: *n* = 4; F7, F002, and CE: *n* = 3; CD and iPSC6.2: *n* = 2. Statistically significant differences between all iPSCs and ASA iPSCs are indicated as ^∗^*p* < 0.01; **^∗∗^***p* < 0.001; **^∗∗∗^***p* < 0.0001 (unpaired two-tailed Student’s t test). (B) Graph represents percentage of cells with different expression profiles for XIST and XACT by RNA FISH as depicted in the legend on the left; a minimum of 200 cells were counted per cell line. (C) Representative RNA FISH images for XIST (red) and XACT (green) in ASD, F7, F002, CD, and CE hiPSCs; DNA stained in blue by DAPI; scale bar: 5 μm. (D) Barplot with RT-qPCR analysis of *XIST* expression normalized to GAPDH housekeeping gene in newly generated F7 cell lines (F7cl2, F7cl4, F7cl6, F7cl12, F7cl14, and F7cl15). XIST+ ASD and XIST− CD cell lines were used as positive and negative controls, respectively; *n* = 1 for all iPSCs. (E) On the left, representative RNA FISH images for XIST (red) and XACT (green) in isogenic F7cl15 and F7cl4 hiPSCs; DNA stained in blue by DAPI; scale bar: 5 μm; On the right, graph represents percentage of cells with different expression profiles for XIST and XACT by RNA FISH as depicted in (B); a minimum of 200 cells were counted per cell line.

Erosion of XCI can have a major impact in the downstream applications of hiPSCs, making it crucial to evaluate the stability of the Xi. To address this, we took advantage of our collection of female hiPSCs (Burridge et al., 2011; Chamberlain et al., 2010; Gomes et al., 2020; Pólvora-Brandão et al., 2018; Silva et al., 2020), which comprises eight cell lines spanning a wide range of cell passages (P), including two isogenic pairs, ideal for epigenetic studies (Table S1). We began by assessing *XIST* expression as a proxy for erosion using quantitative reverse-transcription PCR (RT-qPCR), revealing distinct expression patterns: high levels (XIST+) in ASA and ASD hiPSCs (P16–20), intermediate levels (XIST±) in the F7 line (P37–39), and residual or absent *XIST* expression (XIST−) in F002 (P41–45), CD (P21–25), CE (P16–20), AG1-0 (P86–90), and iPSC6.2 (P85–87) lines (Figure 1A). Cell passage significantly impacted *XIST* downregulation, the first sign of XCI erosion, but it was also observed in lower-passage lines like CD and CE. Unlike XIST+ ASD line, *XIST* loss in CD and F002 lines correlated with increased methylation levels at YY1 binding sites in XIST exon 1 (Figure S1A), a known *XIST* activator (Chapman et al., 2014; Fukuda et al., 2021; Makhlouf et al., 2014). To complement RT-qPCR analysis, we conducted single-cell RNA FISH for XIST and XACT. While XIST coats the Xi, XACT, another lncRNA, coats the Xa and Xe (Vallot et al., 2013, 2015). RNA FISH was performed on five cell lines: XIST+ (ASD), XIST− (F002, CD, and CE), and XIST± (F7). XIST was detected in >80% of XIST+ ASD, 70% of XIST± F7, and ∼0% of XIST− (F002, CD, and CE) hiPSCs, confirming RT-qPCR results (Figures 1B and 1C). Conversely, >80% of XIST− hiPSCs showed biallelic XACT expression, characteristic of erosion. XIST+ ASD showed a few cells with biallelic XACT in the presence (3%) or absence of XIST expression (7%), a percentage that increases to 25% in the XIST± F7 line (3% in the presence and 22% in the absence of XIST). Therefore, while XIST+ ASD is composed mostly of non-eroded cells, XIST± F7 presents a mixed population of non-eroded and eroded cells at roughly 75/25% ratio (Figures 1B and 1C).

Since epigenetic states are clonally propagated, we decided to isolate XIST+ and XIST− subclones from the F7 line. We picked 6 viable subclones and screened them for *XIST* expression by RT-qPCR to isolate 4 F7 XIST+ (F7cl6, F7cl12, F7cl14, and F7cl15) and two XIST− subclones (F7cl2 and F7cl4) (Figure 1D). We then selected one XIST+ (F7cl15) and one XIST− (F7cl4) subclones and analyzed the expression of *XIST* and *XACT* lncRNAs by RNA FISH (Figure 1E). As expected, *XIST* was detected in most cells in F7cl15, but not present in F7cl4 hiPSCs, while *XACT* was mostly monoallelic in F7cl15 and biallelic in F7cl4 hiPSCs (Figure 1E). In conclusion, our collection of hiPSC lines present different XCI states: ASD mostly preserves XCI and F002, CD, and CE hiPSCs show Xi erosion. The F7 line is a mosaic, containing both normal and eroded cells, from which XIST+ (e.g., F7cl15) and XIST− (e.g., F7cl4) subclones were successfully isolated.

While XCI loss reactivates some X-linked genes, the entire chromosome is not fully reactivated (Vallot et al., 2015; Theunissen et al., 2016; Bar and Benvenisty, 2019). Taking advantage of a panel of validated FISH probes targeting nascent transcripts, we investigated whether four X-linked genes, *HUWE1*, *ATRX*, *POLA1*, and *HDAC8*, are prone to reactivation upon XCI erosion. From these genes, we had prior knowledge that *POLA1* tends to be an eroded gene, while *ATRX* and *HUWE1* tend to be non-eroded genes (Vallot et al., 2015; Patel et al., 2017). The behavior of *HDAC8* was unknown at this stage. Our results show that *HUWE1*, *ATRX*, and *HDAC8* genes remain monoallelic in all hiPSC lines tested, regardless of XCI status (Figures S1B and S1C). This suggests that these genes are resistant to reactivation upon XCI erosion and continue to be expressed from the Xa. To further validate these findings, we took advantage of the common single-nucleotide polymorphism (SNP) rs3088074, present in the *ATRX* gene in all our hiPSC lines except for F002 (Figure S1D). We successfully showed that *ATRX* was always expressed from a single allele in each cell line not only confirming the monollelic expression of *ATRX* but also providing evidence of their clonal nature (Figure S1E). Interestingly, we observed that the isogenic CD and CE hiPSCs derived from the same skin biopsy express different *ATRX* alleles, indicating that they derived from somatic cells with a different Xi (Figure S1E). In contrast to *ATRX*, *HUWE1*, and *HDAC8*, *POLA1* exhibited a different behavior. In XIST+ cell lines, this gene shows monoallelic expression in most cells (ASD: 94%; F7cl15: 96%) while it was consistently biallelically expressed in XIST− cell lines, (F002: 93%, CD: 92% and CE: 82%). F7cl4 was an exception, with *POLA1* remaining mostly monoallelic (Figure S1C). These results suggest that *POLA1* is frequently reactivated from the Xe. Overall, our findings confirm the occurrence of XCI erosion in some of our female hiPSC lines. We also show that certain genes (*ATRX*, *HDAC8*, and *HUWE1*) resist this process, while others (*XACT* and *POLA1*) are more susceptible, with potential changes in the reactivation dynamics across different hiPSCs.

### Different patterns of XCI erosion in female hiPSCs

**Figure 2:**
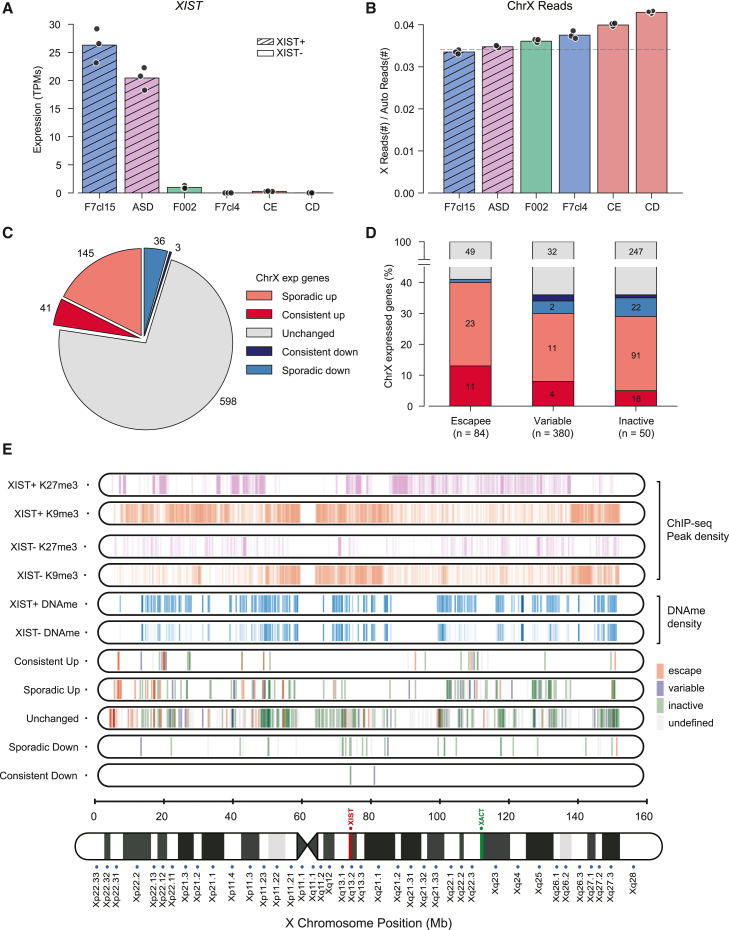
RNA-seq analysis reveals different degrees of XCI erosion in independent human iPSCs (A) *XIST* expression analysis by RNA-seq in F7cl15, ASD, F002, F7cl4, CE, and CD hiPSCs. The graph shows the transcripts per million (TPMs) expression values from biological triplicates (black dots) of each sample. (B) Barplot representing the ratio of normalized X chromosome/autosome reads by RNA-seq in F7cl15, ASD, F002, F7cl4, CE, and CD cell lines. The y axis shows the ratio between the number of chrX reads versus autosomal reads from the biological triplicates of each sample; dashed line represents the average ChrX/autosomal reads between the two XIST+ hiPSC lines, F7cl15 and ASD. Differences between XIST− and XIST+ lines (ASD and F7cl15) are all significant (t test *p* < 0.01 and Cohen’s d > 1). ASD vs. F7cl15 showed no statistical significance using the same standard. (C) Pie chart depicting the number of consistently upregulated, sporadic upregulated, consistently downregulated, sporadic downregulated, and unchanged X-linked genes in XIST− hiPSCs when compared to XIST+ hiPSCs. Consistently up/down-regulated genes: differentially expressed in 3 to 4 XIST− hiPSCs; sporadic up/down-regulated genes: differentially expressed in 1 to 2 XIST− hiPSCs. (D) Barplot illustrating the percentage of escape, variable, and inactive genes categorized according to Tukiainen et al., 2017; Werner et al., 2022; Tukiainen et al., 2017; and Werner et al., 2022, within consistently and sporadic upregulated, consistently and sporadic downregulated, and unchanged genes (a total of 823 expressed genes). (E) Distribution of H3K9me3 and H3K27me3 ChIP-seq peaks (Yokobayashi et al., 2021) and DNA methylation level (Bansal et al., 2021) in XIST+ and XIST− cell lines, along with the position of all genes marked as escapee, variable, inactive, or undefined in each category (consistently up/down, sporadic up/down, and unchanged) along the X chromosome.

To fully characterize the extent of XCI status, we performed bulk RNA sequencing (RNA-seq) on triplicates of our 2 XIST+ (F7cl15 and ASD) and 4 XIST− (F002, F7cl4, CD, and CE) hiPSCs. This includes isogenic pairs for XIST+ and XIST− (F7cl15 and F7cl4) and for XIST− (CD and CE) with distinct eroded X chromosomes. We first confirmed the expected pattern of *XIST* expression for each hiPSC line (Figure 2A) and that all hiPSC lines express high levels of pluripotency markers (Figure S2A). We next evaluated the ratio of RNA-seq reads mapping on the X versus autosomal reads (X:A ratio) as a proxy for erosion (Figure 2B). A statistically significant increase in the X:A ratio in XIST− hiPSCs was observed when compared to XIST+ hiPSC lines. Also, this increase varied substantially among different XIST− hiPSCs, ranging from 3.5% for the F002 (with residual *XIST* expression) to 24.6% for the CD (Figure 2B). To better understand how this variability translates at the gene level, we conducted pairwise differential gene expression (DGE) analysis by comparing the different XIST− against both ASD and F7cl15 XIST+ hiPSCs (FDR < 0.05; −0.33 > logFC > 0.33). The low logFC threshold was chosen to capture subtle changes in genes either reactivating or increasing in expression from the Xi upon XCI erosion. As anticipated, XIST− hiPSCs exhibited a higher number of upregulated X-linked genes when compared to both XIST+ hiPSCs, while no such trend was observed for autosomal genes (Figures S2B and S2C). Consistent with the X:A ratio variation, the number of upregulated X-linked genes varies in XIST− hiPSCs, with the CD line showing the highest number of overexpressed X-linked genes (Figures S2B and S2C). We also noted an elevated number of upregulated genes in ASD compared to F7cl15 (Figures S2B and S2C), implying that the ASD line may exhibit initial signs of erosion. This is consistent with the lower *XIST* levels (Figure 2A) and the increased number of cells with biallelic *XACT* observed by RNA FISH compared to the F7cl15 line (Figures 1B and 1C). Overall, these findings suggest that XIST− hiPSCs can exhibit distinct levels of XCI erosion consistent with previous reports (Bansal et al., 2021; Yokobayashi et al., 2021).

### Predictors of gene reactivation upon XCI erosion

We next investigated whether X-linked gene reactivation could be predicted upon XCI erosion. To classify gene behavior, we grouped X-linked genes by expression changes in XIST− vs. XIST+ hiPSCs. Genes upregulated in 3–4 lines were labeled “consistently upregulated,” while those upregulated in only 1–2 lines were classified as “sporadically upregulated.” Similarly, genes with decreased expression were categorized as “sporadically” or “consistently downregulated.” Genes with no consistent changes were deemed “unchanged.” This categorization identified 41 consistently upregulated, 145 sporadically upregulated, and 598 unchanged genes, with a few genes classified as consistently (3) or sporadically (36) downregulated, including *XIST* (Figure 2C and Table S2).

We first asked whether the original gene activity on the Xi prior to erosion could predict its upregulation upon *XIST* loss. For that, we categorized X-linked genes as inactive, variable (facultative escapees), or escape (constitutively escapees) genes according to Tukiainen et al. (2017) and reviewed by Werner et al. (2022). This categorization allowed us to examine whether pre-existing escape activity correlates with increased susceptibility to upregulation during XCI erosion. Among the 41 consistently upregulated genes, 34 could be categorized in these three classes (Figure S2D and Table S2). Of these, 35% (12 genes) were identified as escapees (e.g., *GYG2*, *NAP1L3*, and *TXLNG*). This proportion rises to 47% (16 genes) when variable genes are included. This represents a higher proportion compared to the percentage of escapees (∼15%) or the combined proportion of escapees and variable genes (∼25%) on the X chromosome. Consistently, nearly 40% of the escapees expressed in hiPSCs were either consistently or sporadically upregulated, while this proportion was lower for variable and inactive genes (Figure 2D). These findings indicate that escape genes, although partially evading *XIST*-dependent silencing on the Xi, are highly susceptible to upregulation upon the loss of *XIST*.

We next investigated how X chromosome positioning and epigenetic features, including histone marks and DNA methylation, influence X-linked gene upregulation in eroded hiPSCs. As observed in Figure 2E, consistently upregulated genes tend to localize in specific regions of the X chromosome, notably in the short arm (especially Xp22), as well as in the central portion of the long arm (Xq22 to Xq23). We compared these regions with Xi chromatin marks (H3K27me3 and H3K9me3) from chromatin immunoprecipitation sequencing (ChIP-seq) in XIST+ and XIST− hiPSCs (Yokobayashi et al., 2021) and found a predominant overlap between consistently upregulated genes and H3K27me3-rich domains (Figure 2E). To corroborate this observation, we plotted metagene profiles to visualize the distribution of H3K27me3/H3K9me3 marks across consistently upregulated, sporadically upregulated, and unchanged genes in XIST+ and XIST− hiPSCs. Consistently upregulated genes showed the highest H3K27me3 enrichment in XIST+ cells, followed by sporadically upregulated genes, while unchanged genes had the lowest enrichment. The differences between gene categories were statistically significant (Figure S2E; t test *p* < 0.01). Upon *XIST* loss, H3K27me3 was reduced for all genes as predicted. These data suggest that H3K27me3 enrichment in the Xi is a predictor of reactivation of X-linked genes upon erosion. In contrast, H3K9me3 did not seem to play a role in XCI erosion, in accordance with the literature (Vallot et al., 2015; Yokobayashi et al., 2021).

Next, we correlated loss of DNA methylation at promoters of X-linked genes with their categorization upon erosion. We leveraged DNA methylation data from Bansal et al. (2021) in XIST+ and XIST− iPSCs (see methods) to show pronounced loss of DNA methylation in consistently upregulated genes, moderate loss in sporadically upregulated genes, and minimal loss in unchanged genes (Figure S2F). The different behavior of each gene category was statistically significant (*p* = 0.028 for consistently vs. sporadic, *p* < 0.001 for sporadic vs. unchanged, using a t test).

Finally, given that escape genes are more prone to XCI erosion, we measure the distance of each gene to the closest upregulated escapee. We observed that consistently upregulated genes are significantly closer to these escapees compared to sporadically upregulated genes, which, in turn, are closer than unchanged genes (Figure S2G), suggesting a spatial influence on reactivation.

Overall, these findings confirm that X-linked genes vary in susceptibility to XCI erosion, with factors such as escapee status, proximity to escape genes, localization in Xp22 or Xq22-q23 regions, H3K27me3 enrichment, and reduced promoter methylation upon erosion significantly increasing the likelihood of erosion.

### Allele-specific analysis reveals increased Xi expression after erosion, including escape genes

**Figure 3:**
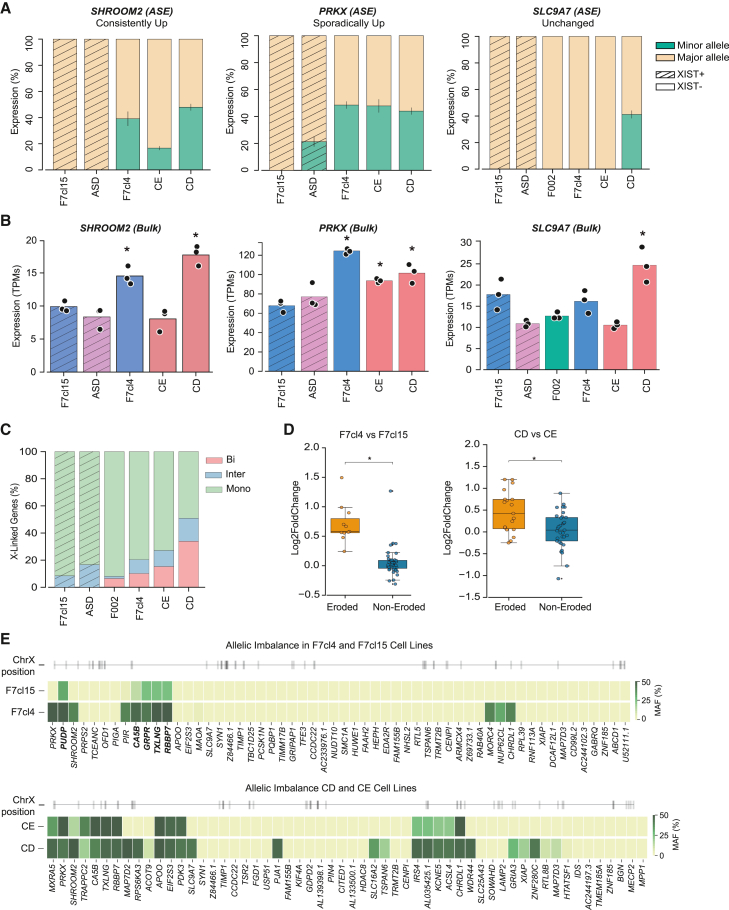
Eroded hiPSC lines show increasing number of biallelic gene expression (A) Allele-specific expression (ASE) based on RNA-seq analysis from representative genes containing common SNPs across different hiPSCs. Bar plots displaying the average percentage of expression ±SEM of the major and minor alleles for the X-linked genes *SHROOM2*, *PRKX*, and *SLC9A7* in triplicates of XIST+ (F7cl15, ASD) and XIST− (F002, F7cl4, CE, and CD) hiPSCs. (B) Corresponding RNA bulk expression based on RNA-seq analysis for these X-linked genes in the same XIST+ and XIST− hiPSCs shown in (A). Bar plots represent expression in TPMs (transcripts per million) for each gene in the different iPSC lines. Asterisks indicate statistical significance compared to either F7cl15 or ASD cell line (independent t test, *p* value < 0.05). (C) Percentage of biallelic (Bi), intermediate (In-btw), and monoallelic (Mono) genes in XIST+ (F7cl15 and ASD) and XIST− (F002, F7cl4, CE, and CE) hiPSCs. Classes were defined based on the minor allele frequency (MAF) (methods for details). (D) Differential gene expression analysis (DGEA) between F7cl4/F7cl15 and CD/CE isogenic pairs. Genes with at least 10% increase in their MAF were defined as eroded genes, while all the other expressed genes were considered non-eroded. Boxplots show the classification of each gene (eroded or non-eroded) against the log2 fold change obtained from the DGEA. Asterisks indicate statistical significance (*p* value < 0.01, Mann-Whitney U test). (E) Minor allele frequency within prominent X-linked genes in isogenic hiPSC pairs, F7cl15/F7cl4 and CD/CE. Heatmap shows genes containing common SNPs in both isogenic cell lines, ordered by their position along the X chromosome (gray lines in the ideogram).

To correlate X-linked gene overexpression with the reactivation of genes from the Xe, we assessed allele-specific expression (ASE) from our RNA-seq dataset. Alleles on the two X chromosomes were discriminated using SNPs identified through whole exome sequencing (WES) of the cell lines included in our study (methods for details). By assessing phased haplotypes of SNPs, we found around 60 expressed heterozygous X-linked genes in each cell line (Table S3), some of which were present across different XIST+ and XIST− hiPSC lines, except for the F002 cell line that consistently showed fewer overlapping informative SNPs compared to the other lines. Biological replicates from three consecutive cell passages demonstrated remarkably consistent ASE of the various X-linked genes, suggesting a stable XCI status across replicates (Table S3). The original gene activity on the Xi in our XIST+ hiPSCs did not always align with the classifications from Tukiainen et al. (2017) and Werner et al. (2022) (Table S2). Notable examples included *CHRDL1* (here classified as inactive instead of escapee), *RBBP7* (here classified as escapee instead of inactive), and *PRKX* and *EIF2S3* (here classified as escapee or inactive depending on the XIST+ hiPSC line, rather than exclusively escapee) (Figures 3A and S3A). These minor discrepancies may result from the classification applied not being hiPSC specific.

In Figure 3A, we show some examples of X-linked genes with informative SNP across different cell lines. For instance, *SHROOM2,* a consistently upregulated gene, is expressed from only one allele in XIST+ cells but from both alleles in XIST− lines (Figure 3A, left panel). Similarly, *PRKX*, a sporadically upregulated gene, is monoallelic or allele biased in XIST+ cell lines (F7cl15 and ASD, respectively) but becomes equally expressed from both X chromosomes in eroded cell lines (Figure 3A, center panel). On the other hand, *SLC9A7*, an unchanged gene, is only reactivated in the most eroded CD cell line (Figure 3A, right panel). Importantly, the reactivation of the Xi is accompanied by an overall increased expression of these genes, as shown by bulk RNA-seq analysis (Figure 3B).

In general, the allelic expression analysis revealed four distinct behaviors of X-linked genes during erosion: (1) inactive genes in XIST+ cells that become biallelically expressed in all XIST− cells (e.g., *CHRDL1* and *SHROOM2*); (2) genes that are only re-expressed in the CD line (e.g., *SLC9A7*, *XIAP*, *TSPAN6*, and *MAP7D3*); (3) genes that remain monoallelically expressed across all cell lines (e.g., *CENPI* and *TRMT2B*); and (4) escape genes exhibiting biased allelic expression in at least one of the two XIST+ lines, which transition to balanced biallelic expression in XIST− cells (e.g., *PRKX*, *TXLNG*, *RBPP7*, and *EIF2S3*) (Figures 3A and S3A).

To further examine the behavior of escape genes, we conducted RNA FISH for three X-linked escapees (*TXLNG*, *RBBP7*, and *EIF2S3*) in two XIST+ hiPSC lines (F7cl15 and ASD) and two XIST− lines (F7cl4 and CD), enabling single-cell allele-specific analysis. Specific probes for each escape gene were combined with *XACT* as a marker of both Xa and Xe and *HUWE1* as a non-eroded X-linked gene marking the Xa. *XACT* was used instead of *XIST* since XIST− hiPSCs do not express *XIST*, whereas *XACT* would be expressed in both cell lines, mono- or biallelically. This experimental setup allowed us to categorize cells into four classes based on ASE of each escapee (Figures S3B and S3C): biallelic, monoallelic from the Xi/Xe, monoallelic from the Xa, and non-expressing cells. For all escapees, biallelic expression increased in XIST− hiPSCs, except for *EIF2S3* in F7cl15/F7cl4, which remained monoallelic from the Xa as expected from RNA-seq data (Figures S3A and S3C). Moreover, the bias toward monoallelic Xa expression seen in XIST+ cell lines was, to a certain extent, also attenuated in XIST− hiPSCs. In summary, these findings show that *XIST* loss in eroded cells promotes increased expression of escape genes from the Xe, suggesting that XIST plays a key role in limiting their expression on the Xi.

To analyze allelic expression globally, we divided X-linked genes in three classes based on the minor allele frequency (MAF), which represents the frequency of the less common allele in each cell line: monoallelic (MAF ≤ 0.10), biallelic (MAF ≥ 0.40), and intermediate (0.10 < MAF < 0.40). As expected, the XIST+ cell lines (ASD and F7cl15) showed mostly monoallelic expression, with no biallelic expression (Figure 3C). Consistent with the increased X:A ratio observed in eroded hiPSCs (Figure 2B), we detected a higher number of intermediate and biallelic genes in eroded hiPSC lines, in the following order: F002 < F7cl4 < CE < CD. These findings suggest that the overexpression of X-linked genes in eroded hiPSCs is driven by the enhanced transcriptional activity from the Xe.

Next, we took advantage of our isogenic pairs (F7cl4/F7cl15 and CD/CE), which share the same SNPs, to conduct a more in-depth allele-specific analysis. To achieve this, we classified as “eroded genes” those showing at least a 10% increase in expression between the less eroded (F7cl15 and CE) and the most eroded line (F7cl4 and CD), while the remaining genes were classified as “non-eroded” (Figure 3D). Our results show that eroded genes exhibit higher bulk expression compared to non-eroded genes. This is manifested not only in XIST− F7cl4 vs. XIST+ F7cl15 cell lines but also in the CD vs. CE cells lines, which differ in their levels of erosion (Figure 3D). Not surprisingly, increased gene activity in the Xe maps to the short arm and the central portion of the long arm (Xq22 to Xq23) (Figure 3E), as previously observed for the overexpressed genes (Figure 2E). Notably, all escapees with available SNPs in XIST+ F7cl15 line (*PUPD*, *CA5B*, *GRPR*, *TXLNG*, and *RBBP7*) transitioned from an unequal allelic pattern to a more balanced allelic expression in XIST− F7cl4 hiPSCs (Figure 3E), in line with the previous observations. In summary, these findings demonstrate that the elevated gene expression in eroded hiPSC lines is primarily attributed to the increased transcription from the former Xi, with this effect intensifying with the degree of erosion, in accordance with previous studies (Mekhoubad et al., 2012; Vallot et al., 2015; Bar and Benvenisty, 2019; Bansal et al., 2021). Importantly, among the various genes on the X chromosome, escape genes are particularly sensitive to XIST loss, with their expression being upregulated upon XCI erosion. These findings highlight the critical role of XIST in restricting their expression from the Xi.

### No impact of XCI erosion in (hydroxy)methylation or genomic imprinting

The dosage of X-linked genes is closely linked to overall levels of DNA methylation as shown for mouse ESCs (Choi et al., 2017). This association might also apply to hPSCs as these cells in naive conditions have two active X chromosomes and a global decrease in DNA methylation levels. Hence, given that XCI erosion leads to partial reactivation of the Xi, it is pertinent to evaluate DNA methylation levels in eroded hiPSCs. For this analysis, we have expanded our original cohort of hiPSCs with 7 new female iPSC lines from 3 different donors that were generated in the course of this study (Table S4) (Silva et al., 2021a, 2021b). We used the expression levels of *XIST* as a proxy for the XCI status, having the XIST± F7 line as a reference to separate what we classified as XIST+ and XIST− hiPSC lines. Out of the 7 new clones we have derived, 2 were classified as XIST+ and the remaining 5 as XIST− (Figure S4A). This includes hiPSC lines with low passage number (<P15) (Figure S4A and Table S4). We confirm the XCI status at a single-cell level by evaluating one of the XIST+ line (2042cl9) and one of the XIST− (2042cl1) hiPSC lines by RNA FISH for *XIST* and *XACT* (Figure S4B). From the initial set of XIST+ and XIST− female hPSC lines, we expanded to include three isogenic pairs, enhancing the robustness of our DNA methylation analysis (Tables S1 and S4).

**Figure 4:**
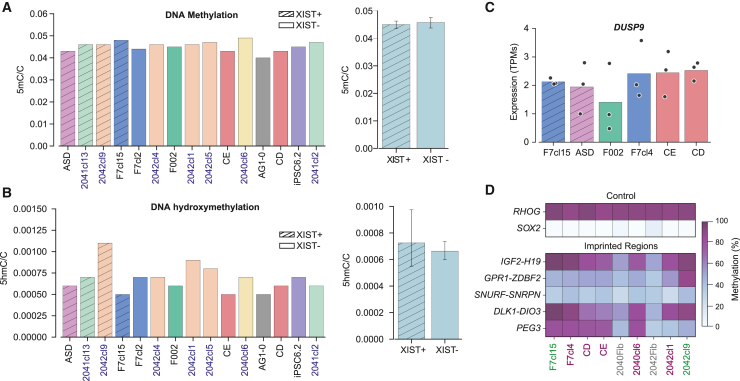
XCI erosion in our hiPSCs shows no impact on global DNA (hydroxy)methylation and genomic imprinting (A and B) Barplots showing global 5mC (A) and 5hmC (B) levels measured by liquid chromatography-tandem mass spectrometry (LC-MS/MS). On the left, graph represents the ratio of 5mC or 5hmC per total cytosines in XIST+ (ASD, 2041cl13, 2042cl9, and F7cl15) and XIST− (F7cl2, 2042cl4, F002, 2042cl1, 2042cl5, CE, 2040cl6, AG1-0, CD, iPSC6.2, and 2041cl2) hiPSCs; *n* = 1 for all iPSCs; On the right, barplot shows the average ratio of 5mC or 5hmC ± SD per total cytosines ± SEM in XIST+ versus XIST− hiPSC lines. (C) *DUSP9* expression data by RNA-seq in F7cl15, ASD, F002, F7cl4, CE, and CD hiPSCs. The graph shows the transcripts per million (TPMs) expression values from biological triplicates (black dots) of each sample. (D) Heatmap representing the percentage of DNA methylation of the control loci (*RHOG* and *SOX2*) and imprinting control regions of several imprinted regions (*IGF2*-*H19*, *GPR1*-*ZDBF2*, *DLK1*-*DIO3*, *PWS*/*AS*, and *PEG3*) for F7cl15, F7cl4, CD, CE, 2040cl6, 2042cl1, and 2042cl9 iPSCs as well as 2040 and 2042 fibroblasts (fib). XIST+ and XIST− lines are colored in green and purple, respectively. Fibroblasts are colored in gray.

To explore the potential variations in global DNA methylation levels between XIST+ and XIST− hiPSCs, we measured global 5-methylcytosine (5mC) and 5-hydroxymethylcytosine (5hmC) levels by liquid chromatography-tandem mass spectrometry (LC-MS/MS). No association between *XIST* expression and 5mC or 5hmC levels was observed in our cohort of female iPSCs, even for high-passage cells (Figures 4A and 4B). In line with this, our RNA-seq data showed no discernible differences in the expression levels of the *DUSP9* between XIST+ and XIST− cells, a gene previously identified as a pivotal X-linked factor influencing DNA methylation levels (Bansal et al., 2021; Choi et al., 2017) (Figure 4C). In conclusion, our study demonstrates that XCI erosion within our panel of female hiPSCs exerts no discernible influence on DNA (hydroxy)methylation levels.

An epigenetic anomaly frequently detected in hiPSCs, but varying across hiPSC lines, is imprinting defects (Bar et al., 2017, p. 2020; Klobučar et al., 2020). To investigate whether XCI erosion contributes to the heterogeneity of imprinting defects, we utilized IMPLICON, a targeted amplicon-seq method, to measure DNA methylation at imprinted regions (Klobučar et al., 2020; Arez et al., 2022) in two sets of isogenic XIST+ and XIST− hiPSCs, including the isogenic pairs CE and CD, which exhibit varying degrees of XCI erosion. The presence or absence of *XIST*, as well as the degree of erosion, does not affect methylation patterns at imprinted regions (Figure 4D). Indeed, abnormal gain of DNA methylation at *DLK1-DIO3*, *PEG3*, or *IGF2-H19* imprinted regions in hiPSCs, which is not seen in their somatic cells of origin (2042 and 2040 fibroblasts, 2042 and 2040 Fib), occurred independently of *XIST* expression (Figure 4D and Table S5). In conclusion, as for the global levels of DNA (hydroxy)methylation, XCI erosion does not impact the methylation-dependent phenomenon of genomic imprinting in our iPSC lines.

### Persistent XCI erosion throughout female iPSC trilineage specification

**Figure 5:**
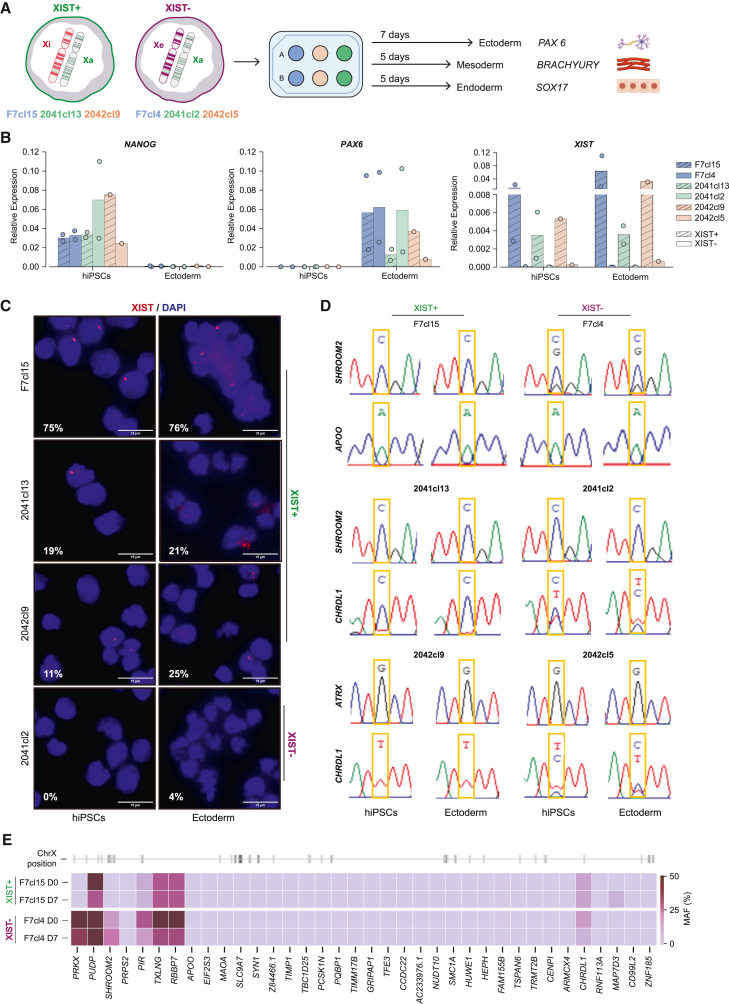
Erosion pattern persists upon ectodermal commitment (A) Illustration of the experimental design used for trilineage commitment of the three XIST+/XIST− isogenic hiPSC pairs (F7cl15 & F7cl4, 2041cl13 & 2041cL2, and 2042cL9 & 2042cL5) into ectoderm, mesoderm, and endoderm. The inactive X chromosome (Xi) is marked in red, the active X chromosome (Xa) is marked in green, and the eroded X chromosome (Xe) is marked in purple. (B) RT-qPCR analysis for *NANOG* (pluripotency marker), *PAX6* (neuronal marker), and *XIST* (Xi marker) normalized to *GAPDH* housekeeping gene in F7cl15, F7cl4, 2041cl13, 2041cl2, 2042cl9, and 2042cl5 hiPSCs and after ectoderm differentiation. Barplots represent the mean relative expression of *n* = 2 for all samples, except 2042cl9 and 2042cl5 in both hiPSCs and ectoderm (*n* = 1). (C) Representative images of XIST RNA-FISH and respective percentages of cells expressing *XIST* (red dots) in F7cl15, 2041cl13, 2042cl9, and 2041cl2 at D0 (hiPSCs) and D7 (differentiated ectodermal cells). The nuclei are counterstained with DAPI (blue). Scale bars represent 15 μm. Number of cells counted: hiPSCs - F7cl15: 281, 2041cl13: 173, 2042cl9: 194, 2041cl2: 94; ectoderm cells - F7cl15: 324, 2041cl13: 326, 2042cl9: 367, 2041cl2: 381. The values represent 1 independent experiment. (D) Allelic expression assayed by RT-PCR followed by Sanger sequencing resourcing to informative SNPs to distinguish the two alleles. The chromatograms represent illustrative examples of the allelic expression of heterozygous X-linked genes in each XIST+/XIST− isogenic hiPSC pair at D0 (hiPSCs) and at D7 (differentiated ectodermal cells): *SHROOM2* and *APOO* gene for the F7cl15 and F7cl4, *SHROOM2* and *CHRDL1* gene for 2041cl13 and 2041cl2, and *ATRX* and *CHRDL1* gene for 2042cl9 and 2042cl5. *CHRDL1* is not expressed in mesoderm cells. (E) Minor allele frequency (MAF) in prevalent X-linked genes between naive (D0) and differentiated (D7) cell states in F7cl15 (XIST+) and F7cl4 (XIST−) cell lines. Heatmap shows genes containing common SNPs in both states, ordered by their position along the X chromosome (gray lines in the ideogram).

Next, we explored the dynamics of Xe during differentiation. We first conducted trilineage commitment experiments in three isogenic pairs of XIST+ and XIST− iPSCs: F7cl15 & F7cl4, 2041cl13 & 2041cl2, and 2042cl9 & 2042cl5. These hiPSC lines were differentiated into ectoderm, mesoderm, and endoderm for 5 to 7 days (Figure 5A). As expected, all hiPSC lines expressed the neuronal marker *PAX6*, while downregulating the pluripotent marker *NANOG* during ectoderm differentiation (Figure 5B). Likewise, all lines expressed *BRACHYURY* upon differentiation to mesoderm (Figure S5A). For the endoderm, we saw a tendency for the *SOX17* marker to be less expressed in XIST− hiPSCs, with the XIST− 2042cl5 even failing to express it (Figure S5A). Notably, *NANOG* expression remained detectable in all cell lines after endoderm specification (Figure S5A), suggesting suboptimal differentiation toward this lineage with this protocol. Overall, taken together, these data suggest that XCI erosion does not prevent trilineage specification.

We next evaluated *XIST* expression before and after trilineage commitment by RT-qPCR and RNA FISH. After 4–6 cell passages, XIST+ 2041cl13 and 2042cl9 hiPSC lines have undergone substantial erosion, reflected by a reduction in the number of XIST+ cells detected by RNA FISH (19% for 2041cl13 and 11% for 2042cl9) (Figures 5B and 5C). In contrast, XIST− hiPSCs maintained residual expression levels of *XIST* by both RT-qPCR and RNA FISH, while F7cl15 remained as a bona-fide XIST+ hiPSC line (∼75% of XIST+ cells by RNA-FISH) (Figures 5B and 5C). Importantly, although the expression levels of *XIST* may rise during ectoderm differentiation, the number of *XIST+* cells remained stable and was not rescued in XIST− hiPSC lines (Figures 5B, 5C, S5A, and S5B). Overall, XIST+ cell numbers remain stable during iPSC commitment to the three germ layers, regardless of their initial proportions. We then addressed the ASE of several X-linked genes in the differentiated progeny of these hiPSC lines expressing diverse levels of *XIST*. For that, we identified eight X-linked genes with common SNPs, including 6 in consistent and sporadic eroded genes (Table S6) and qualitatively assessed allelic expression using Sanger sequencing. Overall, the *XIST*+ counterpart of each isogenic pair retains more genes with monoallelic expression (Figures 5D and S5C and Table S6). Despite considerable loss of *XIST*, most of the assessed genes in the XIST+ 2041cl13 and 2042cl9 lines are not detected as biallelic in contrast to their XIST− counterparts. Strikingly, the original allelic expression profile found for each gene in hiPSCs, irrespective of their *XIST* expression profile, is maintained upon commitment to the three germ layers (Figures 5D and S5C, and Table S6). These results were also recapitulated for ectodermal differentiation of the XIST+ ASD and XIST− F002, CE, and CD hiPSC lines (Table S6).

To investigate the allelic evolution of the eroded state during differentiation in greater detail, we performed RNA-seq for ectodermal differentiation of the XIST+/XIST− F7cl15/F7cl4 pair. As anticipated, the expression levels of pluripotent markers (*NANOG*, *POU5F1*, and *PRDM14*) decreased upon differentiation, while ectoderm markers (*PAX6*, *NES*, and *OTX2*) increased (Figure S5D). Notably, *XIST* expression increased throughout differentiation in the F7cl15 line but remained absent in the F7cl4 line (Figure S5E). We compared allelic expression in F7cl15 and F7cl4 at D0 and D7 and found that biallelic genes retained their expression pattern, including eroded genes like *PRKX* and *SHROOM2* in XIST− F7cl4 (Figure 5E). Additionally, the proportion of biallelic, monoallelic, and “intermediate” genes remained mostly stable from D0 to D7, with a slight decrease in biallelic expression (Figure S5F). Overall, erosion patterns were neither rescued nor magnified during germ layer commitment.

### Persistent XCI erosion throughout female iPSC cardiac differentiation

**Figure 6:**
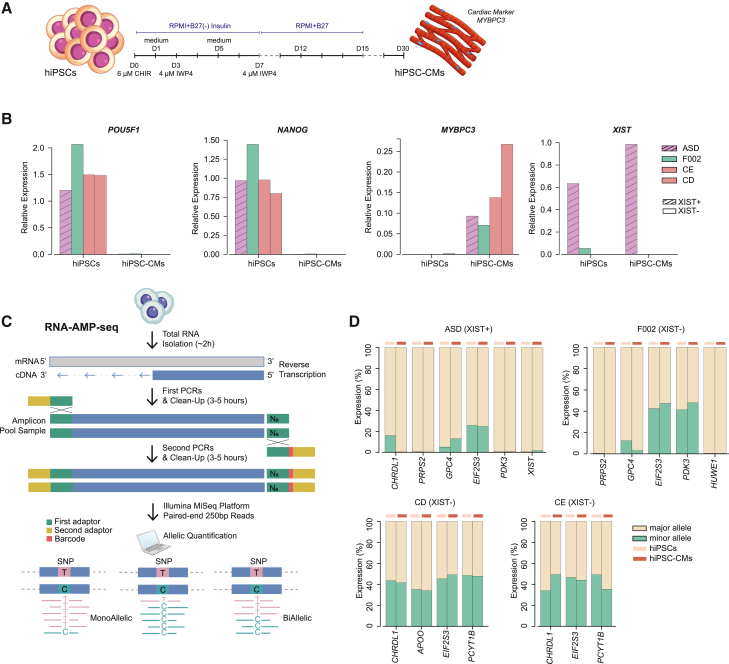
RNA-AMP-seq reveals maintenance of allelic ratio of X-linked genes upon cardiac differentiation of female hiPSCs (A) Schematic overview of the cardiac differentiation protocol (see methods for details). (B) Barplots with RT-qPCR analysis for *MYBPC3*, *NANOG*, *OCT4*, and *XIST* expression normalized to the *U6* housekeeping gene in ASD, F002, CE, and CD in hiPSCs and hiPSC-derived cardiomyocytes (hiPSC-CMs); *n* = 1 for all the samples. (C) Illustration of our novel RNA-AMP-seq methodology. A first PCR amplifies each region per sample in individual reactions in the presence of adapter sequences and 8 random nucleotides (N8) for downstream deduplication of the data. After cleanup using AMPure XP magnetic beads, a second PCR completes a sequence-ready library with sample barcodes for multiplexing. Libraries were sequenced using the Illumina MiSeq platform to generate paired-end 250 bp reads (methods for more details). (D) Allele-specific expression (ASE) based on RNA-AMP-seq analysis from representative genes containing common SNPs across different hiPSCs. Bar plots displaying the expression of the major and minor alleles for different X-linked genes in XIST+ (ASD) and XIST− (F002, CE, and CD) hiPSCs and hIPSC-CMs.

In addition to the short-term trilineage specification, we conducted a long-term cardiac differentiation protocol (Lian et al., 2012) on ASD, F002, CD, and CE lines (Figure 6A). After 15 days of differentiation, both XIST+ (ASD) and XIST− (F002, CD, and CE) cell lines were able to differentiate in contractile cardiomyocytes (Video S1). On day 30, we used RT-qPCR to confirm pluripotency marker downregulation (*POU5F1* and *NANOG*) and cardiac marker upregulation (*MYBPC3*) in all lines (Figure 6B). As expected, *XIST* remained expressed in ASD XIST+ cardiomyocytes and absent in XIST− hiPSCs (Figure 5B). To assess ASE of X-linked genes during cardiac differentiation, we developed a quantitative method, inspired by our prior work with IMPLICON (Klobučar et al., 2020). Our approach, named RNA-amplicon-sequencing (RNA-AMP-seq), integrates cDNA synthesis with amplicon sequencing and incorporates a de-duplication step to generate datasets with robust coverage and precise allelic discrimination for quantifying expression of targeted genes (methods for details). We focused on 9 X-linked genes with common SNPs in the population, including 4 consistently upregulated genes (*CHRDL1*, *GPC4*, *PDK3*, and *PCYT1B*), 1 sporadically upregulated (*APOO*), 2 unchanged (*PRPS2* and *HUWE1*), 1 consistently downregulated (*XIST*), and 1 escapee (*EIF2S3*) (Table S7). First, we validated this method by showing the enhanced sequence coverage offered comparing with RNA-seq in the same hiPSCs lines (Table S7). Next, we employed this method to assess the variation in allelic expression of X-linked genes with heterozygous SNPs between iPSCs and their cardiomyocyte derivatives (hiPSC-CM). Remarkably, allelic expression ratios of X-linked genes remain stable in both XIST+ and XIST− hiPSCs upon differentiation (Figure 6D). Our results show that XCI erosion remains stable even after prolonged differentiation into functional cell types, consistent with our findings with the short-term trilineage differentiation and other distinct differentiation paradigms (Patel et al., 2017; Motosugi et al., 2022).

Video S1. Representative video of contractile iPSC-derived cardiomyocytes at day 15 of differentiation

## Discussion

Our comprehensive exploration of XCI erosion in hiPSCs reveals four significant characteristics of this recurring phenomenon in female stem cell cultures: (1) XCI occurs frequently but exhibits high heterogeneity; (2) the likelihood of gene reactivation is influenced by the original gene activity in the Xi, its genomic location, and epigenetic environment; (3) partial reactivation of the Xe does not usually induce changes in global DNA (hydroxy)methylation levels or affect the epigenetic profile of imprinted regions; and (4) the heterogeneous status of the Xe remains in differentiated derivatives of hiPSCs.

We observed that XCI erosion is common in female hiPSC cultures, with varying degrees observed across different cell lines. Our iPSCs were grown in mTSER Plus medium, which might not provide protection against erosion (Cloutier et al., 2022). Nevertheless, XCI erosion is observed in any of the most commonly used human stem cell media, including StemFlex, E8, or classical hESC medium under an irradiated feeder layer (Mekhoubad et al., 2012; Vallot et al., 2015; Motosugi et al., 2022; Agostinho de Sousa et al., 2023). Therefore, our observation that XCI erosion is common in female hiPSCs aligns with previous findings performed using other medium formulations. In addition to demonstrating the prevalence of XCI erosion, our data reveal their remarkable heterogeneity. This observation aligns with recent research (Bansal et al., 2021; Yokobayashi et al., 2021), yet we provide clear confirmation through ASE analysis. The variability observed underscores that XCI erosion encompasses a spectrum of states, adding complexity to this phenomenon. Although not comprehensively addressed by us in this study, the variable nature of XCI erosion might significantly impact downstream applications in both research and clinical settings. This underlines the critical need to incorporate XCI erosion assessment into routine hiPSC quality control protocols.

Despite the heterogeneity in XCI erosion, our study identifies specific traits influencing the likelihood of gene reactivation from the Xi. Location of a gene in the Xp22 or Xq22-q23 chromosomal regions, inclusion within H3K27me3-enriched domains, whether it is an escape gene or near one, and the loss of DNA methylation at promoters upon erosion all collectively increase the likelihood of reactivation, as previously reported (Vallot et al., 2015; Bar and Benvenisty, 2019; Bansal et al., 2021; Topa et al., 2024). This is reflected in our categorization of genes based on the consistency of their upregulation across different XIST− relative to XIST+ hiPSClines. This classification allowed us to assess the impact of XCI erosion and distinguish genes that are more prone to reactivation from those with stable silencing. The “consistently upregulated” genes likely represent loci that are particularly susceptible to XCI erosion, while “sporadically upregulated” genes may reflect variability in the extent of erosion or differences in the epigenetic landscape between cell lines. “Unchanged” genes highlight loci with robust silencing mechanisms that appear resistant to XCI erosion. Naturally, our classification captures the variability in gene behavior across the hiPSC lines included in our collection. It is possible that different cell lines might yield slightly different results. Nevertheless, this framework provides key insights into XCI erosion heterogeneity.

A noteworthy result of our study is the increased sensitivity of escape genes to XCI erosion. While initially counterintuitive, as these genes are known to escape silencing on the Xi, our allele-specific analysis shows a shift in escape gene behavior in XIST+ vs. XIST− hiPSCs. These genes, mostly showing biased expression from one allele in XIST+ cells, transitioned to equal expression from both alleles in XIST− cells. We demonstrate this through allele-specific RNA-seq analysis (Figures 3A and S3A) and validate it with single-cell RNA FISH for three escapees (Figures S3B and S3C). Consistent with a recent report linking escape gene susceptibility to *XIST* loss and XCI erosion (Topa et al., 2024), our findings provide the first single-cell evidence of heightened escape gene vulnerability to XCI erosion. This suggests that XIST lncRNA normally restricts escape gene expression on the Xi when compared to the Xa. Interestingly, a recent study also demonstrated preferential transcriptional upregulation of escape genes following conditional deletion of *Xist* in mouse embryonic fibroblasts and hematopoietic stem and progenitor cells (Yang et al., 2022). Both our findings and these reports support the notion that escape genes are attenuated in expression levels on Xi. This parallels recent observations in naive hESCs, where XIST dampens gene expression at the chromosomal scale (Alfeghaly et al., 2023; Dror et al., 2024). A key question for future studies is whether the XIST-dependent attenuation of gene expression in these two phenomena shares the same molecular mechanisms.

Our study also revealed that XCI erosion does not globally impact DNA methylation and hydroxymethylation levels or alter genomic imprinting patterns in hiPSCs. This was an important issue since naive hPSCs or XaXa mouse ESC/iPSCs have global hypomethylation and erasure of imprints (Theunissen et al., 2016; Klobučar et al., 2020; Arez et al., 2022). Despite the reactivation of specific X-linked genes, the overall (hydroxy)methylation profiles remained unchanged between XIST+ and XIST− hiPSC lines. In a previous study, while most eroded hiPSCs retained global DNA methylation levels comparable to XIST+ hiPSCs, advanced stages of XCI erosion in female hiPSCs were associated with global DNA demethylation (Bansal et al., 2021). This demethylation was linked to upregulation of the *DUSP9* gene, a key factor in explaining how X chromosome dosage impacts global methylation (Choi et al., 2017; Bansal et al., 2021). However, in our XIST− hiPSCs, even at high passages (Figure 4C), *DUSP9* was never found to be eroded, which may explain the absence of global DNA methylation changes (Figure 4A). Furthermore, recent findings, including a study analyzing 165 female hiPSCs, found no evidence of biallelic *DUSP9* expression (Topa et al., 2024). Based on these observations, we speculate that global DNA demethylation is a rare consequence of XCI erosion, likely occurring only under specific conditions. We also explored whether XCI erosion could contribute to the methylation variation at imprinted loci in hiPSCs (Bar and Benvenisty, 2019; Klobučar et al., 2020) and found no evidence that it affects DNA methylation patterns at these genes Therefore XCI erosion did not contribute for the imprinting errors observed in our hiPSCs.

Previous studies showed inconsistent findings showing either a rescue (Vallot et al., 2015) or maintenance of the XCI erosion pattern upon iPSC differentiation (Patel et al., 2017; Motosugi et al., 2022). These inconsistencies may be explained by the fact that no ASE was employed in these studies or was limited to a small number of genes. Our ASE analysis on different differentiation paradigms using isogenic sets of XIST+ and XIST− hiPSCs clearly point to the maintenance of the reactivated state of eroded genes during differentiation with a few exceptions. Therefore, the heterogeneous abnormal XCI states in female hiPSCs persist in their differentiated derivatives, potentially affecting cellular functionality and fitness (D’Antonio-Chronowska et al., 2019; Motosugi et al., 2022), though this was not explored in our study. For our ASE analysis, we used a qualitative method, Sanger sequencing, and two quantitative methods: RNA-seq and RNA-AMP-seq. While RNA-seq gives a comprehensive overview of the transcriptome, RNA-AMP-seq targets specific genetic variants on genes and builds datasets with allelic discrimination and higher coverage (Table S7). RNA-AMP-seq demonstrates promise as a high-throughput method for erosion screening. Compared to our current approach, which analyzes 9 X-linked genes (5 consistently/sporadically upregulated) across 8 samples, RNA-AMP-seq offers significant scalability to accommodate a much wider range of genes and samples. Additionally, the design of new primer pairs allows for straightforward customization, enabling the application of RNA-AMP-seq to other categories of monoallelically expressed genes. This includes genes subjected to imprinting or exhibiting random monoallelic expression.

In conclusion, our study advances the current understanding of XCI erosion in female hiPSCs and its implications for stem cell biology and regenerative medicine. By characterizing the frequency and persistence of XCI erosion, we provide valuable insights that can inform the development of improved hiPSC culture and differentiation protocols. Moving forward, continued research into the molecular mechanisms and the culture conditions driving XCI erosion will be essential for optimizing the utility of hiPSCs in various biomedical applications.

## Methods

A more detailed version of this section is available in the supplemental information.

### Ethics

hiPSC lines were purchased or previously generated by us (Pólvora-Brandão et al., 2018; Silva et al., 2021a, 2021b) (Tables S1 and S4). Donor consent was obtained, and ethics approval granted by the Lisbon Academic Medical Center (Approval: 535/12, 170/18).

### Stem cell culture

hiPSC lines were maintained in mTeSR Plus medium (STEMCELL Technologies) on Matrigel-coated plates (Corning), with media changes every 24–48 h. Cells were passaged using 0.5 mM EDTA (Invitrogen) in 1x PBS (Gibco) and cultured at 37°C in a 5% CO_2_ incubator. Cells were dissociated with 0.5 mM EDTA/PBS, collected in washing medium (DMEM-F12, KSR, NEAA, L-Glutamine, β-mercaptoethanol), centrifuged, and resuspended in freezing medium (90% KSR, 10% DMSO) before storage in liquid nitrogen. iPSC cultures were routinely tested for mycoplasma contamination using the qPCR mycoplasma test (Mycoplasmacheck, Eurofins Genomics).

### RT-qPCR

Total RNA was extracted from all hiPSC lines using NZYol RNA isolation reagent (NZYTech) and treated with DNase I (Roche) to remove contaminating DNA, following the manufacturer’s instructions. DNase I-treated RNA (500 ng) was reverse-transcribed using random primers and a high-capacity cDNA reverse-transcription kit (Applied Biosystems). RT-qPCR was performed using NZYSpeedy qPCR Green Master Mix ROX (NZYTech) on StepOne or ViiA 7 real-time PCR systems (Applied Biosystems). Reactions were conducted in technical duplicates or triplicates and normalized to *GAPDH*. Primers are listed in Table S8. Data analysis was performed using StepOne or QuantStudio software, with relative gene expression calculated using the 2^−ΔΔCT^ method.

### PCR/RT-PCR followed by Sanger sequencing

To verify the presence of a specific SNP in ASD, F7, F002, CD, CE, 2041cl13, 2041cl2, 2042cl9, and 2042cl5 hiPSCs, genomic DNA was extracted using phenol:chloroform:isoamyl alcohol (Invitrogen) and amplified by PCR using primers listed in Table S8. For relative allelic expression analysis of X-linked genes in hiPSCs and their ectodermal, mesodermal, or endodermal derivatives, cDNA synthesized as described in the RT-qPCR section was amplified using the same primers. PCR products (DNA or cDNA) were purified with the NZYGelpure kit (NZYTech) and sent for Sanger sequencing to STAB VIDA. Data were analyzed and visualized using Chromas v.2.6.2 software.

### RNA FISH

The templates used for probe production were the following: XIST, a plasmid containing the 10 Kb exon 5–6 plasmid (Rosspopoff et al., 2023); *XACT*: RP11-35D3 bacterial artificial chromosome (BAC); *ATRX*: RP11-42M11 BAC; *HUWE1*: RP11-155O24 BAC; *HDAC8*: RP11-1021B19 BAC; *POLA1*: RP11-1104L9 BAC; *EIF2S3*: WI2-1347O20 fosmid; *TXLNG*: WI2-1095J6 fosmid; *RBBP7*: WI2-648C17 fosmid. Plasmid, fosmid or BAC probes were prepared using the Nick translation DNA labeling system 2.0 (Enzo) with red, green, or Cy5 dUTPs (red & green: ENZO; Cy5: Cytiva). RNA FISH was performed according to previously published protocol (Bousard et al., 2019). For probe preparation, 4 μL of probe was precipitated using NaAc (Sigma-Aldrich), sheared salmon sperm DNA (Invitrogen), human *COT1* DNA (Invitrogen), and 3 volumes of ethanol (Fisher Chemical). After denaturation (75°C, 7 min) and blocking (37°C, 30 min), probes were co-hybridized in a hybridization buffer overnight at 37°C. hiPSCs and differentiated cells were grown on matrigel-coated coverslips, fixed in 3% PFA, permeabilized with 0.5% Triton X-100, dehydrated in ethanol, and hybridized with fluorescent probes. After washing, nuclei were stained with DAPI and mounted. Imaging was performed using a Zeiss Axio Observer fluorescence microscope (63x oil objective). For RNA FISH analysis of escapees (*TLXNG*, *RBBP7*, and *EIF2S3*), only *HUWE1*/*XACT*-positive cells were counted (Figure S3B). At least 200 cells were analyzed per experiment.

### Bisulfite sequencing

Genomic DNA was extracted using phenol:chloroform:isoamyl alcohol. Bisulfite treatment was performed with the EZ DNA Methylation Gold kit (Zymo Research). Bisulfite-treated DNA was PCR-amplified for YY1 binding sites in *XIST* exon 1 (Figure S1A) using primers in Table S8. PCR products were cloned into pGEM-T Easy (Promega), and at least 10 clones per sample were sequenced. Methylation analysis was done with BiQ Analyzer v.2.02 (Bock et al., 2005).

### Whole Exome Sequencing (WES)

Genomic DNA (1.5 μg) from ASD, F7, F002, and CD was extracted using phenol:chloroform:isoamyl alcohol and sent to NOVOGENE. Libraries were prepared with the Agilent SureSelect Human All Exon V6 kit and paired-end sequenced on Illumina HiSeq 2000. Raw WES data were processed with Trim Galore, aligned to GRCh38 using BWA-MEM, and analyzed with GATK4 (Poplin et al., 2018) for duplicate marking, base recalibration, and variant calling. Variant calling (VCF) file was filtered (GQ > 30, DP > 20, MIN(FMT/AD > 7) and annotated with Ensembl VEP v.96 (McLaren et al., 2016). Chromosome X variants were selected for downstream analysis.

### RNA-seq library preparation and analysis

RNA-seq was performed in triplicates for F7cl15, ASD, F002, F7cl4, CE, and CD hiPSC lines, plus one replicate of ectodermal differentiation (F7cl4/F7cl15 D0 & D7). Total RNA was extracted using NYZol, DNase-treated, and sent to NOVOGENE for quality control (RIN > 9) and sequencing on NovaSeq 6000. Reads were mapped to GRCh38 using STAR (--quantMode GeneCounts) (Dobin et al., 2013), and the number of raw reads mapping to the X chromosome (relative to total reads mapping to autosomes) was used as a proxy for erosion (Figure 2B). DESeq2 (v.1.40.2) (Love et al., 2014) was used to identify differentially expressed genes (DEGs) by establishing a threshold of |log2FC| = 0.33 and an adjusted *p* value < 0.05. Transcripts per Million (TPMs) were used for gene expression quantification and hierarchical clustering.

We divided X-linked genes into five different categories according to their behavior in each comparison: consistently up/down-regulated if the gene was up/down-regulated in three or four cell lines when compared against both ASD and F7cl15 lines, sporadically up/down-regulated if the gene was up/down-regulated only in one or two cell lines when compared against both XIST+ controls, and unchanged if they did not meet any of the previous criteria. Examples of unchanged genes include genes considered up-regulated against F7cl15, but not against ASD, and genes upregulated against ASD, but downregulated against F7cl15. Furthermore, we categorized X-linked genes according to their XCI status as inactive, variable, and escapees following the classification by Werner et al., 2022 as reference and the classification from Tukiainen et al., 2017 for the remaining genes (Tukiainen et al., 2017; Werner et al., 2022).

### Allele-Specific Expression (ASE) Analysis

Haplotype expression data were generated with phASER (v.0.9.9.4; Castel et al., 2016) using RNA-seq-based phasing. Only uniquely mapped reads (base quality ≥10) and loci with ≥10 read depth were analyzed to minimize false positives. Allelic imbalance was assessed using MAF, calculated as the ratio of minor allele read counts (the least common allele) to the total read counts from both alleles. Genes were classified as monoallelic (MAF < 0.10), biallelic (MAF > 0.40), or intermediate (0.10 ≤ MAF ≤ 0.40).

### Re-analyzing epigenomic datasets: ChIP-seq and DNA methylation arrays

We integrated data from Yokobayashi et al. (2021) (GEO: GSE165869) and Bansal et al. (2021) to compare H3K27me3, H3K9me3, and DNA methylation across the X chromosome in eroded vs. non-eroded conditions. ChIP-seq data from female samples F1 (eroded, XIST^−^) and F3 (non-eroded, XIST^+^) showed loss of H3K27me3 and H3K9me3 enrichment in confined regions during erosion. Metagene profiles of histone marks (Figure S2E) were generated using deepTools (v.3.5.1; Ramírez et al., 2014), normalizing signals across genomic regions with a 1 kbp bin size and 5 kbp gene body length.

DNA methylation data from Bansal et al. (2021) (Illumina 450K/850K arrays) was used to assess methylation changes during XCI erosion progression. The authors used probes with higher variance in female vs. male samples (*p* ≤ 0.01) for K-means clustering, identifying six clusters based on methylation levels. The analysis revealed stepwise demethylation associated with Xi erosion. Here, we considered cluster A as the non-eroded (XIST^+^) and cluster C as the eroded (XIST^−^) state. To assess the correlation between gene upregulation and methylation variability, we analyzed promoter methylation across different gene categories. Two-sample t tests showed significant differences between consistently and sporadically upregulated genes (*p* = 0.028) and between sporadically upregulated and unchanged genes (*p* < 0.001) in the eroded state (cluster C).

### Distances to escape genes

We computed the distance between each expressed gene and the nearest upregulated escapee using bedtools closest (Quinlan and Hall, 2010; v.2.30.0). Average distances for consistently upregulated, sporadically upregulated, unchanged, and downregulated genes were plotted. Mann-Whitney tests showed consistently upregulated genes differed significantly from all other categories (*p* < 0.01), while sporadically upregulated and unchanged genes were not (*p* = 0.12).

### 5mC/5hmC measurements by LC-MS

Genomic DNA was extracted using phenol:chloroform:isoamyl alcohol and digested with DNA Degradase Plus (Zymo Research). Nucleosides were analyzed by LC-MS/MS on a Q Exactive mass spectrometer (Thermo Scientific) with a nanoelectrospray ion source. Heavy isotope-labeled nucleosides were added before analysis. MS2 data for 5hmC, 5mC, and C were acquired using a 5 Th isolation window and fragmented by HCD (10% energy, 70,000 resolution). Quantification was done using extracted ion chromatograms and a six-point calibration curve, with triplicate runs for all samples and standards.

### IMPLICON library preparation and analysis

IMPLICON was performed as described (Klobučar et al., 2020) on hiPSCs (F7cl15, F7cl4, CD, CE, 2040cl6, 2042cl1, and 2042cl9) and fibroblasts (2040 Fib and 2042 Fib). After bisulfite conversion, a first PCR amplified target regions per sample, adding adapter sequences and unique molecular identifiers (UMIs) for deduplication (Table S8). Amplicons were pooled, cleaned with AMPure XP beads (Beckman Coulter) and subjected to a second PCR to attach barcoded Illumina adapters. Libraries were quality-checked via Agilent bioanalyzer and sequenced on Illumina MiSeq (paired-end 250 bp) using the indexing primer, 5′-AAGAGCGGTTCAGCAGGAATGCCGAGACCGATCTC-3′, with a 10% PhiX spike-in. Computational analysis followed Klobučar et al. (2020). Illumina pipelines processed data, UMIs were extracted for deduplication, and reads were trimmed (Trim Galore v.0.5.0; (Martin, 2011)). Reads were aligned to the human genome using Bismark v.0.20.0 and deduplicated with UMIs, and CpG methylation calls were extracted. Coverage files were analyzed in Seqmonk v.1.47, with CpGs quantified using the DNA methylation pipeline or total read count method.

### Trilineage specification and cardiac differentiation

hiPSCs (F7cl15, F7cl4, 2041cl13, 2041cl2, 2042cl9, and 2042cl5) were differentiated into ectoderm, mesoderm, and endoderm using the STEMdiff trilineage differentiation kit (STEMCELL Technologies). Ectodermal differentiation was also performed for ASD, F002, and CE hiPSCs. Cells were plated with a density of 200,000 (mesoderm) or 800,000 (endoderm/ectoderm) per well in 12-well plates. Media was changed daily until day 5 (mesoderm/endoderm) or day 7 (ectoderm). After differentiation, cells were collected for RNA extraction (RT-qPCR or RNA-seq) or dissociated for RNA FISH. Primers are listed in Table S8.

Cardiac differentiation of ASD, F002, CD, and CE hiPSCs followed protocol by Lian et al. (2012). hiPSCs were cultured to confluency in mTeSR1 on Matrigel-coated plates before differentiation with RPMI/B-27 (no insulin) and CHIR99021 (GSK3 inhibitor). At day 3, Wnt signaling was inhibited using IWP-4 to promote cardiac fate. Cells matured in RPMI/B-27 until day 30, when cardiomyocytes were collected for RNA extraction, RT-qPCR, and RNA-AMP-seq. Primers are listed in Table S8.

### RNA-AMP-seq library preparation and analysis

Total RNA was isolated, DNase-treated, and reverse-transcribed as described in the RT-qPCR section. RNA-AMP-seq was performed on hiPSCs (ASD, F002, CD, and CE) before and after cardiac differentiation using a protocol similar to IMPLICON. A first PCR amplified target regions, adding adapters and UMIs. Amplicons were pooled, cleaned with AMPure XP beads, and subjected to a second PCR to attach barcoded Illumina adapters. Libraries were validated via Agilent bioanalyzer and sequenced on Illumina MiSeq (paired-end 250 bp) with a 10% PhiX spike-in. Data were processed using Illumina pipelines. UMIs were extracted for deduplication; reads were trimmed (Trim Galore v.0.5.0), aligned to GRCh38 (STAR v.2.7.11a), and deduplicated with UMI-tools. Allelic expression was quantified with phASER v.0.9.9.4, and MAF was calculated as in ASE analysis.

## Resource availability

### Lead contact

Requests for further information and resources should be directed to and will be fulfilled by the lead contact, Simão Teixeira da Rocha (simao.rocha@tecnico.ulisboa.pt).

### Materials availability

This study did not generate new unique reagents.

### Data and code availability

The datasets generated during the current study are available in the Gene Expression Omnibus (GEO): GSE262239. Source code and source data for most analyses in this study are available at GitHub: https://github.com/comicsfct/ErosionX.

## Acknowledgments

We would like to thank the previous and current members of S.T.d.R.’s team for helpful discussions. We also thank Felix Krueger, Maria Gouveia, Teresa Silva, Marta Furtado, and Adriana Vieira for technical help during the execution of this work. Work in S.T.d.R.’s team was supported by 10.13039/501100001871Fundação para a Ciência e a Tecnologia (FCT) 10.13039/501100006111Ministério da Ciência, Tecnologia e Ensino Superior (MCTES), Portugal (IC&DT projects PTDC/BIA-MOL/29320/2017 and 2022.01532.PTDC as well as projects UIDB/04565/2020 and UIDP/04565/2020 of the Research Unit Institute from Bioengineering and Biosciences – iBB and LA/P/0140/2020 of the Associate Laboratory Institute for Health and Bioeconomy – i4HB). A.C.R., M.A., and P.B. are supported, respectively, by SFRH/BD/137099/2018, SFRH/BD/151251/2021, and SFRH/BD/137062/2018 PhD fellowships from FCT/10.13039/501100006111MCTES. P.C. was a recipient of a Marie Skłodowska-Curie Postdoctoral Fellowship (FOX-MTN-HORIZON-MSCA - 2021-PF-01-01) and an FCT Scientific Employment Stimulus Contract (2023.06750.CEECIND). S.T.d.R. was supported by an assistant research contract from FCT/10.13039/501100006111MCTES (2021.00660.CEECIND/CP1651/CT0018).

## Author contributions

A.C.R. performed the characterization of the majority of all hiPSCs and molecular biology experiments (RNA-seq, WES, RNA-FISH, and cardiac differentiation). P.C. conducted all bioinformatics analyses (RNA-seq, DGE, ASE, and all data visualizations). J.J. prepared and analyzed all the trilineage experiments (RNA-seq, RNA-FISH FISH, and Sanger sequencing) and RNA FISH experiments for escapees with the close assistance of F.C.M. J.J. also assisted in 5mC/5hmC measurements. M.A. prepared and analyzed the IMPLICON and RNA-AMP-seq experiments. P.B. mapped and analyzed the WES data, and R.S.-L. performed initial RNA-seq analyses. D.O. performed 5mC and 5hmC experiments. F.A. analyzed the RNA-AMP-seq results. A.M. assisted in the 5mC/5hmC measurements and IMPLICON. M.E.-M. supervised the analysis of the IMPLICON data. M.C. provided consulting on the experiments and critically revised the manuscript. A.R.G. supervised the bioinformatics analyses. S.T.d.R. conceived and supervised the study and secured funding. S.T.d.R., P.C., and A.C.R. interpreted the data and wrote the manuscript. All the authors have read and agreed to the published version of the manuscript.

## Declaration of interests

A.M. is an Altos Labs employee.

## Declaration of generative AI and AI-assisted technologies in the writing process

During the preparation of this work, the author(s) used ChatGPT in order to improve language and readability, with caution. After using this tool/service, the authors reviewed and edited the content as needed and take full responsibility for the content of the publication.

## Supplemental information

Data S1. Tables S1–S8

Document S2. Article plus supplemental information
