# Supplementary material for: Gene reactivation upon erosion of X chromosome inactivation in female hiPSCs is predictable yet variable and persists through differentiation
Source: Stem Cell Reports. 2025 Apr 3;20(5):102472. doi: 10.1016/j.stemcr.2025.102472 (PMC12143139; doi:10.1016/j.stemcr.2025.102472)
Supplement: Document S1. Figures S1–S5 [file mmc1.pdf]

Stem Cell Reports, Volume *20*

Supplemental Information

Gene reactivation upon erosion of X chromosome inactivation in female hiPSCs is predictable yet variable and persists through differentiation

Ana Cláudia Raposo, Paulo Caldas, Joana Jeremias, Maria Arez, Francisca Cazaux Mateus, Pedro Barbosa, Rui Sousa-Luís, Frederico Água, David Oxley, Annalisa Mupo, Melanie Eckersley-Maslin, Miguel Casanova, Ana Rita Grosso, and Simão Teixeira da Rocha

SUPPLEMENTARY FIGURE 1

A

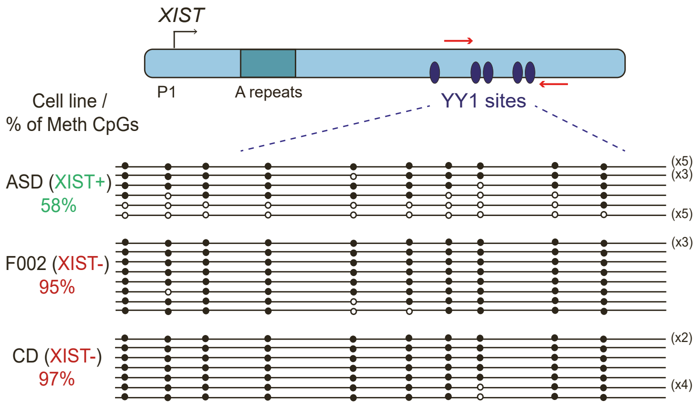

B

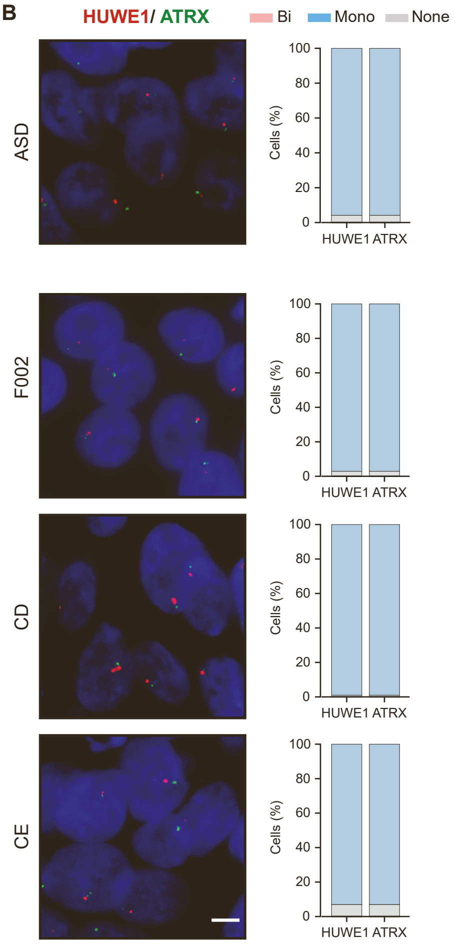

C

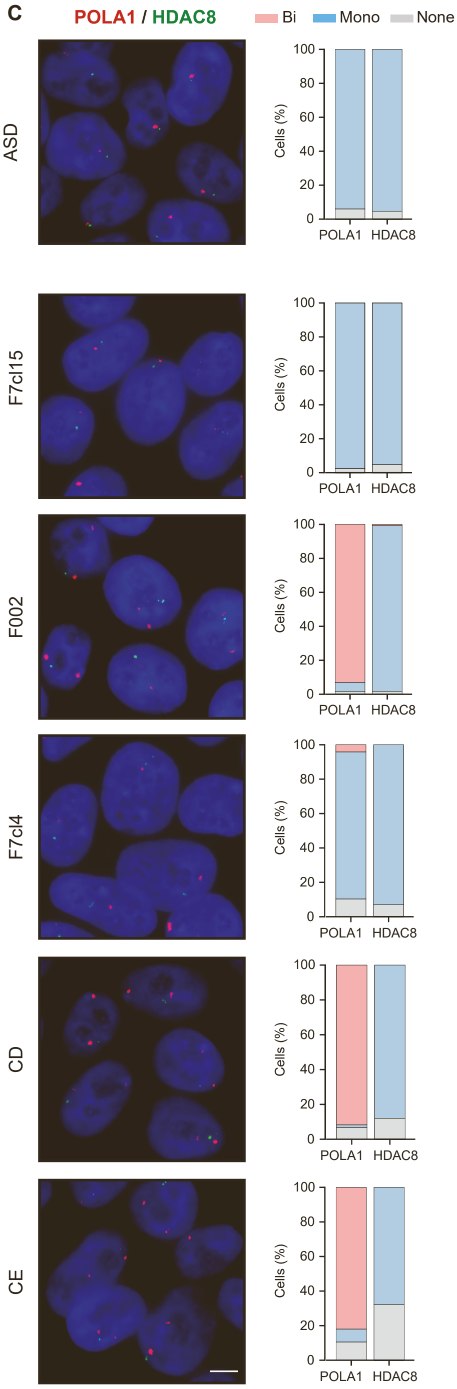

D

Cell lines	XCI status	DNA<br>*ATRX*<br>(rs3088074: C/G)
F7cl15	XIST+	C/G
ASD	XIST+	C/G
F002	XIST-	C/C
F7cl4	XIST-	C/G
CE	XIST-	C/G
CD	XIST-	C/G

E

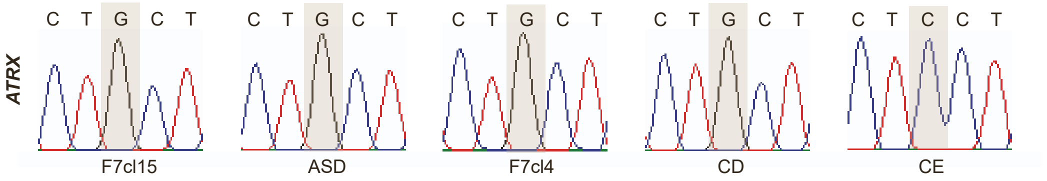

SUPPLEMENTARY FIGURE 2

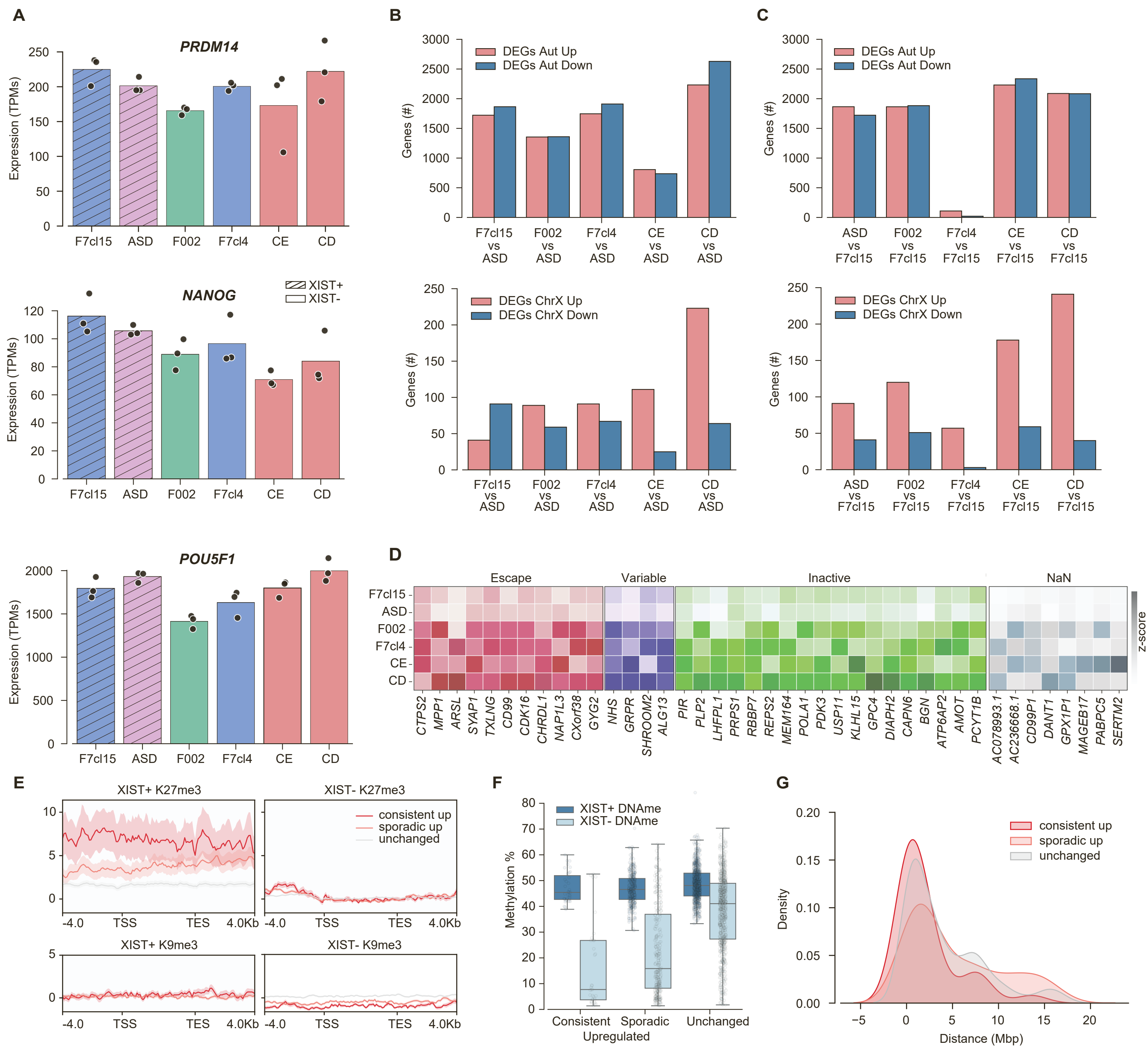

SUPPLEMENTARY FIGURE 3

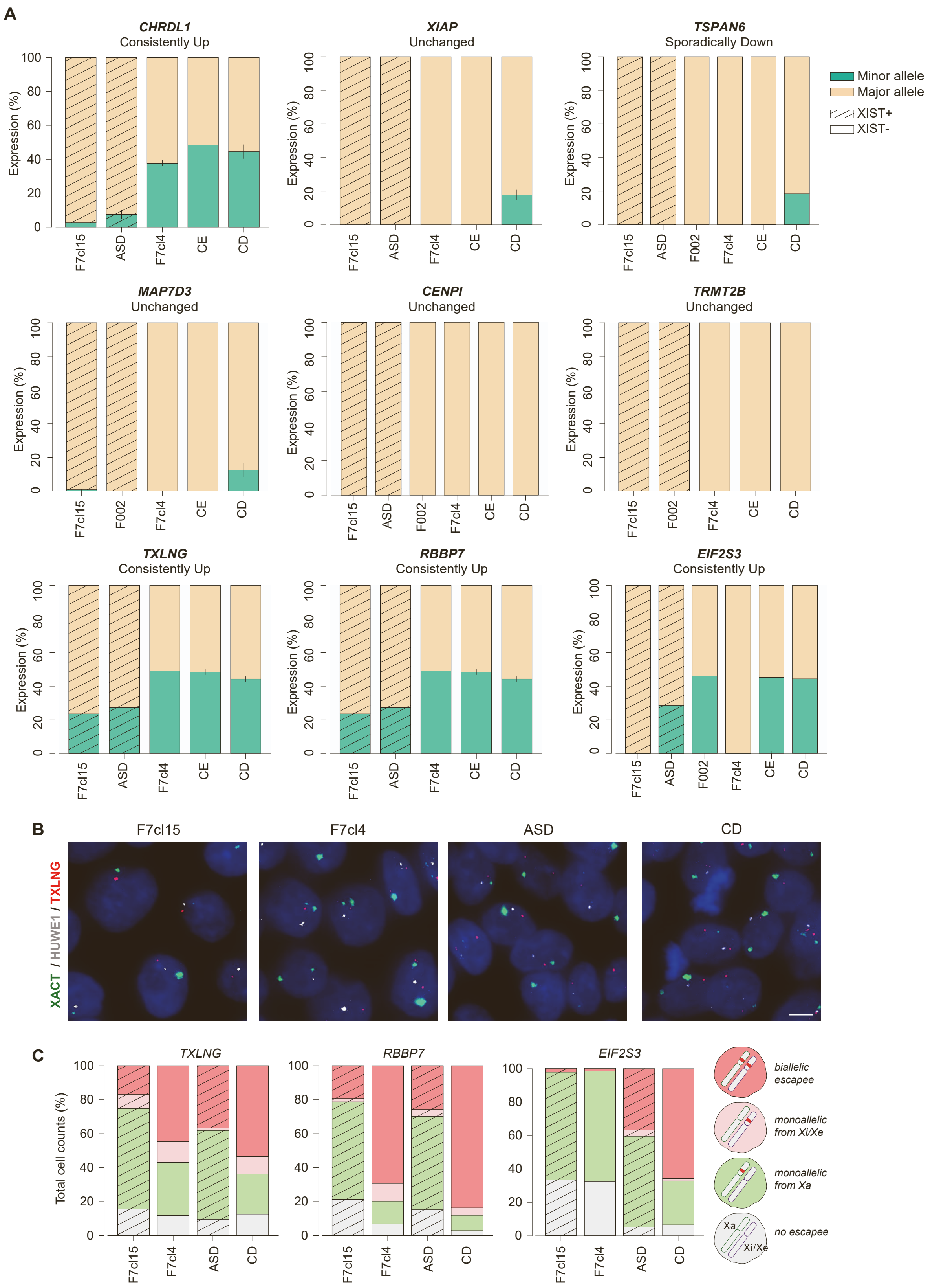

SUPPLEMENTARY FIGURE 4

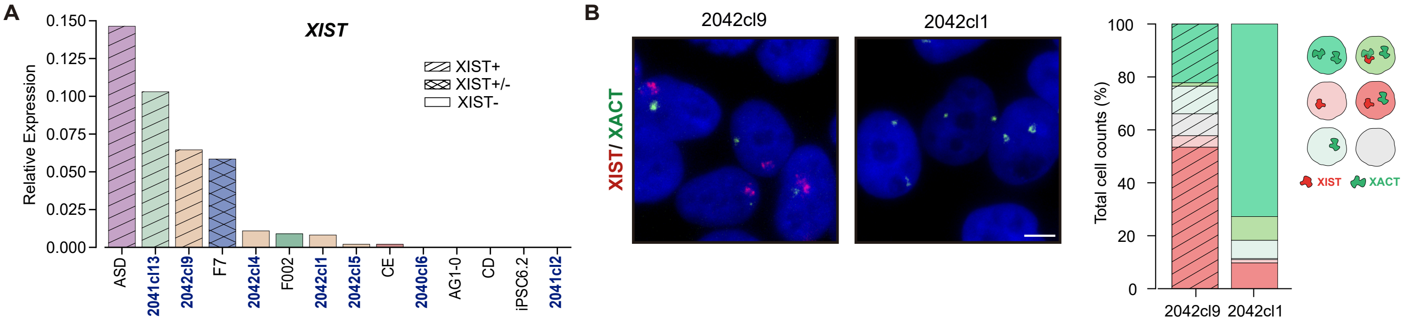

SUPPLEMENTARY FIGURE 5

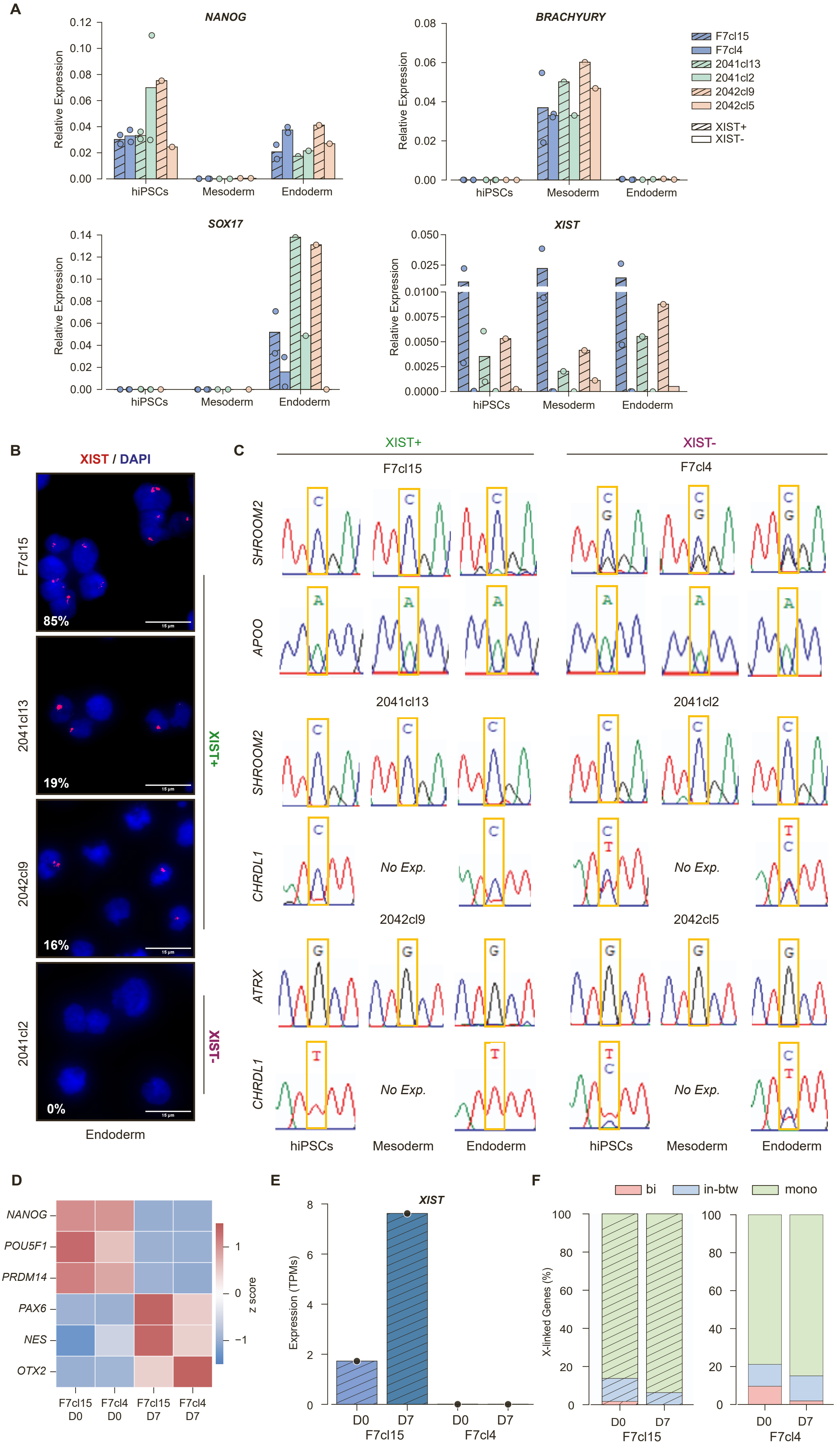

LIST OF SUPPLEMENTARY FIGURES

Fig. S1: Characterization of the XCI status in female hiPSCs

A. Schematic representation of YY1 binding sites within the *XIST* locus relative to A repeats motif. Bisulfite sequencing analysis within a region on *XIST* exon 1 containing YY1 binding sites in ASD, F002 and CD iPSCs. Each line represents the methylation profile of an independent PCR amplicon analyzed by Sanger sequencing and BiQ Analyser. White dots: unmethylated CpGs; black dots: methylated CpGs; % of methylated (Meth) CpGs was calculated as follows: average percentage of the number of methylated CpGs (black dots) / total number of CpGs (black + white dots) per cloned PCR product; the numbers in brackets represent the number of times the same amplicon was sequenced; the repetitive amplicons were only counted as one to determine the % of methylation. **B-C.** Representative RNA FISH images for *HUWE1* (red) and *ATRX* (green) in ASD, F002, CD and CE human hiPSCs (B) and for *POLA1* (red) and *HDAC8* (green) in isogenic F7cl15, ASD, F002, F7cl4, CD and CE iPSCs (C). DNA stained in blue by DAPI; scale bar: 5 µm; graphs represent % of cells with monoallelic (mono), biallelic (bi) or no expression (none). The values represent 1–2 independent experiments, where a minimum of 200 cells were counted per experiment. **D.** Summary table showing presence of heterozygosity at the SNP rs3088073 in the *ATRX* gene in F7cl15, ASD, F002, F7cl4, CE and CD human iPSCs. Bold letters refer to the presence of heterozygosity. **E.** Allelic expression of *ATRX* gene assayed by RT-PCR followed by Sanger sequencing. Chromatograms are shown for each cell line with the respective SNP highlighted in gray.

Fig. S2: Features of X-linked genes overexpressed in eroded hiPSCs

A. Expression analysis by RNAseq of *PRMD14*, *NANOG* and *POU5F1* pluripotent genes in F7cl15, ASD, F002, F7cl4, CE and CD hiPSCs. The graph shows the Transcripts per Million (TPMs) expression values from biological triplicates (black dots) of each sample. **B-C.** Barplots showing the number of upregulated (red) and downregulated (blue) Differentially Expressed Genes (DEGs) in autosomes (Aut) and X chromosome (ChrX) in F7cl15, F002, F7cl4, CE and CD hiPSCs compared with ASD XIST+ line (B) and with F7cl15 XIST+ line (C). **D.** Heatmap illustrating the expression levels of consistently upregulated genes categorized by their inactive/escape status (Escape, Inactive, Variable, and NaN) for multiple hiPSC lines (F7cl15, ASD, F002, F7cl4, CE, and CD). Each category has its genes color-coded to reflect expression levels, with darker shades representing higher expression levels. **E.** Metagene profiles illustrate the distribution of H3K27me3 and H3K9me3 signals in consistently upregulated, sporadically upregulated, and unchanged gene categories based on ChIP-seq data of XIST+ and XIST- hiPSCs from Yokobayashi et al., 2021 (see Materials and Methods for details). A two-sample t-test revealed significant differences for upregulated versus sporadically

upregulated genes and consistent upregulated versus unchanged genes (*p* << 0.01; Cohen's d > 1) for H3K27me3 in XIST+ cell lines. **F.** Boxplot showing the methylation percentage of promoter regions in consistently upregulated, sporadically upregulated, and unchanged genes in XIST+ and XIST- hiPSC lines from Bansal et al. 2021 (see Materials and Methods for details). Statistical comparisons were made using two-sample t-tests (*p* = 0.028 for consistently vs sporadic, *p* << 0.01 for sporadic vs unchanged) in cluster 3 (eroded state). **G.** Density plot showing the distribution of distances (in mega bases - Mbp) between each gene category (consistent up, sporadic up and unchanged) and their nearest upregulated constitutive escapee.

Fig. S3: RNAseq allele-specific expression analysis in female hiPSC lines

A. Allele-specific expression (ASE) based on RNAseq analysis from representative genes containing common SNPs across different hiPSCs. Bar plots displaying the average percentage of expression ± SEM of the major and minor alleles for the X-linked genes *SHROOM2*, *XIAP, TSPAN6*, *MEP7D3*, *CENPI*, *TRMT2B*, *TXLNG*, *RBPP7*, and *EIF2S3* in XIST+ (F7cl15, ASD) and XIST- (F002, F7cl4, CE and CD) hiPSCs. **B.** Representative RNA FISH images for *TXLNG* (red; escapee), XACT (green) and HUWE1 (white) in F7cl15 (XIST+), F7cl4 (XIST-), ASD (XIST+) and CD (XIST-) cell lines. DNA stained in blue by DAPI; scale bar: 5 µm; **C.** Quantification of single-cell expression levels for three escape genes (*TXLNG*, *RBBP7*, and *EIF2S3*) across these hiPSC lines. Bars indicate the proportion of cells expressing the escape gene from the active X chromosome (Xa, green), the inactive/eroded X chromosome (Xi/Xe, salmon), or both chromosomes (XaXi, red). Cells with no detectable expression are shown in gray. A minimum of 200 cells counted per experiment. Scale bar: 5 µm.

Fig S4: Analysis of XCI in the seven newly generated hiPSC lines during this study

A. Barplot showing RT-qPCR analysis of *XIST* expression normalized to *GAPDH* housekeeping gene in ASD, 2041cl13, 2042cl9, F7, 2042cl4, F002, 2042cl1, 2042cl5, CE, 2040cl6, AG1-0, CD, iPSC6.2 and 2041cl2 female hiPSCs. Bars represent the average *XIST/GAPDH* expression. n=1 for all iPSCs. **B.** Representative RNA FISH images for *XIST* (red) and *XACT* (green) in 2042cl9 and 2042cl1 hiPSCs; DNA stained in blue by DAPI; scale bar: 5 µm; graph represents % of cells with different expression profiles for XIST and XACT by RNA FISH as depicted in the legend on the left; a minimum of 200 cells were counted. Scale bar: 5 µm.

Fig S5: Erosion pattern persists upon mesodermal and endodermal commitment.

A. Barplots showing mean relative gene expression of *NANOG* (pluripotency marker), *BRACHYURY* (mesodermal marker), *SOX17* (endodermal marker) and *XIST* (Xi marker),

quantified by RT-qPCR upon normalization for *GAPDH* housekeeping gene in F7cl15, F7cl4, 2041cl13, 2041cl2, 2042cl9 and 2042cl5 hiPSCs, mesodermal and endodermal cells; n=2 for all samples, except for undifferentiated 2042 clones and differentiated (Endoderm and Mesoderm) 2041 and 2042 clones (n=1); Note that the barplots of *NANOG* and *XIST* for iPSCs are the same represented in Fig. 5B. **B.** Representative images of XIST RNA-FISH and respective percentages of cells expressing XIST (red dots) in F7cl15, 2041cl13, 2042cl9 and 2041cl2 at differentiated endodermal cells. The nuclei are counterstained with DAPI (blue). Scale bars represent 15 μm. Number of cells counted: F7cl15: 327, 2041cl13: 266, 2042cl9: 243, 2041cl2: 164. The values represent 1 independent experiment. **C.** Allelic expression assayed by RT-PCR followed by Sanger sequencing resourcing to informative SNPs to distinguish the two alleles. The chromatograms represent illustrative examples of the allelic expression of heterozygous X-linked genes for each XIST+/XIST- isogenic hiPSC pair in hiPSCs and after mesoderm and endoderm specification: *SHROOM2* and *APOO* gene for the F7cl15 and F7cl4, *SHROOM2* and *CHRDL1* gene for 2041cl13 and 2041cl2, and *ATRX* and *CHRDL1* gene for the 2042cl9 and 2042cl5. Note that chromatograms for iPSCs are the same as illustrated in Fig. 5D; **D.** Heatmap showing the RNAseq expression levels for pluripotent markers (*NANOG*, *POU5F1* and *PRDM14*) and ectoderm-specific markers (*PAX6*, *NES* and *OTX2* in F7cl15 (XIST+) and in F7cl4 (XIST-) cells before (D0) and after (D7) ectodermal differentiation. **E.** *XIST* expression data by RNAseq in F7cl15 and F7cl4 before (D0) and after (D7) ectodermal differentiation (D7) (n=1). The graph shows the Transcripts per Million (TPMs) expression value. **F.** Percentage of genes classified as biallelic, intermediate, or monoallelic in F7cl15 (XIST+) and F7cl4 (XIST-) cells before (D0) and after (D7) ectodermal differentiation. Classes were defined based on the minor allele frequency (Methods for details).

LIST OF SUPPLEMENTARY TABLES

Table S1: Description of original Cell Lines used in this study

Table S2: Differential Gene Expression Analysis (DGEA) of X-linked Genes in all cell lines

Table S3: Allelic Specific Expression (ASE) of X-linked Genes in all cell lines

Table S4: Description of Cell Lines generated by reprogramming fibroblasts

Table S5: Methylation analysis of 7 imprinted locus

Table S6: Informative SNPs found for the different isogenic pairs

Table S7: Allelic Specific Expression (ASE) of X-linked Genes in Cardiac Cells

Table S8: Primers and Conditions

EXPANDED MATERIAL AND METHODS

Ethics

hiPSC lines used in this study were either purchased or previously generated by us (Pólvora-Brandão et al., 2018; Silva et al., 2021b, 2021a) (Table S1; Table S4). Written informed consents were obtained by the donor or their legal guardian and ethically approved by the Ethics committee of the Lisbon Academic Medical Center, Lisbon, Portugal (approval numbers: 535/12 and 170/18).

Stem Cell Culture

All the hiPSC lines used in this study were cultured with mTeSR™ Plus medium (#05825, Stem Cell Technologies) in 6-well plates previously coated with Matrigel™ (#354230, Corning). Medium was changed every 24/48 hours (hrs) and the cells were routinely passed using 0.5 mM EDTA (#15575020, Invitrogen) in 1x Phosphate-Buffered Saline (PBS; #21600-044, Gibco). Cells were grown at 37ºC and kept in a humidified 5% CO_2 incubator in normoxia conditions.

To freeze, hiPSCs were dissociated with 0.5 mM EDTA/1x PBS and collected with Washing medium (Dulbecco's Modified Eagle Medium/Nutrient Mixture F-12 - DMEM-F12 #11320-033, Gibco, 10% KnockOut™ Serum Replacement - KSR, #10828-028, Gibco, 1% MEM Non-essential Amino Acid Solution 100x - NEAA, #11140-050, Gibco), 1 mM L-Glutamine (#25030081, Thermo Fisher Scientific), 0.1 mM β-mercaptoethanol, (#31350-010, Gibco). After 3 minutes at 1000 rotations per minute (rpm) of centrifugation, the cells pellet was resuspended with a freezing medium composed of 90% KSR and 10% of Dimethyl Sulfoxide (DMSO, #D2438, Merck). Cell vials were stored in a liquid nitrogen tank.

iPSC cultures were regularly scanned for mycoplasma contamination using the qPCR Mycoplasma Test (Mycoplasmacheck, Eurofins Genomics), following the manufacturer’s instructions.

RT-qPCR

Total RNA was isolated from all hiPSCs lines using NZYol™ RNA Isolation Reagent (#MB18501, NZYTech) and then treated with DNaseI (#04716728001, Roche) to remove contaminating DNA and according to manufacturer’s instructions. DNaseI-treated RNA (500 ng) was reverse-transcribed using random primers and a High-Capacity cDNA Reverse Transcription Kit (#4368814, Applied Biosystems) according to the manufacturer’s instructions. Reverse Transcriptase quantitative PCR (RT-qPCR) was performed using NZYSpeedy qPCR Green Master Mix ROX (#MB22302, NZYTech) or NZYSpeedy qPCR Green Master Mix ROX Plus (#MB22202, NZYTech) in StepOne™ or ViiA™ 7 Real-Time PCR

Systems (Applied Biosystems). All PCR reactions were done with technical duplicates or triplicates and then normalized to the *GAPDH* housekeeping gene. The primers used are listed in Table S8. The results were analyzed with the StepOne™ or the QuantStudio™ RT-PCR softwares. The relative expression of each gene was determined using the $2^{-\Delta\Delta CT}$ method.

PCR/RT-PCR followed by Sanger Sequencing

To verify the presence of a specific SNP in ASD, F7, F002, CD, CE, 2041cl13, 2041cl2 and 2042cl9, 2042cl5 hiPSCs, genomic DNA isolated using conventional phenol:chloroform:isoamyl alcohol (#15593-031, Invitrogen) extraction was amplified using primers in the PCR section of Table S8. To analyze relative allelic expression of X-linked genes in F7cl15, F7cl4, 2041cl13, 2041cl2, 2042cl9, 2042cl5, ASD, F002, CD and CE hiPSCs and their ectodermal, mesodermal or endodermal derivatives, cDNA synthesized as described in RT-qPCR section was amplified by PCR using the primers in Table S8. Both PCR products (DNA or cDNA) were cleaned using the NZYGelpure kit (#MB01102, NzyTech) and sent for Sanger sequencing to STABVIDA with data visualized and analyzed using Chromas v2.6.2 software.

RNA FISH

The templates used for probe production were the following: XIST, a plasmid containing the 10Kb exon 5-6 plasmid (Rosspopoff et al., 2023); *XACT*: RP11-35D3 Bacterial Artificial Chromosome (BAC); *ATRX*: RP11-42M11 BAC; *HUWE1*: RP11-155O24 BAC; *HDAC8*: RP11-1021B19 BAC; *POLA1*: RP11-1104L9 BAC; *EIF2S3*: WI2-1347O20 fosmid; *TXLNG*: WI2-1095J6 fosmid; *RBBP7*: WI2-648C17 fosmid. Plasmid, fosmid or BAC probes were prepared using the Nick translation DNA labeling system 2.0 (#ENZ-GEN111-0050, Enzo) with red, green or Cy5 dUTPs (red: #ENZ-42844L-0050, green: #ENZ-42831L-0050, Enzo; Cy5: PA55022, Cytiva). RNA FISH was performed according to previously published protocol (Bousard et al., 2019). For probe preparation, 4 µl of probe was precipitated using 1/10 3M NaAc (#S2889, Sigma-Aldrich), sheared salmon sperm DNA (#AM9680, Invitrogen), human *COT1* DNA (#15279011, Invitrogen) and 3 volumes of ethanol (#10000652, Fisher Chemical). The pellet was resuspended in 6 µl of deionized formamide (#F9037, Sigma) and dissolved for 15 min at 37ºC with agitation. Then, the probes were denatured at 75ºC for 7 min and incubated at 37ºC for 30 min to prevent non-specific hybridization by *COT1* DNA. The probes were co-hybridized in hybridization buffer (20% dextran sulfate (#42867-5G, Sigma), 2x saline-sodium citrate (SSC; #S6639, Sigma-Aldrich), 1 µg/µl BSA (#R396A, Promega), 10 mM vanadyl-ribonucleoside (VRC; #S1402S, New England Biolabs) overnight at 37ºC. For the experiments on Fig.1B-C, Fig.S1B-C and Fig. S4B), hiPSCs were grown on matrigel-coated coverslips, while hiPSCs and ectodermal and endodermal differentiated cells in Fig. 5C and

Fig. S5B were dissociated with Accutase^{TM} (#A6964, Merck) and incubated onto poly-L-lysine (#P4832, Sigma)-coated 22x22 mm coverslips (#0101050, Marienfeld) for 5 min before RNA FISH procedure. Then, cells were fixed in 3% paraformaldehyde (PFA; #043368.9M, Thermo Fisher Scientific) for 10 min at room temperature (RT) and permeabilized for 5 min in 0.5% Triton X-100 (#T9284, Sigma) with 2 mM VRC diluted in PBS on ice. Cells were then dehydrated through 3 min incubations in 70%, 80%, 95% and 100% ethanol solutions and air-dried for 10 min before hybridization with probes. Coverslips were hybridized with fluorescent-labeled probes overnight at 37ºC in a humid chamber with FA/SSC solution (50% deionized formamide, 2x SSC). On the next day, washes were carried out using FA/SSC solution, three times for 7 min at 42ºC, and then with 2x SSC, three times for 5 min at 42ºC. Nuclei were stained with 1:10.000 dilution of DAPI 0.2 mg/mL (#D9542, Sigma-Aldrich) in 2x SSC for 3 min at RT. Coverslips were then mounted on slides with mounting media. Cells were observed with the widefield fluorescence microscope Zeiss Axio Observer (Carl Zeiss MicroImaging) using a 63x oil objective and filter sets FS43HE, FS38HE, FS49 and FS50. More than 200 cells were counted per experiment. For the RNA FISH analysis of escapees (*TLXNG, RBBP7 and EIS2F3*) in Fig. S3B, only cells positive for *HUWE1* and *XACT* RNA FISH signals were considered for counting.

Bisulfite sequencing

Genomic DNA was purified using conventional phenol:chloroform:isoamyl alcohol extraction. Bisulfite treatment was performed using the EZ DNA Methylation Gold Kit (#D5006, Zymo Research) following manufacturer's guidelines. Bisulfite-treated DNA was amplified by PCR for the YY1 binding sites within exon 1 of *XIST* (Fig. S1A) using the primers and conditions summarized in Table S8. PCR products were cloned into the pGEM-T Easy vector (#A1360, Promega) and at least 10 clones from each sample were sequenced. Methylation analysis was performed using BiQ Analyser v2.02 (Bock et al., 2005).

Whole Exome-sequencing (WES)

Genomic DNA from ASD, F7, F002 and CD were purified using conventional phenol:chloroform:isoamyl alcohol extraction. Genomic DNA (1.5µg) was sent to NOVOGENE that conducted whole-exome sequencing (WES). Briefly, genomic DNA was fragmented into 180–280 bp by sonication and subjected to library preparation using the Agilent SureSelect Human All Exon V6 Kit (#5190-8864, Agilent Technologies). The enriched libraries underwent paired-end 150bp sequencing on the Illumina HiSeq 2000 platform.

Raw WES data from hiPCS cell lines was preprocessed with TrimGalore v0.4.4 (Martin, 2011) to remove possible sequencing adapters and filter sequences by Phred quality scores ("-q 20 –length 75 --stringency 5 --trim-n --max_n 2). Reads were further aligned with bwa mem

(v0.7.15-r1140) (Li, 2013) against the GRCh38 genome assembly and duplicates were flagged with GATK4 MarkDuplicatesSpark (McKenna et al., 2010). Base scores were recalibrated (GATK4 Base Quality Score Recalibration), and a germline joint calling approach was performed with GATK4 HaplotypeCaller (Poplin et al., 2018) and GenotypeGVCFs to generate raw genotype calls. Variant calling was restricted to regions covered by Agilent v6 kit. Raw variant filtering was done with GATK4 VariantFiltration by employing hard filters based on several annotations (default values following GATK recommendations). All GATK4-based analyses were executed using the GATK version 4.1.2.0. Further processing of the call set was performed with bcftools v1.9 (Li, 2011) (multiallelic sites split, indel normalization and quality filtering), where thresholds of GQ > 30, DP > 20 reads and a minimum of 7 reads of the least covered allele were required (MIN(FMT/AD > 7). Filtered variants were annotated with Ensembl VEP (McLaren et al., 2016) using the 96 release. Chromosome X variants were further selected for downstream analysis.

RNA-sequencing (RNA-seq) library preparation and analysis

Triplicates of F7cl15, ASD, F002, F7cl4, CE and CD hiPSC lines as well as one replicate of an ectodermal differentiation series for the F7cl15 and F7cl4 hiPSC pair (F7cl4 D0 and D7 & F7cl15 D0 and D7) were used for RNA-seq. Total RNA was isolated using NYZol and then DNase I-treatment was performed to remove contaminating DNA following the manufacturer's recommendations. RNA (1 µg) was sent to NOVOGENE where quality of the samples was verified on a 2100 Agilent Bioanalyser system. Only samples with RIN score above 9 were processed. RNA was used for 250–300 bp insert cDNA library following manufacturer's recommendations and libraries were sequenced with NovaSeq 6000 platform using paired-end 150-bp mode.

To quantify gene expression from our bulk RNAseq data (paired-ended strand-specific), we mapped the reads to the reference genome (GRCh38 assembly; release 37, GRCh38.p13) using STAR (v2.7.8a) (Dobin et al., 2013) using the *--quantMode GeneCounts* option.

The number of raw reads mapping to the X chromosome (relative to the total number of reads) was used as a proxy for erosion (Fig 2B). We calculated Transcripts per Million (TPMs) for all genes in all samples directly from the read count matrix using a custom python script.

$$TPM = A\,\frac{1}{\sum A} \times 10^6$$

$$\text{where } A = \frac{Total\ read\ mapped\ to\ gene \times 10^3}{gene\ length\ in\ bp}$$

This TPM matrix was then used to show *XIST* expression levels across samples and perform hierarchical clustering.

Differential gene expression was assessed using the DESeq2 (v 1.40.2) R package (Love et al., 2014). We compared each XIST- hiPSC against each XIST+ hiPSC line and filtered differentially expressed genes (DEGs) by establishing a threshold of |log2FC| = 0.33 and an adjusted p-value < 0.05. This threshold was chosen to encompass most genes that reactivate from the Xi regardless of whether they are inactive, variably expressed, or inactive genes, with an expected increase in bulk expression ranging from 1.25 to 2 times. We divided X-linked genes into five different categories according to their behavior in each comparison: consistently upregulated/downregulated (if the gene was upregulated/downregulated in three or four cell lines when compared against both ASD and F7cl15 lines); sporadically upregulated/downregulated (if the gene was upregulated/downregulated only in one or two cell lines when compared against both XIST+ controls) or unchanged (if they did not meet any of the previous criteria: e.g. gene was considered upregulated against F7cl15, but not against ASD; gene was upregulated against ASD, but downregulated against F7cl15).

Furthermore, we categorized X-linked genes according to their XCI status as inactive, variable and escapees following the classification by Tukiainen et al 2017 and then reviewed by Werner et al., 2022 (Tukiainen et al., 2017; Werner et al., 2022). We consider the classes reviewed by Werner *et al.* as reference and the classification from Tukiainen *et al*. 2017 for the remaining genes not classified by Werner *et al*., 2022.

Allele-Specific Expression (ASE) Analysis

After conducting WES and storing gene sequence variations in a Variant Call Format (VCF), we used phASER (v.0.9.9.4) (Castel et al., 2016) for RNAseq-based phasing, enabling the generation of gene-level haplotype expression data. Only reads uniquely mapped and with a base quality ≥10 were used for phasing. For our downstream analyses, we further discarded loci with a total read depth lower than 10. This limits the number of genes in our analysis but reduces the number of false positives due to biased RNA-seq read mapping or other technical artifacts. The effect size of allelic imbalance in expression for each gene in each sample was determined using the Minor Allele Frequency (MAF), calculated as the ratio of minor allele read counts (the least common allele) to the total read counts from both alleles. We defined genes with a MAF < 0.10 as fully monoallelic, genes with a MAF > 0.40 as fully biallelic, and the remaining genes as “intermediate”.

Re-analyzing Epigenomic datasets: ChIP-seq and DNA methylation arrays

We integrated data from two studies to visualize the positioning of H3K27me3, H3K9me3, and DNA methylation levels across the X chromosome, comparing eroded and non-eroded conditions. From Yokobayashi et al. (Yokobayashi et al., 2021) (GEO: GSE165869), we used ChIP-seq peaks (bigwig files) from two female samples (F1 and F3) showing the distribution

of H3K27me3 and H3K9me3 marks. F3 (XIST+, non-eroded) shows H3K27me3 and H3K9me3 distributed along the X chromosomes in a largely mutually exclusive manner, while F1 (XIST-, eroded) shows loss of H3K27me3 and enrichment of H3K9me3, but to confined/narrow regions.

To corroborate the enrichment of each histone mark in specific gene categories ('consistently' and 'sporadically' upregulated and unchanged), we generated metagene profiles to visualize the distribution of histone mark signals across key genomic regions. Using the *computeMatrix* tool from *deepTools* ((Ramírez et al., 2014); v3.5.1), we calculated normalized scores across genomic regions, enabling unbiased comparisons across genes. The scale-regions mode was used to standardize regions of interest by stretching or shrinking them to a uniform length. We set a bin size of 1 kbp and a body length of 5 kbp, ensuring consistent visualization of scores across regions of varying lengths. The resulting profiles were visualized using *deepTools plotProfile*.

DNA methylation data from Bansal et al. (2021) obtained from Illumina 450K and 850K Methylation Array data for primed human iPSC and ESC samples was used to examine variations in methylation levels throughout the progression of XCI erosion (Bansal et al., 2021). Probes with higher variance in female samples compared to male samples ($p \leq 0.01$) were selected for K-means clustering of female samples. This approach identified six distinct clusters, ordered by their average methylation levels, and revealed all differentially methylated probes (DMPs) between neighboring clusters. The analysis demonstrated a clear, systematic pattern of stepwise de-methylation changes, highlighting the progressive erosion of the Xi. We designated cluster A as the non-eroded ($XIST^+$) state and cluster C as the eroded ($XIST^-$) state, as the cluster C contained the biggest number of eroded hiPSCs. Clusters D, E and F, while more extensively eroded, cover a smaller number of hiPSCs and likely represent rarer states of erosion not phenocopied by our hiPSC lines.

To quantify the correlation between the location of upregulated genes and regions with highly variable changes in methylation levels, we compared the percentage of methylation in regions where upregulated genes were located across different categories (consistent up, sporadic up and unchanged). Specifically, we focused on methylation levels in the promoter regions of these genes. Statistical comparisons between groups within each cluster (cluster A and cluster C) were conducted using two-sample t-tests. The resulting p-values were assessed for statistical significance using a threshold of $p < 0.05$. Consistently upregulated genes are significantly different from sporadically upregulated genes ($p = 0.028$), and sporadically upregulated genes are significantly different from unchanged genes ($p < 0.001$) in cluster three (eroded state).

Distances to escapee genes

A comprehensive list of all genes was compiled, along with a second list containing only constitutively upregulated escapees. Using *bedtools closest* (Quinlan and Hall, 2010); v.2.30.0), the closest escapee - whether upstream or downstream and regardless of strand orientation - was identified for each gene in the first list, and the distance between them was recorded. The average distances were then calculated for consistently upregulated, sporadically upregulated, unchanged, and downregulated genes, and the results were visualized in a plot. Statistical analysis using the Mann-Whitney test revealed that consistently upregulated genes were significantly different from all other categories ($p<0.01$), while sporadically upregulated and unchanged genes were not significantly different from each other ($p=0.12$).

5mC/5hmC measurements by Liquid Chromatography-Mass Spectrometry (LC-MS)

Genomic DNA from hiPSCs was purified using conventional phenol:chloroform:isoamyl alcohol extraction and digested using DNA Degradase Plus (#E2020, Zymo Research) according to the manufacturer's instructions. Nucleosides were analyzed by LC-MS/MS on a Q-Exactive mass spectrometer (Thermo Scientific) fitted with a nanoelectrospray ion-source (Proxeon). All samples and standards had a heavy isotope-labeled nucleoside mix added prior to mass spectral analysis (2′-deoxycytidine-$^{13}C_1$, $^{15}N_2$ (#SC-214045, Santa Cruz), 5-(methyl-$^2H_3$)-2′-deoxycytidine (#SC-217100, Santa Cruz), 5-(hydroxymethyl)-2′-deoxycytidine-$^2H_3$ (#H946632, Toronto Research Chemicals). MS2 data for 5hmC, 5mC and C were acquired with both the endogenous and corresponding heavy-labeled nucleoside parent ions simultaneously selected for fragmentation using a 5 Th isolation window with a 1.5 Th offset. Parent ions were fragmented by Higher-energy Collisional Dissociation (HCD) with a relative collision energy of 10%, and a resolution setting of 70,000 for MS2 spectra. Peak areas from extracted ion chromatograms of the relevant fragment ions, relative to their corresponding heavy isotope-labeled internal standards, were quantified against a six-point serial 2-fold dilution calibration curve, with triplicate runs for all samples and standards.

IMPLICON Library Preparation and Analysis

IMPLICON was performed as previously described (Klobučar et al., 2020) in F7cl15, F7cl4, CD, CE, 2040cl6, 2042cl1 and 2042cl9 hiPSCs, and, 2040 and 2042 fibroblasts (2040 Fib, 2042 Fib). Briefly, following bisulfite conversion, a first PCR amplifies each region per sample in individual reactions, adding adapter sequences, as well as 8 random nucleotides (N8) for subsequent data deduplication. PCR conditions and primers for this first step are listed in Table S8. After pooling amplicons for each biological sample and clean-up using AMPure XP magnetic beads (#A63880, Beckman Coulter), a second PCR completes a sequence-ready

library with sample-barcodes for multiplexing. In this PCR reaction, barcoded Illumina adapters are attached to the pooled PCR samples ensuring that each sample pool receives a unique reverse barcoded adapter. Libraries were verified by running 1:30 dilutions on an Agilent bioanalyzer and then sequenced using the Illumina MiSeq platform to generate paired-end 250 bp reads using the indexing primer with the following sequence, 5'-AAGAGCGGTTCAGCAGGAATGCCGAGACCGATCTC-3' and 10% PhIX spike-in as the libraries are of low complexity.

IMPLICON bioinformatics analysis was also performed as described (Klobučar et al., 2020), following the step-by-step guide of data processing analysis. Briefly, data was processed using standard Illumina base-calling pipelines. As the first step in the processing, the first 8 bp of Read 2 were removed and written into the readID of both reads as an in-line barcode, or Unique Molecular Identifier (UMI). This UMI was then later used during the deduplication step with "deduplicate bismark–barcode mapped_file.bam". Raw sequence reads were then trimmed to remove both poor quality calls and adapters using Trim Galore v0.5.0 (Martin, 2011). Trimmed reads were aligned to the human reference genome in paired-end mode. Alignments were carried out with Bismark v0.20.0 and deduplication was then carried out with *deduplicate_bismark*, using the–barcode option to take UMIs into accountCpG methylation calls were extracted from the mapping output using the Bismark methylation extractor. Coverage files were imported into Seqmonk software v1.47 (Babraham Bioinformatics; RRID: SCR_001913) for all downstream analysis. Probes were made for each CpG contained within the amplicon and quantified using the DNA methylation pipeline or total read count options.

Trilineage Specification

Trilineage differentiation of F7cl15, F7cl4, 2041cl13, 2041cl2, 2042cl9 and 2042cl5 hiPSCs was performed using STEMdiff™ Trilineage Differentiation Kit (#05230, Stem Cell Technologies) to differentiate into ectodermal, mesodermal and endodermal lineages according to manufacturer's instructions. For ASD, F002, CE and CE hiPSCs, ectodermal differentiation was also performed. These experiments were conducted with at least one or two replicates (Fig. 5; Fig. S5). Briefly, a density of 200,000 (mesoderm lineage) or 800,000 (endoderm and/or ectoderm lineages) of cells were plated in 12-well plates on day 0. The medium was changed daily until day 5 (mesoderm and endoderm lineages) or day 7 (ectoderm lineage). After trilineage commitment, the cells were collected with NZYol reagent to perform RNA extraction followed by RT-qPCR or RNA-seq (for F7cl15/F7cl4 ectoderm differentiation) or dissociated with accutase to perform RNA FISH and RT-qPCR experiments. The primers used are listed in Table S8.

Cardiac Differentiation

The cardiac differentiation of ASD, F002, CD and CE hiPSCs was performed following a published protocol (Lian et al., 2012). Briefly, the hiPSCs were initially cultured in matrigel-coated plates in mTeSR1 medium (# 85850, STEMCELL Technologies) until full confluency. The differentiation was initiated by removing mTeSR1 medium and adding RPMI/B-27 without insulin (#A1895601, Thermo Fisher Scientific) and containing 6 µM of CHIR99021 (#04-0004, Stemgent), a GSK3 inhibitor. At day 3 (D3), to induce the cardiac fate of the mesendoderm progenitor cells, inhibition of canonical Wnt signaling is performed using IWP-4 (#72552, STEMCELL Technologies) a Wnt signaling inhibitor. Cardiac cells spontaneously develop into contracting cardiomyocytes when cultured in RPMI/B-27 medium and are left until day 30 of differentiation. At day 30 of differentiation (D30), the cardiomyocytes were collected with NZYol reagent to perform RNA extraction followed by RT-qPCR and RNA-AMP-seq using the primers listed in Table S8.

RNA AMPLICON-sequencing (RNA-AMP-seq) Library Preparation and Analysis

Total RNA was isolated, DNaseI treated and reverse-transcribed as described in the RT-qPCR section of the Methods. RNA-AMP-seq was performed for the samples before (ASD, F002, CD and CE hiPSCs) and after cardiac differentiation (ASD, F002, CD, CE hiPSC-CM) using a similar procedure previously employed for IMPLICON. Briefly, a first PCR amplifies each region per sample in individual reactions, adding adapter sequences, as well as 8 random nucleotides (N8) for subsequent data deduplication. PCR conditions and primers for this first step are listed in Table S8. After pooling amplicons for each biological sample and clean-up using AMPure XP magnetic beads, a second PCR completes a sequence-ready library with sample-barcodes for multiplexing. In this PCR reaction, barcoded Illumina adapters are attached to the pooled PCR samples ensuring that each sample pool receives a unique reverse barcoded adapter. Libraries were verified by running 1:30 dilutions on an Agilent bioanalyzer and then sequenced using the Illumina MiSeq platform to generate paired-end 250 bp reads using the indexing primer with the following sequence, 5'-AAGAGCGGTTCAGCAGGAATGCCGAGACCGATCTC-3' and 10% PhIX spike-in as the libraries are of low complexity.

RNA-AMP-seq data was first processed using standard Illumina base-calling pipelines. Briefly, the first 8 bp of Read 2 were removed and written into the readID of both reads as an in-line barcode, or Unique Molecular Identifier (UMI). This UMI was then later used during the deduplication step with “umi_tools dedup --umi-separator=':' -I "mapped_file.bam" --paired”. Raw sequence reads were then trimmed to remove both poor quality calls and adapters using Trim Galore v0.5.0 (doi: 10.5281/zenodo.5127899, Cutadapt version 1.15, parameters:–

paired). Trimmed reads were aligned to the human reference genome in paired-end mode. Alignments were carried out with STAR v2.7.11a. Deduplication was then carried out with UMI-tools v1.1 (see above). Aligned read (.bam) files were analyzed with phASER v.0.9.9.4 (Castel et al., 2016) for allelic expression quantification using the “phaser” and “phaser_gene_ae” commands.Minor Allelic Frequency (MAF) was then calculated as described above for the ASE analysis.

Statistics and Reproducibility

The statistical methods employed in each analysis are described in their respective sections. All these statistical tests were conducted using dedicated Python packages tailored to each specific analysis.

REFERENCES

- Bansal, P., Ahern, D.T., Kondaveeti, Y., Qiu, C.W., Pinter, S.F., 2021. Contiguous erosion of the inactive X in human pluripotency concludes with global DNA hypomethylation. Cell Rep. 35, 109215. https://doi.org/10.1016/j.celrep.2021.109215
- Bock, C., Reither, S., Mikeska, T., Paulsen, M., Walter, J., Lengauer, T., 2005. BiQ Analyzer: visualization and quality control for DNA methylation data from bisulfite sequencing. Bioinformatics 21, 4067–4068. https://doi.org/10.1093/bioinformatics/bti652
- Castel, S.E., Mohammadi, P., Chung, W.K., Shen, Y., Lappalainen, T., 2016. Rare variant phasing and haplotypic expression from RNA sequencing with phASER. Nat. Commun. 7, 12817. https://doi.org/10.1038/ncomms12817
- Dobin, A., Davis, C.A., Schlesinger, F., Drenkow, J., Zaleski, C., Jha, S., Batut, P., Chaisson, M., Gingeras, T.R., 2013. STAR: Ultrafast universal RNA-seq aligner. Bioinformatics 29, 15–21. https://doi.org/10.1093/bioinformatics/bts635
- Klobučar, T., Kreibich, E., Krueger, F., Arez, M., Pólvora-Brandão, D., von Meyenn, F., da Rocha, S.T., Eckersley-Maslin, M., 2020. IMPLICON: an ultra-deep sequencing method to uncover DNA methylation at imprinted regions. Nucleic Acids Res. 48, e92–e92. https://doi.org/10.1093/nar/gkaa567
- Lian, X., Hsiao, C., Wilson, G., Zhu, K., Hazeltine, L.B., Azarin, S.M., Raval, K.K., Zhang, J., Kamp, T.J., Palecek, S.P., 2012. Robust cardiomyocyte differentiation from human pluripotent stem cells via temporal modulation of canonical Wnt signaling. Proc. Natl. Acad. Sci. U. S. A. 109, E1848-1857. https://doi.org/10.1073/pnas.1200250109
- Love, M.I., Huber, W., Anders, S., 2014. Moderated estimation of fold change and dispersion for RNA-seq data with DESeq2. Genome Biol. 15, 550. https://doi.org/10.1186/s13059-014-0550-8
- McKenna, A., Hanna, M., Banks, E., Sivachenko, A., Cibulskis, K., Kernytsky, A., Garimella, K., Altshuler, D., Gabriel, S., Daly, M., DePristo, M.A., 2010. The Genome Analysis Toolkit: a MapReduce framework for analyzing next-generation DNA sequencing data. Genome Res. 20, 1297–1303. https://doi.org/10.1101/gr.107524.110
- Pólvora-Brandão, D., Joaquim, M., Godinho, I., Aprile, D., Álvaro, A.R., Onofre, I., Raposo, A.C., Pereira de Almeida, L., Duarte, S.T., da Rocha, S.T., 2018. Loss of hierarchical

imprinting regulation at the Prader–Willi/Angelman syndrome locus in human iPSCs. Hum. Mol. Genet. 27, 3999–4011. https://doi.org/10.1093/hmg/ddy274

- Poplin, R., Ruano-Rubio, V., DePristo, M.A., Fennell, T.J., Carneiro, M.O., Auwera, G.A.V. der, Kling, D.E., Gauthier, L.D., Levy-Moonshine, A., Roazen, D., Shakir, K., Thibault, J., Chandran, S., Whelan, C., Lek, M., Gabriel, S., Daly, M.J., Neale, B., MacArthur, D.G., Banks, E., 2018. Scaling accurate genetic variant discovery to tens of thousands of samples. https://doi.org/10.1101/201178
- Ramírez, F., Dündar, F., Diehl, S., Grüning, B.A., Manke, T., 2014. deepTools: a flexible platform for exploring deep-sequencing data. Nucleic Acids Res. 42, W187–W191. https://doi.org/10.1093/nar/gku365
- Rosspopoff, O., Cazottes, E., Huret, C., Loda, A., Collier, A.J., Casanova, M., Rugg-Gunn, P.J., Heard, E., Ouimette, J.-F., Rougeulle, C., 2023. Species-specific regulation of XIST by the JPX/FTX orthologs. Nucleic Acids Res. 51, 2177–2194. https://doi.org/10.1093/nar/gkad029
- Silva, T.P., Pereira, C.A., Oliveira, A.R., Raposo, A.C., Arez, M., Cabral, J.M.S., Milagre, I., Carmo-Fonseca, M., da Rocha, S.T., 2021a. Generation and characterization of induced pluripotent stem cells from a family carrying the BRCA1 mutation c.3612delA. Stem Cell Res. 52, 102242. https://doi.org/10.1016/j.scr.2021.102242
- Silva, T.P., Pereira, C.A., Raposo, A.C., Oliveira, A.R., Arez, M., Cabral, J.M.S., Milagre, I., Carmo-Fonseca, M., Rocha, S.T. da, 2021b. Generation and characterization of induced pluripotent stem cells heterozygous for the Portuguese BRCA2 founder mutation. Stem Cell Res. 53, 102364. https://doi.org/10.1016/j.scr.2021.102364
- Tukiainen, T., Villani, A.-C., Yen, A., Rivas, M.A., Marshall, J.L., Satija, R., Aguirre, M., Gauthier, L., Fleharty, M., Kirby, A., Cummings, B.B., Castel, S.E., Karczewski, K.J., Aguet, F., Byrnes, A., (…), Lappalainen, T., Regev, A., Ardlie, K.G., Hacohen, N., MacArthur, D.G., 2017. Landscape of X chromosome inactivation across human tissues. Nature 550, 244–248. https://doi.org/10.1038/nature24265
- Werner, J.M., Ballouz, S., Hover, J., Gillis, J., 2022. Variability of cross-tissue X-chromosome inactivation characterizes timing of human embryonic lineage specification events. Dev. Cell 57, 1995-2008.e5. https://doi.org/10.1016/j.devcel.2022.07.007
- Yokobayashi, S., Yabuta, Y., Nakagawa, M., Okita, K., Hu, B., Murase, Y., Nakamura, T., Bourque, G., Majewski, J., Yamamoto, T., Saitou, M., 2021. Inherent genomic properties underlie the epigenomic heterogeneity of human induced pluripotent stem cells. Cell Rep. 37, 109909. https://doi.org/10.1016/j.celrep.2021.109909
