# Supplementary material for: Gene reactivation upon erosion of X chromosome inactivation in female hiPSCs is predictable yet variable and persists through differentiation
Source: Stem Cell Reports. 2025 Apr 3;20(5):102472. doi: 10.1016/j.stemcr.2025.102472 (PMC12143139; doi:10.1016/j.stemcr.2025.102472)
Supplement: Document S2. Article plus supplemental information [file mmc4.pdf]

Stem Cell Reports
Article
OPEN ACCESS

Gene reactivation upon erosion of X chromosome inactivation in female hiPSCs is predictable yet variable and persists through differentiation

Ana Cláudia Raposo,[1,2,3,13] Paulo Caldas,[2,4,13] Joana Jeremias,[1,2] Maria Arez,[1,2] Francisca Cazaux Mateus,[1,2] Pedro Barbosa,[3,5] Rui Sousa-Luís,[3,6] Frederico Água,[2,4] David Oxley,[7] Annalisa Mupo,[8,9] Melanie Eckersley-Maslin,[10,11,12] Miguel Casanova,[1,2,3] Ana Rita Grosso,[2,4] and Simão Teixeira da Rocha[1,2,3,14,*]

[1]iBB - Institute for Bioengineering and Biosciences and Department of Bioengineering, Instituto Superior Técnico, Universidade de Lisboa, Lisbon, Portugal
[2]Associate Laboratory i4HB Institute for Health and Bioeconomy, Instituto Superior Técnico, Universidade de Lisboa, Lisbon, Portugal
[3]Instituto de Medicina Molecular, João Lobo Antunes, Faculdade de Medicina, Universidade de Lisboa, Lisbon, Portugal
[4]Department of Life Sciences, UCIBIO - Applied Molecular Biosciences Unit, NOVA School of Science and Technology, NOVA University Lisbon, Caparica, Portugal
[5]LASIGE, Departamento de Informática, Faculdade de Ciências, Universidade de Lisboa, 1749-016 Lisbon, Portugal
[6]Sir William Dunn School of Pathology, University of Oxford, Oxford, UK
[7]Mass Spectrometry Facility, The Babraham Institute, Cambridge, UK
[8]Epigenetics Programme, Babraham Institute, Cambridge CB22 3AT, UK
[9]Altos Labs, Cambridge, UK
[10]Peter MacCallum Cancer Centre, Melbourne, VIC 3000, Australia
[11]Sir Peter MacCallum Department of Oncology, The University of Melbourne, Melbourne, VIC 3010, Australia
[12]Department of Anatomy and Physiology, The University of Melbourne, Melbourne, VIC 3010, Australia
[13]These authors contributed equally
[14]Lead contact
*Correspondence: simao.rocha@tecnico.ulisboa.pt
https://doi.org/10.1016/j.stemcr.2025.102472

SUMMARY

Female human induced pluripotent stem cells frequently undergo X-chromosome inactivation (XCI) erosion, marked by X-inactive specific transcript (XIST) RNA loss and partial reactivation of the inactive X (Xi). This overlooked phenomenon limits our understanding of its impact on stem cell applications. Here, we show that XCI erosion is frequent and heterogeneous, leading to the reactivation of several X-linked genes. These are primarily located on the short arm of the X chromosome, particularly near escape genes and within H3K27me3-enriched domains, with reactivation linked to reduced promoter DNA methylation. Interestingly, escape genes further increase their expression from Xi upon XCI erosion, highlighting the critical role of XIST in their dosage regulation. Importantly, global (hydroxy) methylation levels and imprinted regions remain unaffected, and analysis of trilineage commitment and cardiomyocyte formation reveals that XCI erosion persists across differentiation. These findings underscore the need for greater awareness of the implications of XCI erosion for stem cell research and clinical applications.

INTRODUCTION

X-chromosome inactivation (XCI) in female mammals ensures dosage compensation between females (XX) and males (XY) through transcriptional silencing of one X chromosome (Loda et al., 2022; Patrat et al., 2020). This process is indispensable for female survival (Penny et al., 1996; Marahrens et al., 1997), being established in post-implantation embryos and faithfully maintained throughout life (Werner et al., 2022). The key regulator of XCI is the X-inactive specific transcript (XIST), a long non-coding RNA (lncRNA) that remains expressed only from the inactive X chromosome (Xi), which is randomly chosen for inactivation (reviewed in Patrat et al., 2020). XIST RNA coats the Xi and recruits several RNA binding proteins (RBPs) and chromatin modifiers, inducing a stable transcriptional silencing across the entire chromosome (da Rocha and Heard, 2017; Raposo et al., 2021). *XIST* remains expressed in all somatic cells, maintaining XCI with cell-specific nuances (Richart et al., 2022; Yu et al., 2021). Importantly, more than 15% of X-linked genes, known as escapees, evade inactivation on the Xi (Carrel and Willard, 2005; Tukiainen et al., 2017; Werner et al., 2022). Escapees could either constitutively evade inactivation (named escape genes or escapees in this article) or escape only in certain cell types or individuals (named variable genes).

Distinct from the *in vivo* situation, in clinically relevant female human pluripotent stem cells (hPSCs), namely embryonic stem cells (hESCs) and induced pluripotent stem cells (hiPSCs) derived and cultured in conventional/primed conditions, *XIST* expression is recurrently lost upon cell passage (Mekhoubad et al., 2012; Vallot et al., 2015). Loss of *XIST* expression leads to the progressive re-activation of the Xi, a process commonly known as XCI erosion (Bansal et al., 2021; Mekhoubad et al., 2012; Nazor et al., 2012; Sahakyan et al., 2018). The eroded X chromosome (Xe) is partially reactivated and is characterized by the loss of H3K27me3 histone modification across the Xi

Published by Elsevier Inc. on behalf of International Society for Stem Cell Research.
This is an open access article under the CC BY license (http://creativecommons.org/licenses/by/4.0/).

(Mekhoubad et al., 2012; Vallot et al., 2015) and DNA demethylation at the reactivated X-linked gene promoters (Geens et al., 2016; Patel et al., 2017; Bansal et al., 2021; Fukuda et al., 2021). XCI erosion can be easily tracked using genetic variants since hPSCs are clonal, meaning all cells share the same Xi, rather than being a mix of maternal or paternal X-inactivated cells (Tchieu et al., 2010).

Cell passage is the strongest driver for XCI erosion (Silva et al., 2008; Geens et al., 2016; Patel et al., 2017; Fukuda et al., 2021), with the rate of erosion being influenced by the culture conditions (Cloutier et al., 2022). Preventing or correcting XCI erosion is essential for maintaining proper X-linked gene dosage in female hPSCs. While xeno-free, feeder-dependent hESC medium minimizes erosion (Cloutier et al., 2022), prolonged culture may still increase vulnerability in these conditions. Targeting *XIST* promoter region by CRISPR-Cas9 gene editing can restore *XIST* expression and overcome erosion; however, this depends on the error-prone mechanism of non-homologous end joining (NHEJ) (Motosugi et al., 2022). XCI erosion can also be reversed by resetting hPSCs to a naive pre-XCI state, followed by a transition back to the primed state, but maintenance of XCI is limited to a certain number of passages (Patel et al., 2017; Agostinho de Sousa et al., 2023). Therefore, no current methods ensure permanent prevention or correction of XCI erosion.

X-linked gene activity of the Xe typically falls between the levels observed for Xa and the Xi (Bansal et al., 2021). In female naive hPSCs, besides the presence of two active X chromosomes (XaXa), these cells have a pronounced global decrease in DNA methylation levels and erasure of methylation marks at imprinted regions (Klobučar et al., 2020; Theunissen et al., 2016). While direct causality between the XaXa status and global demethylation has not yet been made for human PSCs, this association has been proven for female mouse XaXa ESCs (Choi et al., 2017). Interestingly, global DNA demethylation has been linked to advanced stages of XCI erosion in female hiPSCs (Bansal et al., 2021). Given the potential link between X-linked gene dosage and global DNA methylation, it is important to define how the degree of erosion impacts DNA methylation-dependent processes. This is particularly important for imprinted loci, which are often irreversibly dysregulated in hPSCs (Nazor et al., 2012; Bar and Benvenisty, 2019), with potentially impacting the functionality and fitness of their derivatives.

A key concern for hPSC clinical use is how XCI erosion affects differentiation. Research shows differences in differentiation ability and cell fate decisions between eroded and non-eroded hiPSCs. For instance, hiPSCs with no *XIST* expression exhibit more immature differentiation in teratoma assays (Anguera et al., 2012). Moreover, XCI erosion in hiPSCs influences neuronal maturation in cortical brain organoids (Motosugi et al., 2022) and cell fate decisions during cardiac differentiation (D'Antonio-Chronowska et al., 2019). XCI erosion also impacts the use of female hiPSCs with X-linked mutations for disease modeling by inadvertently activating the non-mutated allele on the Xi (Mekhoubad et al., 2012). It also remains unclear whether Xi erosion changes during differentiation, as studies show inconsistent findings on its maintenance, rescue, or amplification (Mekhoubad et al., 2012; Vallot et al., 2015; Patel et al., 2017; Motosugi et al., 2022; Cloutier et al., 2022).

Here, we explore the dynamics of erosion in a collection of female hiPSCs, including isogenic pairs with high levels (XIST+) or low levels of *XIST* (XIST−) expression. We employ RNA fluorescence *in situ* hybridization (FISH), Sanger sequencing, targeted amplicon-based allelic quantification, and RNA-seq to quantify allelic expression in these cells before and after differentiation. We unveiled that XCI erosion is frequent yet heterogeneous among our hiPSCs with no impact on the global (hydroxy)methylome and genomic imprinting. While not all X-linked genes are affected by erosion, specific features increase susceptibility, with escapees being particularly hypersensitive. Importantly, the variability of XCI status among hiPSCs persists through differentiation. Our findings emphasize the importance of drawing attention to XCI erosion within the stem cell community and advocate its inclusion in hiPSC quality control given their implications for their basic, translational, and clinical applications.

RESULTS

Impaired XCI is frequent in female hiPSCs

Erosion of XCI can have a major impact in the downstream applications of hiPSCs, making it crucial to evaluate the stability of the Xi. To address this, we took advantage of our collection of female hiPSCs (Burridge et al., 2011; Chamberlain et al., 2010; Gomes et al., 2020; Pólvora-Brandão et al., 2018; Silva et al., 2020), which comprises eight cell lines spanning a wide range of cell passages (P), including two isogenic pairs, ideal for epigenetic studies (Table S1). We began by assessing *XIST* expression as a proxy for erosion using quantitative reverse-transcription PCR (RT-qPCR), revealing distinct expression patterns: high levels (XIST+) in ASA and ASD hiPSCs (P16–20), intermediate levels (XIST±) in the F7 line (P37–39), and residual or absent *XIST* expression (XIST−) in F002 (P41–45), CD (P21–25), CE (P16–20), AG1-0 (P86–90), and iPSC6.2 (P85–87) lines (Figure 1A). Cell passage significantly impacted *XIST* downregulation, the first sign of XCI erosion, but it was also observed in lower-passage lines like CD and CE. Unlike XIST+ ASD line, *XIST* loss in CD and F002 lines correlated with increased methylation levels at YY1 binding sites in

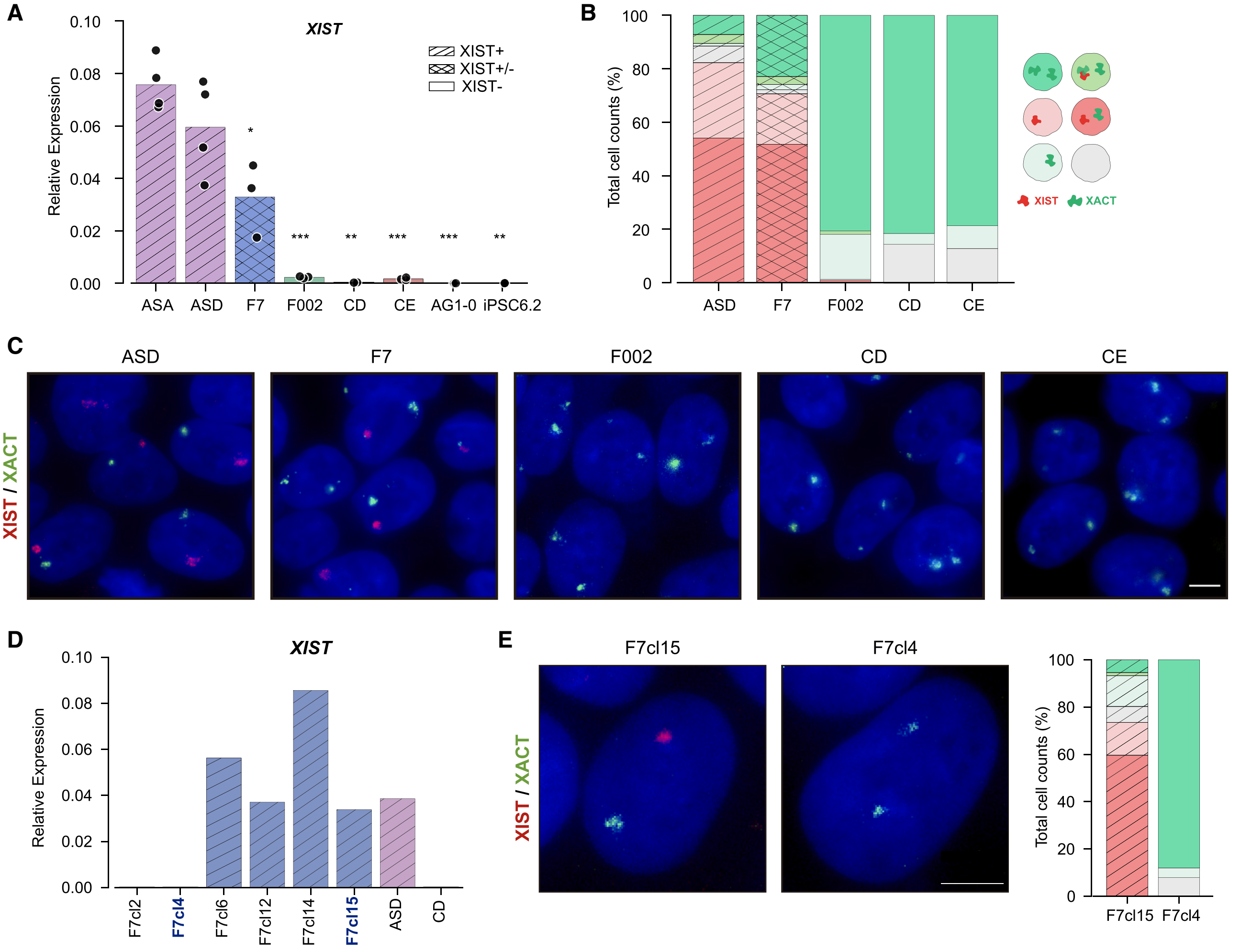

Figure 1. XCI status in female hiPSCs

(A) Barplot showing RT-qPCR analysis of *XIST* expression normalized to *GAPDH* housekeeping gene in female hiPSCs. Bars represent the average *XIST/GAPDH* relative expression. ASA, ASD, and AG1-0: $n = 4$; F7, F002, and CE: $n = 3$; CD and iPSC6.2: $n = 2$. Statistically significant differences between all iPSCs and ASA iPSCs are indicated as $*p < 0.01$; $**p < 0.001$; $***p < 0.0001$ (unpaired two-tailed Student's t test).

(B) Graph represents percentage of cells with different expression profiles for XIST and XACT by RNA FISH as depicted in the legend on the left; a minimum of 200 cells were counted per cell line.

(C) Representative RNA FISH images for XIST (red) and XACT (green) in ASD, F7, F002, CD, and CE hiPSCs; DNA stained in blue by DAPI; scale bar: 5 μm.

(D) Barplot with RT-qPCR analysis of *XIST* expression normalized to GAPDH housekeeping gene in newly generated F7 cell lines (F7cl2, F7cl4, F7cl6, F7cl12, F7cl14, and F7cl15). XIST+ ASD and XIST− CD cell lines were used as positive and negative controls, respectively; $n = 1$ for all iPSCs.

(E) On the left, representative RNA FISH images for XIST (red) and XACT (green) in isogenic F7cl15 and F7cl4 hiPSCs; DNA stained in blue by DAPI; scale bar: 5 μm; On the right, graph represents percentage of cells with different expression profiles for XIST and XACT by RNA FISH as depicted in (B); a minimum of 200 cells were counted per cell line.

XIST exon 1 (Figure S1A), a known *XIST* activator (Chapman et al., 2014; Fukuda et al., 2021; Makhlouf et al., 2014). To complement RT-qPCR analysis, we conducted single-cell RNA FISH for XIST and XACT. While XIST coats the Xi, XACT, another lncRNA, coats the Xa and Xe (Vallot et al., 2013, 2015). RNA FISH was performed on five cell lines: XIST+ (ASD), XIST− (F002, CD, and CE), and XIST± (F7). XIST was detected in >80% of XIST+ ASD, 70% of XIST± F7, and ~0% of XIST− (F002, CD, and CE) hiPSCs, confirming RT-qPCR results (Figures 1B and 1C). Conversely, >80% of XIST− hiPSCs showed biallelic XACT expression, characteristic of erosion. XIST+ ASD showed a few cells with biallelic XACT in the presence (3%) or absence of XIST expression (7%), a percentage

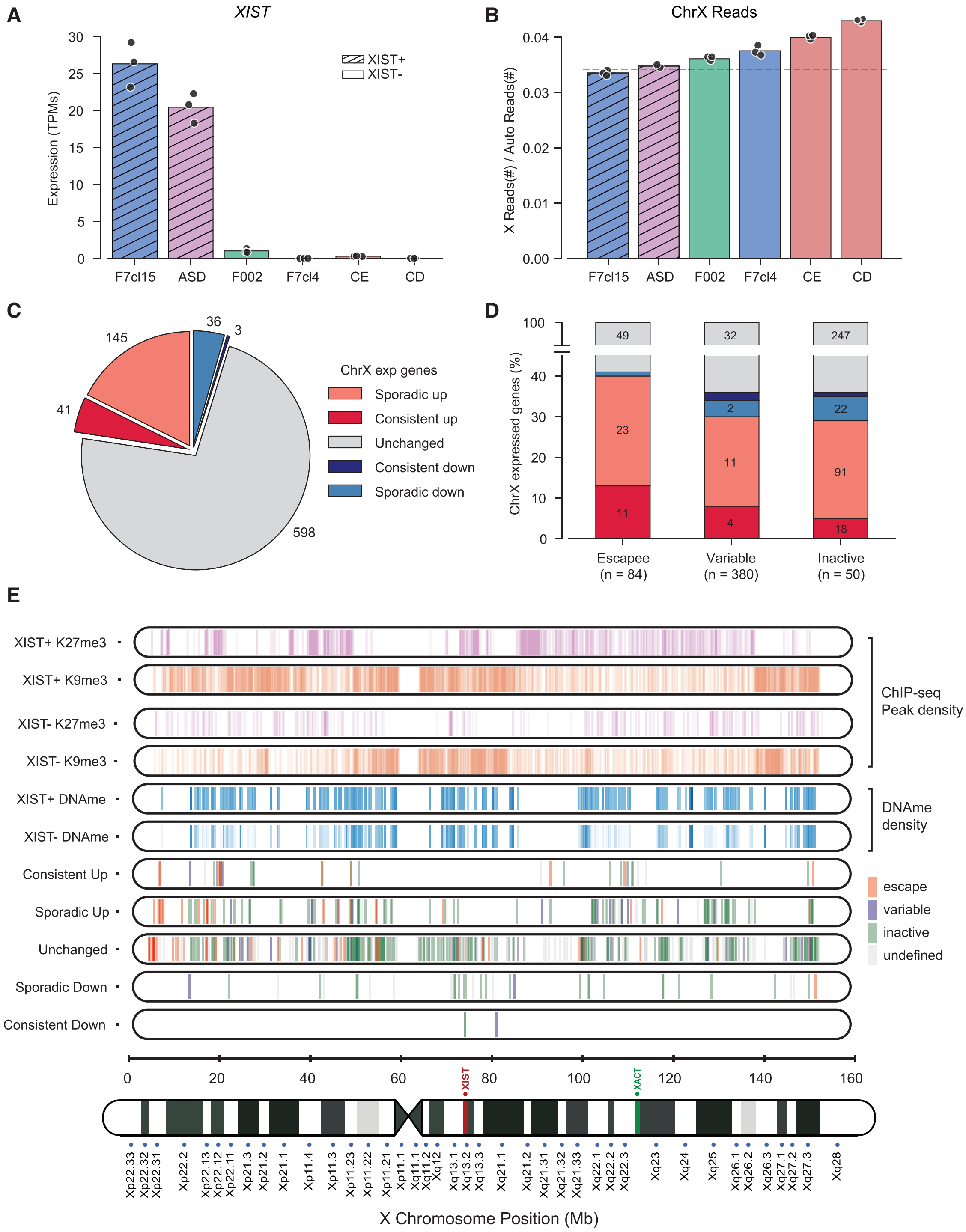

Figure 2. RNA-seq analysis reveals different degrees of XCI erosion in independent human iPSCs

(A) *XIST* expression analysis by RNA-seq in F7cl15, ASD, F002, F7cl4, CE, and CD hiPSCs. The graph shows the transcripts per million (TPMs) expression values from biological triplicates (black dots) of each sample.

(B) Barplot representing the ratio of normalized X chromosome/autosome reads by RNA-seq in F7cl15, ASD, F002, F7cl4, CE, and CD cell lines. The y axis shows the ratio between the number of chrX reads versus autosomal reads from the biological triplicates of each sample; dashed line represents the average ChrX/autosomal reads between the two XIST+ hiPSC lines, F7cl15 and ASD. Differences between

(legend continued on next page)

that increases to 25% in the XIST± F7 line (3% in the presence and 22% in the absence of XIST). Therefore, while XIST+ ASD is composed mostly of non-eroded cells, XIST± F7 presents a mixed population of non-eroded and eroded cells at roughly 75/25% ratio (Figures 1B and 1C).

Since epigenetic states are clonally propagated, we decided to isolate XIST+ and XIST− subclones from the F7 line. We picked 6 viable subclones and screened them for *XIST* expression by RT-qPCR to isolate 4 F7 XIST+ (F7cl6, F7cl12, F7cl14, and F7cl15) and two XIST− subclones (F7cl2 and F7cl4) (Figure 1D). We then selected one XIST+ (F7cl15) and one XIST− (F7cl4) subclones and analyzed the expression of *XIST* and *XACT* lncRNAs by RNA FISH (Figure 1E). As expected, *XIST* was detected in most cells in F7cl15, but not present in F7cl4 hiPSCs, while *XACT* was mostly monoallelic in F7cl15 and biallelic in F7cl4 hiPSCs (Figure 1E). In conclusion, our collection of hiPSC lines present different XCI states: ASD mostly preserves XCI and F002, CD, and CE hiPSCs show Xi erosion. The F7 line is a mosaic, containing both normal and eroded cells, from which XIST+ (e.g., F7cl15) and XIST− (e.g., F7cl4) subclones were successfully isolated.

While XCI loss reactivates some X-linked genes, the entire chromosome is not fully reactivated (Vallot et al., 2015; Theunissen et al., 2016; Bar and Benvenisty, 2019). Taking advantage of a panel of validated FISH probes targeting nascent transcripts, we investigated whether four X-linked genes, *HUWE1*, *ATRX*, *POLA1*, and *HDAC8*, are prone to reactivation upon XCI erosion. From these genes, we had prior knowledge that *POLA1* tends to be an eroded gene, while *ATRX* and *HUWE1* tend to be non-eroded genes (Vallot et al., 2015; Patel et al., 2017). The behavior of *HDAC8* was unknown at this stage. Our results show that *HUWE1*, *ATRX*, and *HDAC8* genes remain monoallelic in all hiPSC lines tested, regardless of XCI status (Figures S1B and S1C). This suggests that these genes are resistant to reactivation upon XCI erosion and continue to be expressed from the Xa. To further validate these findings, we took advantage of the common single-nucleotide polymorphism (SNP) rs3088074, present in the *ATRX* gene in all our hiPSC lines except for F002 (Figure S1D). We successfully showed that *ATRX* was always expressed from a single allele in each cell line not only confirming the monollelic expression of *ATRX* but also providing evidence of their clonal nature (Figure S1E). Interestingly, we observed that the isogenic CD and CE hiPSCs derived from the same skin biopsy express different *ATRX* alleles, indicating that they derived from somatic cells with a different Xi (Figure S1E). In contrast to *ATRX*, *HUWE1*, and *HDAC8*, *POLA1* exhibited a different behavior. In XIST+ cell lines, this gene shows monoallelic expression in most cells (ASD: 94%; F7cl15: 96%) while it was consistently biallelically expressed in XIST− cell lines, (F002: 93%, CD: 92% and CE: 82%). F7cl4 was an exception, with *POLA1* remaining mostly monoallelic (Figure S1C). These results suggest that *POLA1* is frequently reactivated from the Xe. Overall, our findings confirm the occurrence of XCI erosion in some of our female hiPSC lines. We also show that certain genes (*ATRX*, *HDAC8*, and *HUWE1*) resist this process, while others (*XACT* and *POLA1*) are more susceptible, with potential changes in the reactivation dynamics across different hiPSCs.

Different patterns of XCI erosion in female hiPSCs

To fully characterize the extent of XCI status, we performed bulk RNA sequencing (RNA-seq) on triplicates of our 2 XIST+ (F7cl15 and ASD) and 4 XIST− (F002, F7cl4, CD, and CE) hiPSCs. This includes isogenic pairs for XIST+ and XIST− (F7cl15 and F7cl4) and for XIST− (CD and CE) with distinct eroded X chromosomes. We first confirmed the expected pattern of *XIST* expression for each hiPSC line (Figure 2A) and that all hiPSC lines express high levels of pluripotency markers (Figure S2A). We next evaluated the ratio of RNA-seq reads mapping on the X versus autosomal reads (X:A ratio) as a proxy for erosion (Figure 2B). A statistically significant increase in the X:A ratio in XIST− hiPSCs was observed when compared to XIST+ hiPSC lines. Also, this increase varied substantially among different XIST− hiPSCs, ranging from 3.5% for the F002 (with residual *XIST* expression) to 24.6% for the CD (Figure 2B). To better understand how this variability translates at the gene level, we conducted pairwise differential gene

XIST− and XIST+ lines (ASD and F7cl15) are all significant (t test $p < 0.01$ and Cohen's d > 1). ASD vs. F7cl15 showed no statistical significance using the same standard.

(C) Pie chart depicting the number of consistently upregulated, sporadic upregulated, consistently downregulated, sporadic downregulated, and unchanged X-linked genes in XIST− hiPSCs when compared to XIST+ hiPSCs. Consistently up/down-regulated genes: differentially expressed in 3 to 4 XIST− hiPSCs; sporadic up/down-regulated genes: differentially expressed in 1 to 2 XIST− hiPSCs.

(D) Barplot illustrating the percentage of escape, variable, and inactive genes categorized according to Tukiainen et al., 2017; Werner et al., 2022; Tukiainen et al., 2017; and Werner et al., 2022, within consistently and sporadic upregulated, consistently and sporadic downregulated, and unchanged genes (a total of 823 expressed genes).

(E) Distribution of H3K9me3 and H3K27me3 ChIP-seq peaks (Yokobayashi et al., 2021) and DNA methylation level (Bansal et al., 2021) in XIST+ and XIST− cell lines, along with the position of all genes marked as escapee, variable, inactive, or undefined in each category (consistently up/down, sporadic up/down, and unchanged) along the X chromosome.

expression (DGE) analysis by comparing the different XIST− against both ASD and F7cl15 XIST+ hiPSCs (FDR < 0.05; −0.33 > logFC > 0.33). The low logFC threshold was chosen to capture subtle changes in genes either reactivating or increasing in expression from the Xi upon XCI erosion. As anticipated, XIST− hiPSCs exhibited a higher number of upregulated X-linked genes when compared to both XIST+ hiPSCs, while no such trend was observed for autosomal genes (Figures S2B and S2C). Consistent with the X:A ratio variation, the number of upregulated X-linked genes varies in XIST− hiPSCs, with the CD line showing the highest number of overexpressed X-linked genes (Figures S2B and S2C). We also noted an elevated number of upregulated genes in ASD compared to F7cl15 (Figures S2B and S2C), implying that the ASD line may exhibit initial signs of erosion. This is consistent with the lower *XIST* levels (Figure 2A) and the increased number of cells with biallelic *XACT* observed by RNA FISH compared to the F7cl15 line (Figures 1B and 1C). Overall, these findings suggest that XIST− hiPSCs can exhibit distinct levels of XCI erosion consistent with previous reports (Bansal et al., 2021; Yokobayashi et al., 2021).

Predictors of gene reactivation upon XCI erosion

We next investigated whether X-linked gene reactivation could be predicted upon XCI erosion. To classify gene behavior, we grouped X-linked genes by expression changes in XIST− vs. XIST+ hiPSCs. Genes upregulated in 3–4 lines were labeled “consistently upregulated,” while those upregulated in only 1–2 lines were classified as “sporadically upregulated.” Similarly, genes with decreased expression were categorized as “sporadically” or “consistently downregulated.” Genes with no consistent changes were deemed “unchanged.” This categorization identified 41 consistently upregulated, 145 sporadically upregulated, and 598 unchanged genes, with a few genes classified as consistently (3) or sporadically (36) downregulated, including *XIST* (Figure 2C and Table S2).

We first asked whether the original gene activity on the Xi prior to erosion could predict its upregulation upon *XIST* loss. For that, we categorized X-linked genes as inactive, variable (facultative escapees), or escape (constitutively escapees) genes according to Tukiainen et al. (2017) and reviewed by Werner et al. (2022). This categorization allowed us to examine whether pre-existing escape activity correlates with increased susceptibility to upregulation during XCI erosion. Among the 41 consistently upregulated genes, 34 could be categorized in these three classes (Figure S2D and Table S2). Of these, 35% (12 genes) were identified as escapees (e.g., *GYG2*, *NAP1L3*, and *TXLNG*). This proportion rises to 47% (16 genes) when variable genes are included. This represents a higher proportion compared to the percentage of escapees (∼15%) or the combined proportion of escapees and variable genes (∼25%) on the X chromosome. Consistently, nearly 40% of the escapees expressed in hiPSCs were either consistently or sporadically upregulated, while this proportion was lower for variable and inactive genes (Figure 2D). These findings indicate that escape genes, although partially evading *XIST*-dependent silencing on the Xi, are highly susceptible to upregulation upon the loss of *XIST*.

We next investigated how X chromosome positioning and epigenetic features, including histone marks and DNA methylation, influence X-linked gene upregulation in eroded hiPSCs. As observed in Figure 2E, consistently upregulated genes tend to localize in specific regions of the X chromosome, notably in the short arm (especially Xp22), as well as in the central portion of the long arm (Xq22 to Xq23). We compared these regions with Xi chromatin marks (H3K27me3 and H3K9me3) from chromatin immunoprecipitation sequencing (ChIP-seq) in XIST+ and XIST− hiPSCs (Yokobayashi et al., 2021) and found a predominant overlap between consistently upregulated genes and H3K27me3-rich domains (Figure 2E). To corroborate this observation, we plotted metagene profiles to visualize the distribution of H3K27me3/H3K9me3 marks across consistently upregulated, sporadically upregulated, and unchanged genes in XIST+ and XIST− hiPSCs. Consistently upregulated genes showed the highest H3K27me3 enrichment in XIST+ cells, followed by sporadically upregulated genes, while unchanged genes had the lowest enrichment. The differences between gene categories were statistically significant (Figure S2E; t test $p < 0.01$). Upon *XIST* loss, H3K27me3 was reduced for all genes as predicted. These data suggest that H3K27me3 enrichment in the Xi is a predictor of reactivation of X-linked genes upon erosion. In contrast, H3K9me3 did not seem to play a role in XCI erosion, in accordance with the literature (Vallot et al., 2015; Yokobayashi et al., 2021).

Next, we correlated loss of DNA methylation at promoters of X-linked genes with their categorization upon erosion. We leveraged DNA methylation data from Bansal et al. (2021) in XIST+ and XIST− iPSCs (see methods) to show pronounced loss of DNA methylation in consistently upregulated genes, moderate loss in sporadically upregulated genes, and minimal loss in unchanged genes (Figure S2F). The different behavior of each gene category was statistically significant ($p = 0.028$ for consistently vs. sporadic, $p < 0.001$ for sporadic vs. unchanged, using a t test).

Finally, given that escape genes are more prone to XCI erosion, we measure the distance of each gene to the closest upregulated escapee. We observed that consistently upregulated genes are significantly closer to these escapees compared to sporadically upregulated genes, which, in

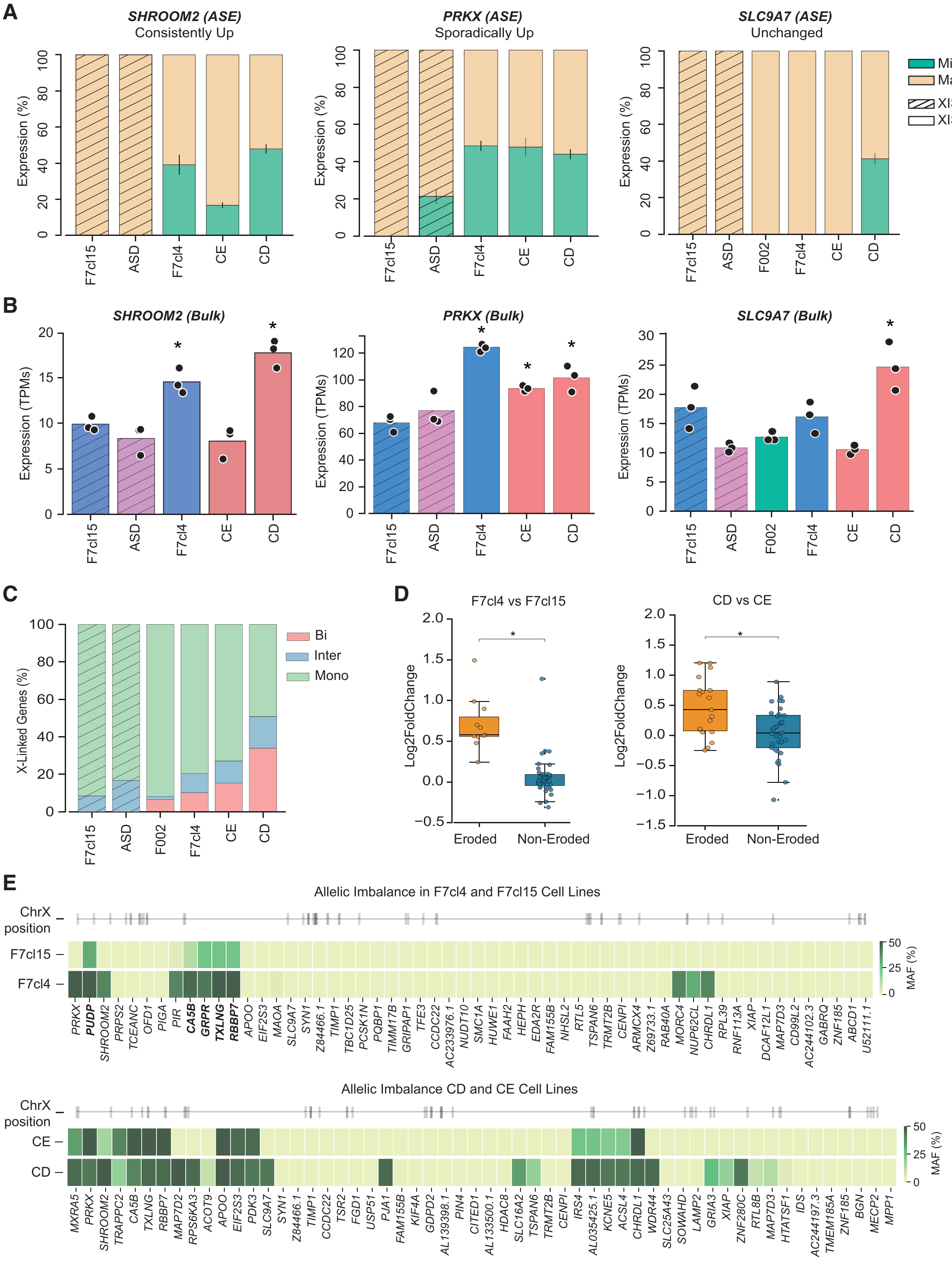

Figure 3. Eroded hiPSC lines show increasing number of biallelic gene expression

(A) Allele-specific expression (ASE) based on RNA-seq analysis from representative genes containing common SNPs across different hiPSCs. Bar plots displaying the average percentage of expression ±SEM of the major and minor alleles for the X-linked genes *SHROOM2*, *PRKX*, and *SLC9A7* in triplicates of XIST+ (F7cl15, ASD) and XIST− (F002, F7cl4, CE, and CD) hiPSCs.

(B) Corresponding RNA bulk expression based on RNA-seq analysis for these X-linked genes in the same XIST+ and XIST− hiPSCs shown in (A). Bar plots represent expression in TPMs (transcripts per million) for each gene in the different iPSC lines. Asterisks indicate statistical significance compared to either F7cl15 or ASD cell line (independent t test, p value < 0.05).

(legend continued on next page)

turn, are closer than unchanged genes (Figure S2G), suggesting a spatial influence on reactivation.

Overall, these findings confirm that X-linked genes vary in susceptibility to XCI erosion, with factors such as escapee status, proximity to escape genes, localization in Xp22 or Xq22-q23 regions, H3K27me3 enrichment, and reduced promoter methylation upon erosion significantly increasing the likelihood of erosion.

Allele-specific analysis reveals increased Xi expression after erosion, including escape genes

To correlate X-linked gene overexpression with the reactivation of genes from the Xe, we assessed allele-specific expression (ASE) from our RNA-seq dataset. Alleles on the two X chromosomes were discriminated using SNPs identified through whole exome sequencing (WES) of the cell lines included in our study (methods for details). By assessing phased haplotypes of SNPs, we found around 60 expressed heterozygous X-linked genes in each cell line (Table S3), some of which were present across different XIST+ and XIST− hiPSC lines, except for the F002 cell line that consistently showed fewer overlapping informative SNPs compared to the other lines. Biological replicates from three consecutive cell passages demonstrated remarkably consistent ASE of the various X-linked genes, suggesting a stable XCI status across replicates (Table S3). The original gene activity on the Xi in our XIST+ hiPSCs did not always align with the classifications from Tukiainen et al. (2017) and Werner et al. (2022) (Table S2). Notable examples included *CHRDL1* (here classified as inactive instead of escapee), *RBBP7* (here classified as escapee instead of inactive), and *PRKX* and *EIF2S3* (here classified as escapee or inactive depending on the XIST+ hiPSC line, rather than exclusively escapee) (Figures 3A and S3A). These minor discrepancies may result from the classification applied not being hiPSC specific.

In Figure 3A, we show some examples of X-linked genes with informative SNP across different cell lines. For instance, *SHROOM2,* a consistently upregulated gene, is expressed from only one allele in XIST+ cells but from both alleles in XIST− lines (Figure 3A, left panel). Similarly, *PRKX*, a sporadically upregulated gene, is monoallelic or allele biased in XIST+ cell lines (F7cl15 and ASD, respectively) but becomes equally expressed from both X chromosomes in eroded cell lines (Figure 3A, center panel). On the other hand, *SLC9A7*, an unchanged gene, is only reactivated in the most eroded CD cell line (Figure 3A, right panel). Importantly, the reactivation of the Xi is accompanied by an overall increased expression of these genes, as shown by bulk RNA-seq analysis (Figure 3B).

In general, the allelic expression analysis revealed four distinct behaviors of X-linked genes during erosion: (1) inactive genes in XIST+ cells that become biallelically expressed in all XIST− cells (e.g., *CHRDL1* and *SHROOM2*); (2) genes that are only re-expressed in the CD line (e.g., *SLC9A7*, *XIAP*, *TSPAN6*, and *MAP7D3*); (3) genes that remain monoallelically expressed across all cell lines (e.g., *CENPI* and *TRMT2B*); and (4) escape genes exhibiting biased allelic expression in at least one of the two XIST+ lines, which transition to balanced biallelic expression in XIST− cells (e.g., *PRKX*, *TXLNG*, *RBPP7*, and *EIF2S3*) (Figures 3A and S3A).

To further examine the behavior of escape genes, we conducted RNA FISH for three X-linked escapees (*TXLNG*, *RBBP7*, and *EIF2S3*) in two XIST+ hiPSC lines (F7cl15 and ASD) and two XIST− lines (F7cl4 and CD), enabling single-cell allele-specific analysis. Specific probes for each escape gene were combined with *XACT* as a marker of both Xa and Xe and *HUWE1* as a non-eroded X-linked gene marking the Xa. *XACT* was used instead of *XIST* since XIST− hiPSCs do not express *XIST*, whereas *XACT* would be expressed in both cell lines, mono- or biallelically. This experimental setup allowed us to categorize cells into four classes based on ASE of each escapee (Figures S3B and S3C): biallelic, monoallelic from the Xi/Xe, monoallelic from the Xa, and non-expressing cells. For all escapees, biallelic expression increased in XIST− hiPSCs, except for *EIF2S3* in F7cl15/F7cl4, which remained monoallelic from the Xa as expected from RNA-seq data (Figures S3A and S3C). Moreover, the bias toward monoallelic Xa expression seen in XIST+ cell lines was, to a certain extent, also attenuated in XIST− hiPSCs. In summary, these findings show that *XIST* loss in eroded cells promotes increased expression of escape genes from the Xe, suggesting that XIST plays a key role in limiting their expression on the Xi.

To analyze allelic expression globally, we divided X-linked genes in three classes based on the minor allele frequency (MAF), which represents the frequency of the

(C) Percentage of biallelic (Bi), intermediate (In-btw), and monoallelic (Mono) genes in XIST+ (F7cl15 and ASD) and XIST− (F002, F7cl4, CE, and CE) hiPSCs. Classes were defined based on the minor allele frequency (MAF) (methods for details).

(D) Differential gene expression analysis (DGEA) between F7cl4/F7cl15 and CD/CE isogenic pairs. Genes with at least 10% increase in their MAF were defined as eroded genes, while all the other expressed genes were considered non-eroded. Boxplots show the classification of each gene (eroded or non-eroded) against the log2 fold change obtained from the DGEA. Asterisks indicate statistical significance (p value < 0.01, Mann-Whitney U test).

(E) Minor allele frequency within prominent X-linked genes in isogenic hiPSC pairs, F7cl15/F7cl4 and CD/CE. Heatmap shows genes containing common SNPs in both isogenic cell lines, ordered by their position along the X chromosome (gray lines in the ideogram).

less common allele in each cell line: monoallelic (MAF ≤ 0.10), biallelic (MAF ≥ 0.40), and intermediate (0.10 < MAF < 0.40). As expected, the XIST+ cell lines (ASD and F7cl15) showed mostly monoallelic expression, with no biallelic expression (Figure 3C). Consistent with the increased X:A ratio observed in eroded hiPSCs (Figure 2B), we detected a higher number of intermediate and biallelic genes in eroded hiPSC lines, in the following order: F002 < F7cl4 < CE < CD. These findings suggest that the overexpression of X-linked genes in eroded hiPSCs is driven by the enhanced transcriptional activity from the Xe.

Next, we took advantage of our isogenic pairs (F7cl4/F7cl15 and CD/CE), which share the same SNPs, to conduct a more in-depth allele-specific analysis. To achieve this, we classified as "eroded genes" those showing at least a 10% increase in expression between the less eroded (F7cl15 and CE) and the most eroded line (F7cl4 and CD), while the remaining genes were classified as "non-eroded" (Figure 3D). Our results show that eroded genes exhibit higher bulk expression compared to non-eroded genes. This is manifested not only in XIST− F7cl4 vs. XIST+ F7cl15 cell lines but also in the CD vs. CE cells lines, which differ in their levels of erosion (Figure 3D). Not surprisingly, increased gene activity in the Xe maps to the short arm and the central portion of the long arm (Xq22 to Xq23) (Figure 3E), as previously observed for the overexpressed genes (Figure 2E). Notably, all escapees with available SNPs in XIST+ F7cl15 line (*PUPD*, *CA5B*, *GRPR*, *TXLNG*, and *RBBP7*) transitioned from an unequal allelic pattern to a more balanced allelic expression in XIST− F7cl4 hiPSCs (Figure 3E), in line with the previous observations. In summary, these findings demonstrate that the elevated gene expression in eroded hiPSC lines is primarily attributed to the increased transcription from the former Xi, with this effect intensifying with the degree of erosion, in accordance with previous studies (Mekhoubad et al., 2012; Vallot et al., 2015; Bar and Benvenisty, 2019; Bansal et al., 2021). Importantly, among the various genes on the X chromosome, escape genes are particularly sensitive to XIST loss, with their expression being upregulated upon XCI erosion. These findings highlight the critical role of XIST in restricting their expression from the Xi.

No impact of XCI erosion in (hydroxy)methylation or genomic imprinting

The dosage of X-linked genes is closely linked to overall levels of DNA methylation as shown for mouse ESCs (Choi et al., 2017). This association might also apply to hPSCs as these cells in naive conditions have two active X chromosomes and a global decrease in DNA methylation levels. Hence, given that XCI erosion leads to partial reactivation of the Xi, it is pertinent to evaluate DNA methylation levels in eroded hiPSCs. For this analysis, we have expanded our original cohort of hiPSCs with 7 new female iPSC lines from 3 different donors that were generated in the course of this study (Table S4) (Silva et al., 2021a, 2021b). We used the expression levels of *XIST* as a proxy for the XCI status, having the XIST± F7 line as a reference to separate what we classified as XIST+ and XIST− hiPSC lines. Out of the 7 new clones we have derived, 2 were classified as XIST+ and the remaining 5 as XIST− (Figure S4A). This includes hiPSC lines with low passage number (<P15) (Figure S4A and Table S4). We confirm the XCI status at a single-cell level by evaluating one of the XIST+ line (2042cl9) and one of the XIST− (2042cl1) hiPSC lines by RNA FISH for *XIST* and *XACT* (Figure S4B). From the initial set of XIST+ and XIST− female hPSC lines, we expanded to include three isogenic pairs, enhancing the robustness of our DNA methylation analysis (Tables S1 and S4).

To explore the potential variations in global DNA methylation levels between XIST+ and XIST− hiPSCs, we measured global 5-methylcytosine (5mC) and 5-hydroxymethylcytosine (5hmC) levels by liquid chromatography-tandem mass spectrometry (LC-MS/MS). No association between *XIST* expression and 5mC or 5hmC levels was observed in our cohort of female iPSCs, even for high-passage cells (Figures 4A and 4B). In line with this, our RNA-seq data showed no discernible differences in the expression levels of the *DUSP9* between XIST+ and XIST− cells, a gene previously identified as a pivotal X-linked factor influencing DNA methylation levels (Bansal et al., 2021; Choi et al., 2017) (Figure 4C). In conclusion, our study demonstrates that XCI erosion within our panel of female hiPSCs exerts no discernible influence on DNA (hydroxy)methylation levels.

An epigenetic anomaly frequently detected in hiPSCs, but varying across hiPSC lines, is imprinting defects (Bar et al., 2017, p. 2020; Klobučar et al., 2020). To investigate whether XCI erosion contributes to the heterogeneity of imprinting defects, we utilized IMPLICON, a targeted amplicon-seq method, to measure DNA methylation at imprinted regions (Klobučar et al., 2020; Arez et al., 2022) in two sets of isogenic XIST+ and XIST− hiPSCs, including the isogenic pairs CE and CD, which exhibit varying degrees of XCI erosion. The presence or absence of *XIST*, as well as the degree of erosion, does not affect methylation patterns at imprinted regions (Figure 4D). Indeed, abnormal gain of DNA methylation at *DLK1-DIO3*, *PEG3*, or *IGF2-H19* imprinted regions in hiPSCs, which is not seen in their somatic cells of origin (2042 and 2040 fibroblasts, 2042 and 2040 Fib), occurred independently of *XIST* expression (Figure 4D and Table S5). In conclusion, as for the global levels of DNA (hydroxy)methylation,

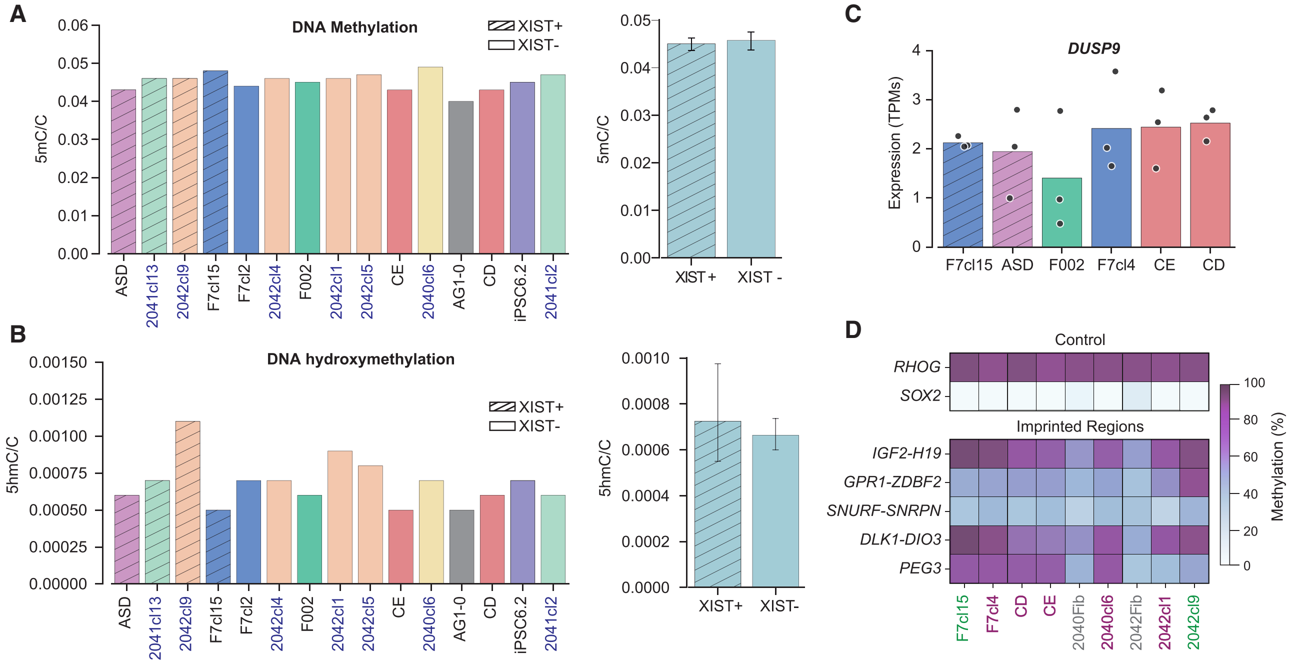

Figure 4. XCI erosion in our hiPSCs shows no impact on global DNA (hydroxy)methylation and genomic imprinting

(A and B) Barplots showing global 5mC (A) and 5hmC (B) levels measured by liquid chromatography-tandem mass spectrometry (LC-MS/MS). On the left, graph represents the ratio of 5mC or 5hmC per total cytosines in XIST+ (ASD, 2041cl13, 2042cl9, and F7cl15) and XIST− (F7cl2, 2042cl4, F002, 2042cl1, 2042cl5, CE, 2040cl6, AG1-0, CD, iPSC6.2, and 2041cl2) hiPSCs; $n = 1$ for all iPSCs; On the right, barplot shows the average ratio of 5mC or 5hmC ± SD per total cytosines ± SEM in XIST+ versus XIST− hiPSC lines.

(C) *DUSP9* expression data by RNA-seq in F7cl15, ASD, F002, F7cl4, CE, and CD hiPSCs. The graph shows the transcripts per million (TPMs) expression values from biological triplicates (black dots) of each sample.

(D) Heatmap representing the percentage of DNA methylation of the control loci (*RHOG* and *SOX2*) and imprinting control regions of several imprinted regions (*IGF2-H19*, *GPR1-ZDBF2*, *DLK1-DIO3*, *PWS/AS*, and *PEG3*) for F7cl15, F7cl4, CD, CE, 2040cl6, 2042cl1, and 2042cl9 iPSCs as well as 2040 and 2042 fibroblasts (fib). XIST+ and XIST− lines are colored in green and purple, respectively. Fibroblasts are colored in gray.

XCI erosion does not impact the methylation-dependent phenomenon of genomic imprinting in our iPSC lines.

Persistent XCI erosion throughout female iPSC trilineage specification

Next, we explored the dynamics of Xe during differentiation. We first conducted trilineage commitment experiments in three isogenic pairs of XIST+ and XIST− iPSCs: F7cl15 & F7cl4, 2041cl13 & 2041cl2, and 2042cl9 & 2042cl5. These hiPSC lines were differentiated into ectoderm, mesoderm, and endoderm for 5 to 7 days (Figure 5A). As expected, all hiPSC lines expressed the neuronal marker *PAX6*, while downregulating the pluripotent marker *NANOG* during ectoderm differentiation (Figure 5B). Likewise, all lines expressed *BRACHYURY* upon differentiation to mesoderm (Figure S5A). For the endoderm, we saw a tendency for the *SOX17* marker to be less expressed in XIST− hiPSCs, with the XIST− 2042cl5 even failing to express it (Figure S5A). Notably, *NANOG* expression remained detectable in all cell lines after endoderm specification (Figure S5A), suggesting suboptimal differentiation toward this lineage with this protocol. Overall, taken together, these data suggest that XCI erosion does not prevent trilineage specification.

We next evaluated *XIST* expression before and after trilineage commitment by RT-qPCR and RNA FISH. After 4–6 cell passages, XIST+ 2041cl13 and 2042cl9 hiPSC lines have undergone substantial erosion, reflected by a reduction in the number of XIST+ cells detected by RNA FISH (19% for 2041cl13 and 11% for 2042cl9) (Figures 5B and 5C). In contrast, XIST− hiPSCs maintained residual expression levels of *XIST* by both RT-qPCR and RNA FISH, while F7cl15 remained as a bona-fide XIST+ hiPSC line (~75% of XIST+ cells by RNA-FISH) (Figures 5B and 5C). Importantly, although the expression levels of *XIST* may rise during ectoderm differentiation, the number of *XIST*+ cells remained stable and was not rescued in XIST− hiPSC lines (Figures 5B, 5C, S5A, and S5B). Overall, XIST+ cell numbers remain stable during iPSC commitment to the three germ layers, regardless of their

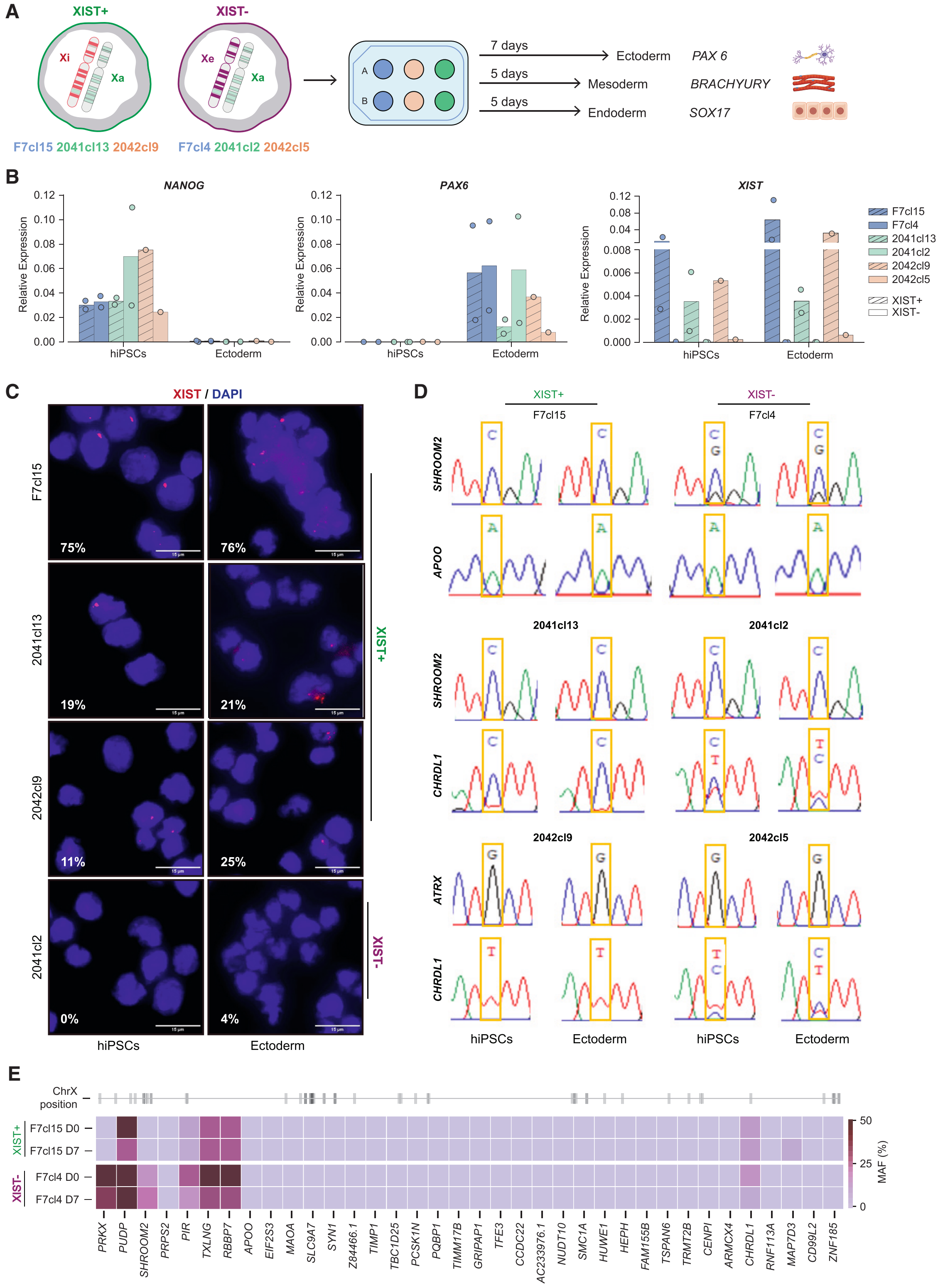

(legend on next page)

initial proportions. We then addressed the ASE of several X-linked genes in the differentiated progeny of these hiPSC lines expressing diverse levels of *XIST*. For that, we identified eight X-linked genes with common SNPs, including 6 in consistent and sporadic eroded genes (Table S6) and qualitatively assessed allelic expression using Sanger sequencing. Overall, the *XIST*+ counterpart of each isogenic pair retains more genes with monoallelic expression (Figures 5D and S5C and Table S6). Despite considerable loss of *XIST*, most of the assessed genes in the XIST+ 2041cl13 and 2042cl9 lines are not detected as biallelic in contrast to their XIST− counterparts. Strikingly, the original allelic expression profile found for each gene in hiPSCs, irrespective of their *XIST* expression profile, is maintained upon commitment to the three germ layers (Figures 5D and S5C, and Table S6). These results were also recapitulated for ectodermal differentiation of the XIST+ ASD and XIST− F002, CE, and CD hiPSC lines (Table S6).

To investigate the allelic evolution of the eroded state during differentiation in greater detail, we performed RNA-seq for ectodermal differentiation of the XIST+/XIST− F7cl15/F7cl4 pair. As anticipated, the expression levels of pluripotent markers (*NANOG*, *POU5F1*, and *PRDM14*) decreased upon differentiation, while ectoderm markers (*PAX6*, *NES*, and *OTX2*) increased (Figure S5D). Notably, *XIST* expression increased throughout differentiation in the F7cl15 line but remained absent in the F7cl4 line (Figure S5E). We compared allelic expression in F7cl15 and F7cl4 at D0 and D7 and found that biallelic genes retained their expression pattern, including eroded genes like *PRKX* and *SHROOM2* in XIST− F7cl4 (Figure 5E). Additionally, the proportion of biallelic, monoallelic, and "intermediate" genes remained mostly stable from D0 to D7, with a slight decrease in biallelic expression (Figure S5F). Overall, erosion patterns were neither rescued nor magnified during germ layer commitment.

Persistent XCI erosion throughout female iPSC cardiac differentiation

In addition to the short-term trilineage specification, we conducted a long-term cardiac differentiation protocol (Lian et al., 2012) on ASD, F002, CD, and CE lines (Figure 6A). After 15 days of differentiation, both XIST+ (ASD) and XIST− (F002, CD, and CE) cell lines were able to differentiate in contractile cardiomyocytes (Video S1). On day 30, we used RT-qPCR to confirm pluripotency marker downregulation (*POU5F1* and *NANOG*) and cardiac marker upregulation (*MYBPC3*) in all lines (Figure 6B). As expected, *XIST* remained expressed in ASD XIST+ cardiomyocytes and absent in XIST− hiPSCs (Figure 5B). To assess ASE of X-linked genes during cardiac differentiation, we developed a quantitative method, inspired by our prior work with IMPLICON (Klobučar et al., 2020). Our approach, named RNA-amplicon-sequencing (RNA-AMP-seq), integrates cDNA synthesis with amplicon sequencing and incorporates a de-duplication step to generate datasets with robust coverage and precise allelic discrimination for quantifying expression of targeted genes (methods for details). We focused on 9 X-linked genes with common SNPs in the population, including 4 consistently upregulated genes (*CHRDL1*, *GPC4*, *PDK3*, and *PCYT1B*), 1 sporadically upregulated (*APOO*), 2 unchanged (*PRPS2* and *HUWE1*), 1 consistently downregulated (*XIST*), and 1 escapee (*EIF2S3*) (Table S7). First, we validated this method by showing the enhanced sequence coverage offered comparing with RNA-seq in the same hiPSCs lines (Table S7). Next, we employed this method to assess the variation in allelic expression of X-linked genes with heterozygous SNPs between iPSCs and their

Figure 5. Erosion pattern persists upon ectodermal commitment

(A) Illustration of the experimental design used for trilineage commitment of the three XIST+/XIST− isogenic hiPSC pairs (F7cl15 & F7cl4, 2041cl13 & 2041cL2, and 2042cL9 & 2042cL5) into ectoderm, mesoderm, and endoderm. The inactive X chromosome (Xi) is marked in red, the active X chromosome (Xa) is marked in green, and the eroded X chromosome (Xe) is marked in purple.

(B) RT-qPCR analysis for *NANOG* (pluripotency marker), *PAX6* (neuronal marker), and *XIST* (Xi marker) normalized to *GAPDH* housekeeping gene in F7cl15, F7cl4, 2041cl13, 2041cl2, 2042cl9, and 2042cl5 hiPSCs and after ectoderm differentiation. Barplots represent the mean relative expression of $n = 2$ for all samples, except 2042cl9 and 2042cl5 in both hiPSCs and ectoderm ($n = 1$).

(C) Representative images of XIST RNA-FISH and respective percentages of cells expressing *XIST* (red dots) in F7cl15, 2041cl13, 2042cl9, and 2041cl2 at D0 (hiPSCs) and D7 (differentiated ectodermal cells). The nuclei are counterstained with DAPI (blue). Scale bars represent 15 μm. Number of cells counted: hiPSCs - F7cl15: 281, 2041cl13: 173, 2042cl9: 194, 2041cl2: 94; ectoderm cells - F7cl15: 324, 2041cl13: 326, 2042cl9: 367, 2041cl2: 381. The values represent 1 independent experiment.

(D) Allelic expression assayed by RT-PCR followed by Sanger sequencing resourcing to informative SNPs to distinguish the two alleles. The chromatograms represent illustrative examples of the allelic expression of heterozygous X-linked genes in each XIST+/XIST− isogenic hiPSC pair at D0 (hiPSCs) and at D7 (differentiated ectodermal cells): *SHROOM2* and *APOO* gene for the F7cl15 and F7cl4, *SHROOM2* and *CHRDL1* gene for 2041cl13 and 2041cl2, and *ATRX* and *CHRDL1* gene for 2042cl9 and 2042cl5. *CHRDL1* is not expressed in mesoderm cells.

(E) Minor allele frequency (MAF) in prevalent X-linked genes between naive (D0) and differentiated (D7) cell states in F7cl15 (XIST+) and F7cl4 (XIST−) cell lines. Heatmap shows genes containing common SNPs in both states, ordered by their position along the X chromosome (gray lines in the ideogram).

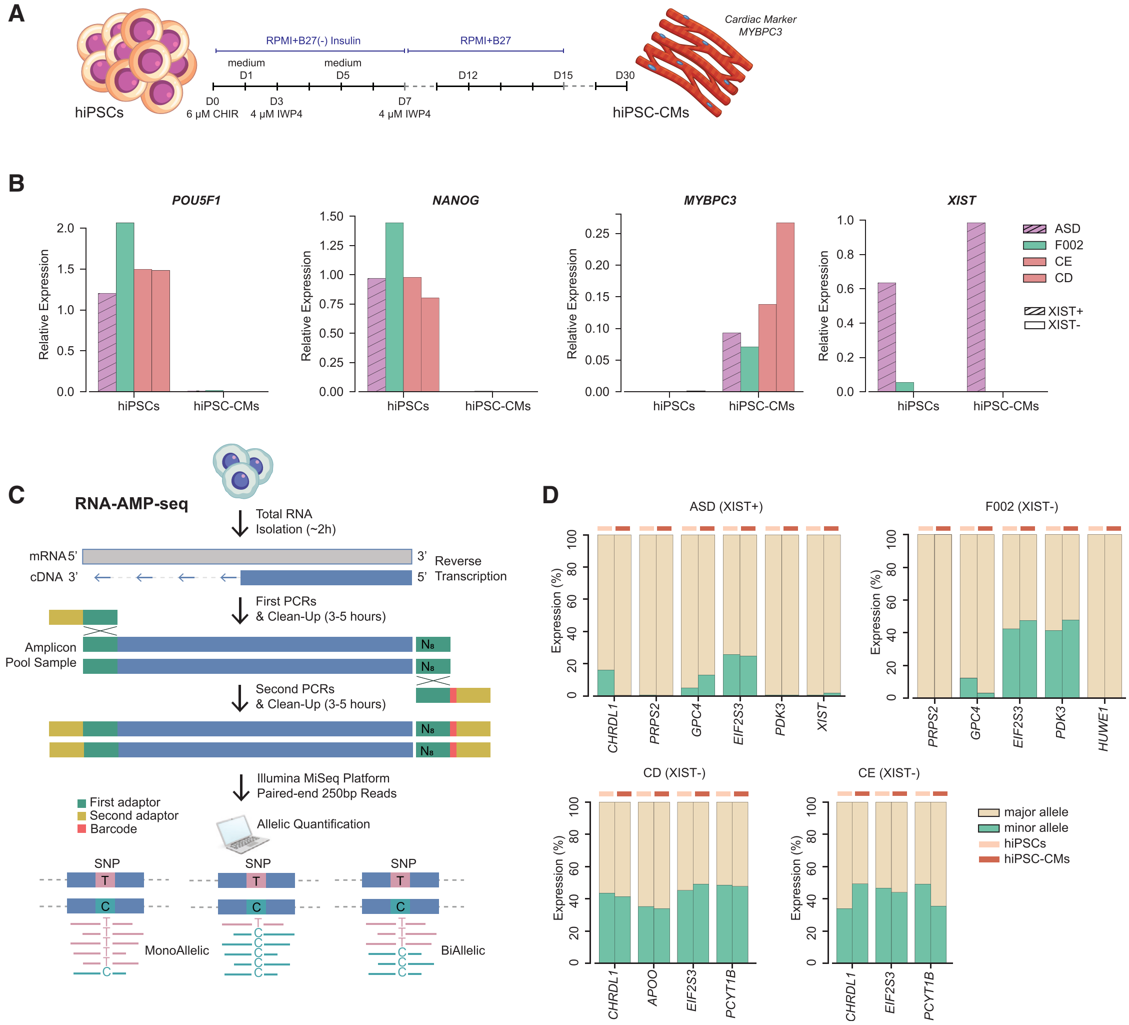

Figure 6. RNA-AMP-seq reveals maintenance of allelic ratio of X-linked genes upon cardiac differentiation of female hiPSCs

(A) Schematic overview of the cardiac differentiation protocol (see methods for details).

(B) Barplots with RT-qPCR analysis for *MYBPC3*, *NANOG*, *OCT4*, and *XIST* expression normalized to the *U6* housekeeping gene in ASD, F002, CE, and CD in hiPSCs and hiPSC-derived cardiomyocytes (hiPSC-CMs); $n = 1$ for all the samples.

(C) Illustration of our novel RNA-AMP-seq methodology. A first PCR amplifies each region per sample in individual reactions in the presence of adapter sequences and 8 random nucleotides (N8) for downstream deduplication of the data. After cleanup using AMPure XP magnetic beads, a second PCR completes a sequence-ready library with sample barcodes for multiplexing. Libraries were sequenced using the Illumina MiSeq platform to generate paired-end 250 bp reads (methods for more details).

(D) Allele-specific expression (ASE) based on RNA-AMP-seq analysis from representative genes containing common SNPs across different hiPSCs. Bar plots displaying the expression of the major and minor alleles for different X-linked genes in XIST+ (ASD) and XIST− (F002, CE, and CD) hiPSCs and hIPSC-CMs.

cardiomyocyte derivatives (hiPSC-CM). Remarkably, allelic expression ratios of X-linked genes remain stable in both XIST+ and XIST− hiPSCs upon differentiation (Figure 6D). Our results show that XCI erosion remains stable even after prolonged differentiation into functional cell types, consistent with our findings with the short-term trilineage differentiation and other distinct differentiation paradigms (Patel et al., 2017; Motosugi et al., 2022).

DISCUSSION

Our comprehensive exploration of XCI erosion in hiPSCs reveals four significant characteristics of this recurring phenomenon in female stem cell cultures: (1) XCI occurs frequently but exhibits high heterogeneity; (2) the likelihood of gene reactivation is influenced by the original gene activity in the Xi, its genomic location, and epigenetic

environment; (3) partial reactivation of the Xe does not usually induce changes in global DNA (hydroxy)methylation levels or affect the epigenetic profile of imprinted regions; and (4) the heterogeneous status of the Xe remains in differentiated derivatives of hiPSCs.

We observed that XCI erosion is common in female hiPSC cultures, with varying degrees observed across different cell lines. Our iPSCs were grown in mTSER Plus medium, which might not provide protection against erosion (Cloutier et al., 2022). Nevertheless, XCI erosion is observed in any of the most commonly used human stem cell media, including StemFlex, E8, or classical hESC medium under an irradiated feeder layer (Mekhoubad et al., 2012; Vallot et al., 2015; Motosugi et al., 2022; Agostinho de Sousa et al., 2023). Therefore, our observation that XCI erosion is common in female hiPSCs aligns with previous findings performed using other medium formulations. In addition to demonstrating the prevalence of XCI erosion, our data reveal their remarkable heterogeneity. This observation aligns with recent research (Bansal et al., 2021; Yokobayashi et al., 2021), yet we provide clear confirmation through ASE analysis. The variability observed underscores that XCI erosion encompasses a spectrum of states, adding complexity to this phenomenon. Although not comprehensively addressed by us in this study, the variable nature of XCI erosion might significantly impact downstream applications in both research and clinical settings. This underlines the critical need to incorporate XCI erosion assessment into routine hiPSC quality control protocols.

Despite the heterogeneity in XCI erosion, our study identifies specific traits influencing the likelihood of gene reactivation from the Xi. Location of a gene in the Xp22 or Xq22-q23 chromosomal regions, inclusion within H3K27me3-enriched domains, whether it is an escape gene or near one, and the loss of DNA methylation at promoters upon erosion all collectively increase the likelihood of reactivation, as previously reported (Vallot et al., 2015; Bar and Benvenisty, 2019; Bansal et al., 2021; Topa et al., 2024). This is reflected in our categorization of genes based on the consistency of their upregulation across different XIST− relative to XIST+ hiPSClines. This classification allowed us to assess the impact of XCI erosion and distinguish genes that are more prone to reactivation from those with stable silencing. The "consistently upregulated" genes likely represent loci that are particularly susceptible to XCI erosion, while "sporadically upregulated" genes may reflect variability in the extent of erosion or differences in the epigenetic landscape between cell lines. "Unchanged" genes highlight loci with robust silencing mechanisms that appear resistant to XCI erosion. Naturally, our classification captures the variability in gene behavior across the hiPSC lines included in our collection. It is possible that different cell lines might yield slightly different results. Nevertheless, this framework provides key insights into XCI erosion heterogeneity.

A noteworthy result of our study is the increased sensitivity of escape genes to XCI erosion. While initially counterintuitive, as these genes are known to escape silencing on the Xi, our allele-specific analysis shows a shift in escape gene behavior in XIST+ vs. XIST− hiPSCs. These genes, mostly showing biased expression from one allele in XIST+ cells, transitioned to equal expression from both alleles in XIST− cells. We demonstrate this through allele-specific RNA-seq analysis (Figures 3A and S3A) and validate it with single-cell RNA FISH for three escapees (Figures S3B and S3C). Consistent with a recent report linking escape gene susceptibility to *XIST* loss and XCI erosion (Topa et al., 2024), our findings provide the first single-cell evidence of heightened escape gene vulnerability to XCI erosion. This suggests that XIST lncRNA normally restricts escape gene expression on the Xi when compared to the Xa. Interestingly, a recent study also demonstrated preferential transcriptional upregulation of escape genes following conditional deletion of *Xist* in mouse embryonic fibroblasts and hematopoietic stem and progenitor cells (Yang et al., 2022). Both our findings and these reports support the notion that escape genes are attenuated in expression levels on Xi. This parallels recent observations in naive hESCs, where XIST dampens gene expression at the chromosomal scale (Alfeghaly et al., 2023; Dror et al., 2024). A key question for future studies is whether the XIST-dependent attenuation of gene expression in these two phenomena shares the same molecular mechanisms.

Our study also revealed that XCI erosion does not globally impact DNA methylation and hydroxymethylation levels or alter genomic imprinting patterns in hiPSCs. This was an important issue since naive hPSCs or XaXa mouse ESC/iPSCs have global hypomethylation and erasure of imprints (Theunissen et al., 2016; Klobučar et al., 2020; Arez et al., 2022). Despite the reactivation of specific X-linked genes, the overall (hydroxy)methylation profiles remained unchanged between XIST+ and XIST− hiPSC lines. In a previous study, while most eroded hiPSCs retained global DNA methylation levels comparable to XIST+ hiPSCs, advanced stages of XCI erosion in female hiPSCs were associated with global DNA demethylation (Bansal et al., 2021). This demethylation was linked to upregulation of the *DUSP9* gene, a key factor in explaining how X chromosome dosage impacts global methylation (Choi et al., 2017; Bansal et al., 2021). However, in our XIST− hiPSCs, even at high passages (Figure 4C), *DUSP9* was never found to be eroded, which may explain the absence of global DNA methylation changes (Figure 4A). Furthermore, recent findings, including a study analyzing

165 female hiPSCs, found no evidence of biallelic *DUSP9* expression (Topa et al., 2024). Based on these observations, we speculate that global DNA demethylation is a rare consequence of XCI erosion, likely occurring only under specific conditions. We also explored whether XCI erosion could contribute to the methylation variation at imprinted loci in hiPSCs (Bar and Benvenisty, 2019; Klobučar et al., 2020) and found no evidence that it affects DNA methylation patterns at these genes Therefore XCI erosion did not contribute for the imprinting errors observed in our hiPSCs.

Previous studies showed inconsistent findings showing either a rescue (Vallot et al., 2015) or maintenance of the XCI erosion pattern upon iPSC differentiation (Patel et al., 2017; Motosugi et al., 2022). These inconsistencies may be explained by the fact that no ASE was employed in these studies or was limited to a small number of genes. Our ASE analysis on different differentiation paradigms using isogenic sets of XIST+ and XIST− hiPSCs clearly point to the maintenance of the reactivated state of eroded genes during differentiation with a few exceptions. Therefore, the heterogeneous abnormal XCI states in female hiPSCs persist in their differentiated derivatives, potentially affecting cellular functionality and fitness (D'Antonio-Chronowska et al., 2019; Motosugi et al., 2022), though this was not explored in our study. For our ASE analysis, we used a qualitative method, Sanger sequencing, and two quantitative methods: RNA-seq and RNA-AMP-seq. While RNA-seq gives a comprehensive overview of the transcriptome, RNA-AMP-seq targets specific genetic variants on genes and builds datasets with allelic discrimination and higher coverage (Table S7). RNA-AMP-seq demonstrates promise as a high-throughput method for erosion screening. Compared to our current approach, which analyzes 9 X-linked genes (5 consistently/sporadically upregulated) across 8 samples, RNA-AMP-seq offers significant scalability to accommodate a much wider range of genes and samples. Additionally, the design of new primer pairs allows for straightforward customization, enabling the application of RNA-AMP-seq to other categories of monoallelically expressed genes. This includes genes subjected to imprinting or exhibiting random monoallelic expression.

In conclusion, our study advances the current understanding of XCI erosion in female hiPSCs and its implications for stem cell biology and regenerative medicine. By characterizing the frequency and persistence of XCI erosion, we provide valuable insights that can inform the development of improved hiPSC culture and differentiation protocols. Moving forward, continued research into the molecular mechanisms and the culture conditions driving XCI erosion will be essential for optimizing the utility of hiPSCs in various biomedical applications.

METHODS

A more detailed version of this section is available in the supplemental information.

Ethics

hiPSC lines were purchased or previously generated by us (Pólvora-Brandão et al., 2018; Silva et al., 2021a, 2021b) (Tables S1 and S4). Donor consent was obtained, and ethics approval granted by the Lisbon Academic Medical Center (Approval: 535/12, 170/18).

Stem cell culture

hiPSC lines were maintained in mTeSR Plus medium (STEMCELL Technologies) on Matrigel-coated plates (Corning), with media changes every 24–48 h. Cells were passaged using 0.5 mM EDTA (Invitrogen) in 1x PBS (Gibco) and cultured at 37°C in a 5% CO_2 incubator. Cells were dissociated with 0.5 mM EDTA/PBS, collected in washing medium (DMEM-F12, KSR, NEAA, L-Glutamine, β-mercaptoethanol), centrifuged, and resuspended in freezing medium (90% KSR, 10% DMSO) before storage in liquid nitrogen. iPSC cultures were routinely tested for mycoplasma contamination using the qPCR mycoplasma test (Mycoplasmacheck, Eurofins Genomics).

RT-qPCR

Total RNA was extracted from all hiPSC lines using NZYol RNA isolation reagent (NZYTech) and treated with DNase I (Roche) to remove contaminating DNA, following the manufacturer's instructions. DNase I-treated RNA (500 ng) was reverse-transcribed using random primers and a high-capacity cDNA reverse-transcription kit (Applied Biosystems). RT-qPCR was performed using NZYSpeedy qPCR Green Master Mix ROX (NZYTech) on StepOne or ViiA 7 real-time PCR systems (Applied Biosystems). Reactions were conducted in technical duplicates or triplicates and normalized to *GAPDH*. Primers are listed in Table S8. Data analysis was performed using StepOne or QuantStudio software, with relative gene expression calculated using the $2^{-\Delta\Delta CT}$ method.

PCR/RT-PCR followed by Sanger sequencing

To verify the presence of a specific SNP in ASD, F7, F002, CD, CE, 2041cl13, 2041cl2, 2042cl9, and 2042cl5 hiPSCs, genomic DNA was extracted using phenol:chloroform: isoamyl alcohol (Invitrogen) and amplified by PCR using primers listed in Table S8. For relative allelic expression analysis of X-linked genes in hiPSCs and their ectodermal, mesodermal, or endodermal derivatives, cDNA synthesized as described in the RT-qPCR section was amplified using the same primers. PCR products (DNA or cDNA) were

purified with the NZYGelpure kit (NZYTech) and sent for Sanger sequencing to STAB VIDA. Data were analyzed and visualized using Chromas v.2.6.2 software.

RNA FISH

The templates used for probe production were the following: XIST, a plasmid containing the 10 Kb exon 5–6 plasmid (Rosspopoff et al., 2023); *XACT*: RP11-35D3 bacterial artificial chromosome (BAC); *ATRX*: RP11-42M11 BAC; *HUWE1*: RP11-155O24 BAC; *HDAC8*: RP11-1021B19 BAC; *POLA1*: RP11-1104L9 BAC; *EIF2S3*: WI2-1347O20 fosmid; *TXLNG*: WI2-1095J6 fosmid; *RBBP7*: WI2-648C17 fosmid. Plasmid, fosmid or BAC probes were prepared using the Nick translation DNA labeling system 2.0 (Enzo) with red, green, or Cy5 dUTPs (red & green: ENZO; Cy5: Cytiva). RNA FISH was performed according to previously published protocol (Bousard et al., 2019). For probe preparation, 4 μL of probe was precipitated using NaAc (Sigma-Aldrich), sheared salmon sperm DNA (Invitrogen), human *COT1* DNA (Invitrogen), and 3 volumes of ethanol (Fisher Chemical). After denaturation (75°C, 7 min) and blocking (37°C, 30 min), probes were co-hybridized in a hybridization buffer overnight at 37°C. hiPSCs and differentiated cells were grown on matrigel-coated coverslips, fixed in 3% PFA, permeabilized with 0.5% Triton X-100, dehydrated in ethanol, and hybridized with fluorescent probes. After washing, nuclei were stained with DAPI and mounted. Imaging was performed using a Zeiss Axio Observer fluorescence microscope (63x oil objective). For RNA FISH analysis of escapees (*TLXNG*, *RBBP7*, and *EIF2S3*), only *HUWE1*/*XACT*-positive cells were counted (Figure S3B). At least 200 cells were analyzed per experiment.

Bisulfite sequencing

Genomic DNA was extracted using phenol:chloroform: isoamyl alcohol. Bisulfite treatment was performed with the EZ DNA Methylation Gold kit (Zymo Research). Bisulfite-treated DNA was PCR-amplified for YY1 binding sites in *XIST* exon 1 (Figure S1A) using primers in Table S8. PCR products were cloned into pGEM-T Easy (Promega), and at least 10 clones per sample were sequenced. Methylation analysis was done with BiQ Analyzer v.2.02 (Bock et al., 2005).

Whole Exome Sequencing (WES)

Genomic DNA (1.5 μg) from ASD, F7, F002, and CD was extracted using phenol:chloroform:isoamyl alcohol and sent to NOVOGENE. Libraries were prepared with the Agilent SureSelect Human All Exon V6 kit and paired-end sequenced on Illumina HiSeq 2000. Raw WES data were processed with Trim Galore, aligned to GRCh38 using BWA-MEM, and analyzed with GATK4 (Poplin et al., 2018) for duplicate marking, base recalibration, and variant calling. Variant calling (VCF) file was filtered (GQ > 30, DP > 20, MIN(FMT/AD > 7) and annotated with Ensembl VEP v.96 (McLaren et al., 2016). Chromosome X variants were selected for downstream analysis.

RNA-seq library preparation and analysis

RNA-seq was performed in triplicates for F7cl15, ASD, F002, F7cl4, CE, and CD hiPSC lines, plus one replicate of ectodermal differentiation (F7cl4/F7cl15 D0 & D7). Total RNA was extracted using NYZol, DNase-treated, and sent to NOVOGENE for quality control (RIN > 9) and sequencing on NovaSeq 6000. Reads were mapped to GRCh38 using STAR (–quantMode GeneCounts) (Dobin et al., 2013), and the number of raw reads mapping to the X chromosome (relative to total reads mapping to autosomes) was used as a proxy for erosion (Figure 2B). DESeq2 (v.1.40.2) (Love et al., 2014) was used to identify differentially expressed genes (DEGs) by establishing a threshold of $|\log2FC| = 0.33$ and an adjusted p value < 0.05. Transcripts per Million (TPMs) were used for gene expression quantification and hierarchical clustering.

We divided X-linked genes into five different categories according to their behavior in each comparison: consistently up/down-regulated if the gene was up/down-regulated in three or four cell lines when compared against both ASD and F7cl15 lines, sporadically up/down-regulated if the gene was up/down-regulated only in one or two cell lines when compared against both XIST+ controls, and unchanged if they did not meet any of the previous criteria. Examples of unchanged genes include genes considered up-regulated against F7cl15, but not against ASD, and genes upregulated against ASD, but downregulated against F7cl15. Furthermore, we categorized X-linked genes according to their XCI status as inactive, variable, and escapees following the classification by Werner et al., 2022 as reference and the classification from Tukiainen et al., 2017 for the remaining genes (Tukiainen et al., 2017; Werner et al., 2022).

Allele-Specific Expression (ASE) Analysis

Haplotype expression data were generated with phASER (v.0.9.9.4; Castel et al., 2016) using RNA-seq-based phasing. Only uniquely mapped reads (base quality ≥10) and loci with ≥10 read depth were analyzed to minimize false positives. Allelic imbalance was assessed using MAF, calculated as the ratio of minor allele read counts (the least common allele) to the total read counts from both alleles. Genes were classified as monoallelic (MAF < 0.10), biallelic (MAF > 0.40), or intermediate (0.10 ≤ MAF ≤ 0.40).

Re-analyzing epigenomic datasets: ChIP-seq and DNA methylation arrays

We integrated data from Yokobayashi et al. (2021) (GEO: GSE165869) and Bansal et al. (2021) to compare

H3K27me3, H3K9me3, and DNA methylation across the X chromosome in eroded vs. non-eroded conditions. ChIP-seq data from female samples F1 (eroded, XIST^{-}) and F3 (non-eroded, XIST^{+}) showed loss of H3K27me3 and H3K9me3 enrichment in confined regions during erosion. Metagene profiles of histone marks (Figure S2E) were generated using deepTools (v.3.5.1; Ramírez et al., 2014), normalizing signals across genomic regions with a 1 kbp bin size and 5 kbp gene body length.

DNA methylation data from Bansal et al. (2021) (Illumina 450K/850K arrays) was used to assess methylation changes during XCI erosion progression. The authors used probes with higher variance in female vs. male samples ($p \leq 0.01$) for K-means clustering, identifying six clusters based on methylation levels. The analysis revealed stepwise demethylation associated with Xi erosion. Here, we considered cluster A as the non-eroded (XIST^{+}) and cluster C as the eroded (XIST^{-}) state. To assess the correlation between gene upregulation and methylation variability, we analyzed promoter methylation across different gene categories. Two-sample t tests showed significant differences between consistently and sporadically upregulated genes ($p = 0.028$) and between sporadically upregulated and unchanged genes ($p < 0.001$) in the eroded state (cluster C).

Distances to escape genes

We computed the distance between each expressed gene and the nearest upregulated escapee using bedtools closest (Quinlan and Hall, 2010; v.2.30.0). Average distances for consistently upregulated, sporadically upregulated, unchanged, and downregulated genes were plotted. Mann-Whitney tests showed consistently upregulated genes differed significantly from all other categories ($p < 0.01$), while sporadically upregulated and unchanged genes were not ($p = 0.12$).

5mC/5hmC measurements by LC-MS

Genomic DNA was extracted using phenol:chloroform:isoamyl alcohol and digested with DNA Degradase Plus (Zymo Research). Nucleosides were analyzed by LC-MS/MS on a Q Exactive mass spectrometer (Thermo Scientific) with a nanoelectrospray ion source. Heavy isotope-labeled nucleosides were added before analysis. MS2 data for 5hmC, 5mC, and C were acquired using a 5 Th isolation window and fragmented by HCD (10% energy, 70,000 resolution). Quantification was done using extracted ion chromatograms and a six-point calibration curve, with triplicate runs for all samples and standards.

IMPLICON library preparation and analysis

IMPLICON was performed as described (Klobučar et al., 2020) on hiPSCs (F7cl15, F7cl4, CD, CE, 2040cl6, 2042cl1, and 2042cl9) and fibroblasts (2040 Fib and 2042 Fib). After bisulfite conversion, a first PCR amplified target regions per sample, adding adapter sequences and unique molecular identifiers (UMIs) for deduplication (Table S8). Amplicons were pooled, cleaned with AMPure XP beads (Beckman Coulter) and subjected to a second PCR to attach barcoded Illumina adapters. Libraries were quality-checked via Agilent bioanalyzer and sequenced on Illumina MiSeq (paired-end 250 bp) using the indexing primer, 5′-AAGAGCGGTTCAGCAGGAATGCCGAGACCGATCTC-3′, with a 10% PhiX spike-in. Computational analysis followed Klobučar et al. (2020). Illumina pipelines processed data, UMIs were extracted for deduplication, and reads were trimmed (Trim Galore v.0.5.0; (Martin, 2011)). Reads were aligned to the human genome using Bismark v.0.20.0 and deduplicated with UMIs, and CpG methylation calls were extracted. Coverage files were analyzed in Seqmonk v.1.47, with CpGs quantified using the DNA methylation pipeline or total read count method.

Trilineage specification and cardiac differentiation

hiPSCs (F7cl15, F7cl4, 2041cl13, 2041cl2, 2042cl9, and 2042cl5) were differentiated into ectoderm, mesoderm, and endoderm using the STEMdiff trilineage differentiation kit (STEMCELL Technologies). Ectodermal differentiation was also performed for ASD, F002, and CE hiPSCs. Cells were plated with a density of 200,000 (mesoderm) or 800,000 (endoderm/ectoderm) per well in 12-well plates. Media was changed daily until day 5 (mesoderm/endoderm) or day 7 (ectoderm). After differentiation, cells were collected for RNA extraction (RT-qPCR or RNA-seq) or dissociated for RNA FISH. Primers are listed in Table S8.

Cardiac differentiation of ASD, F002, CD, and CE hiPSCs followed protocol by Lian et al. (2012). hiPSCs were cultured to confluency in mTeSR1 on Matrigel-coated plates before differentiation with RPMI/B-27 (no insulin) and CHIR99021 (GSK3 inhibitor). At day 3, Wnt signaling was inhibited using IWP-4 to promote cardiac fate. Cells matured in RPMI/B-27 until day 30, when cardiomyocytes were collected for RNA extraction, RT-qPCR, and RNA-AMP-seq. Primers are listed in Table S8.

RNA-AMP-seq library preparation and analysis

Total RNA was isolated, DNase-treated, and reverse-transcribed as described in the RT-qPCR section. RNA-AMP-seq was performed on hiPSCs (ASD, F002, CD, and CE) before and after cardiac differentiation using a protocol similar to IMPLICON. A first PCR amplified target regions, adding adapters and UMIs. Amplicons were pooled, cleaned with AMPure XP beads, and subjected to a second PCR to attach barcoded Illumina adapters. Libraries were validated via Agilent bioanalyzer and sequenced on Illumina MiSeq (paired-end 250 bp) with a 10% PhiX

spike-in. Data were processed using Illumina pipelines. UMIs were extracted for deduplication; reads were trimmed (Trim Galore v.0.5.0), aligned to GRCh38 (STAR v.2.7.11a), and deduplicated with UMI-tools. Allelic expression was quantified with phASER v.0.9.9.4, and MAF was calculated as in ASE analysis.

RESOURCE AVAILABILITY

Lead contact

Requests for further information and resources should be directed to and will be fulfilled by the lead contact, Simão Teixeira da Rocha (simao.rocha@tecnico.ulisboa.pt).

Materials availability

This study did not generate new unique reagents.

Data and code availability

The datasets generated during the current study are available in the Gene Expression Omnibus (GEO): GSE262239. Source code and source data for most analyses in this study are available at GitHub: https://github.com/comicsfct/ErosionX.

ACKNOWLEDGMENTS

We would like to thank the previous and current members of S.T.d.R.'s team for helpful discussions. We also thank Felix Krueger, Maria Gouveia, Teresa Silva, Marta Furtado, and Adriana Vieira for technical help during the execution of this work. Work in S.T.d.R.'s team was supported by Fundação para a Ciência e a Tecnologia (FCT) Ministério da Ciência, Tecnologia e Ensino Superior (MCTES), Portugal (IC&DT projects PTDC/BIA-MOL/29320/2017 and 2022.01532.PTDC as well as projects UIDB/04565/2020 and UIDP/04565/2020 of the Research Unit Institute from Bioengineering and Biosciences – iBB and LA/P/0140/2020 of the Associate Laboratory Institute for Health and Bioeconomy – i4HB). A.C.R., M.A., and P.B. are supported, respectively, by SFRH/BD/137099/2018, SFRH/BD/151251/2021, and SFRH/BD/137062/2018 PhD fellowships from FCT/MCTES. P.C. was a recipient of a Marie Skłodowska-Curie Postdoctoral Fellowship (FOX-MTN-HORIZON-MSCA - 2021-PF-01-01) and an FCT Scientific Employment Stimulus Contract (2023.06750.CEECIND). S.T.d.R. was supported by an assistant research contract from FCT/MCTES (2021.00660.CEECIND/CP1651/CT0018).

AUTHOR CONTRIBUTIONS

A.C.R. performed the characterization of the majority of all hiPSCs and molecular biology experiments (RNA-seq, WES, RNA-FISH, and cardiac differentiation). P.C. conducted all bioinformatics analyses (RNA-seq, DGE, ASE, and all data visualizations). J.J. prepared and analyzed all the trilineage experiments (RNA-seq, RNA-FISH FISH, and Sanger sequencing) and RNA FISH experiments for escapees with the close assistance of F.C.M. J.J. also assisted in 5mC/5hmC measurements. M.A. prepared and analyzed the IMPLICON and RNA-AMP-seq experiments. P.B. mapped and analyzed the WES data, and R.S.-L. performed initial RNA-seq analyses. D.O. performed 5mC and 5hmC experiments. F.A. analyzed the RNA-AMP-seq results. A.M. assisted in the 5mC/5hmC measurements and IMPLICON. M.E.-M. supervised the analysis of the IMPLICON data. M.C. provided consulting on the experiments and critically revised the manuscript. A.R.G. supervised the bioinformatics analyses. S.T.d.R. conceived and supervised the study and secured funding. S.T.d.R., P.C., and A.C.R. interpreted the data and wrote the manuscript. All the authors have read and agreed to the published version of the manuscript.

DECLARATION OF INTERESTS

A.M. is an Altos Labs employee.

DECLARATION OF GENERATIVE AI AND AI-ASSISTED TECHNOLOGIES IN THE WRITING PROCESS

During the preparation of this work, the author(s) used ChatGPT in order to improve language and readability, with caution. After using this tool/service, the authors reviewed and edited the content as needed and take full responsibility for the content of the publication.

SUPPLEMENTAL INFORMATION

Supplemental information can be found online at https://doi.org/10.1016/j.stemcr.2025.102472.

Received: April 9, 2024
Revised: March 6, 2025
Accepted: March 7, 2025
Published: April 3, 2025

REFERENCES

Agostinho de Sousa, J., Wong, C.-W., Dunkel, I., Owens, T., Voigt, P., Hodgson, A., Baker, D., Schulz, E.G., Reik, W., Smith, A., et al. (2023). Epigenetic dynamics during capacitation of naïve human pluripotent stem cells. Sci. Adv. *9*, eadg1936. https://doi.org/10.1126/sciadv.adg1936.

Alfeghaly, C., Castel, G., Cazottes, E., Moscatelli, M., Moinard, E., Casanova, M., Boni, J., Mahadik, K., Lammers, J., Freour, T., et al. (2023). XIST dampens X chromosome activity in a SPEN-dependent manner during early human development. Preprint at bioRxiv. https://doi.org/10.1101/2023.10.19.563078.

Anguera, M.C., Sadreyev, R., Zhang, Z., Szanto, A., Payer, B., Sheridan, S.D., Kwok, S., Haggarty, S.J., Sur, M., Alvarez, J., et al. (2012). Molecular signatures of human induced pluripotent stem cells highlight sex differences and cancer genes. Cell Stem Cell *11*, 75–90. https://doi.org/10.1016/j.stem.2012.03.008.

Arez, M., Eckersley-Maslin, M., Klobučar, T., von Gilsa Lopes, J., Krueger, F., Mupo, A., Raposo, A.C., Oxley, D., Mancino, S., Gendrel, A.-V., et al. (2022). Imprinting fidelity in mouse iPSCs depends on sex of donor cell and medium formulation. Nat. Commun. *13*, 5432. https://doi.org/10.1038/s41467-022-33013-5.

Bansal, P., Ahern, D.T., Kondaveeti, Y., Qiu, C.W., and Pinter, S.F. (2021). Contiguous erosion of the inactive X in human pluripotency concludes with global DNA hypomethylation. Cell Rep. *35*, 109215. https://doi.org/10.1016/j.celrep.2021.109215.

Bar, S., and Benvenisty, N. (2019). Epigenetic aberrations in human pluripotent stem cells. EMBO J. *38*, e101033. https://doi.org/10.15252/embj.2018101033.

Bar, S., Schachter, M., Eldar-Geva, T., and Benvenisty, N. (2017). Large-Scale Analysis of Loss of Imprinting in Human Pluripotent Stem Cells. Cell Rep. *19*, 957–968. https://doi.org/10.1016/j.celrep.2017.04.020.

Bock, C., Reither, S., Mikeska, T., Paulsen, M., Walter, J., and Lengauer, T. (2005). BiQ Analyzer: visualization and quality control for DNA methylation data from bisulfite sequencing. Bioinformatics *21*, 4067–4068. https://doi.org/10.1093/bioinformatics/bti652.

Bousard, A., Raposo, A.C., Żylicz, J.J., Picard, C., Pires, V.B., Qi, Y., Gil, C., Syx, L., Chang, H.Y., Heard, E., and Da Rocha, S.T. (2019). The role of Xist -mediated Polycomb recruitment in the initiation of X-chromosome inactivation. EMBO Rep. *20*, e48019. https://doi.org/10.15252/embr.201948019.

Burridge, P.W., Thompson, S., Millrod, M.A., Weinberg, S., Yuan, X., Peters, A., Mahairaki, V., Koliatsos, V.E., Tung, L., and Zambidis, E.T. (2011). A Universal System for Highly Efficient Cardiac Differentiation of Human Induced Pluripotent Stem Cells That Eliminates Interline Variability. PLoS One *6*, e18293. https://doi.org/10.1371/journal.pone.0018293.

Carrel, L., and Willard, H.F. (2005). X-inactivation profile reveals extensive variability in X-linked gene expression in females. Nature *434*, 400–404. https://doi.org/10.1038/nature03479.

Castel, S.E., Mohammadi, P., Chung, W.K., Shen, Y., and Lappalainen, T. (2016). Rare variant phasing and haplotypic expression from RNA sequencing with phASER. Nat. Commun. *7*, 12817. https://doi.org/10.1038/ncomms12817.

Chamberlain, S.J., Chen, P.-F., Ng, K.Y., Bourgois-Rocha, F., Lemtiri-Chlieh, F., Levine, E.S., and Lalande, M. (2010). Induced pluripotent stem cell models of the genomic imprinting disorders Angelman and Prader–Willi syndromes. Proc. Natl. Acad. Sci. USA *107*, 17668–17673. https://doi.org/10.1073/pnas.1004487107.

Chapman, A.G., Cotton, A.M., Kelsey, A.D., and Brown, C.J. (2014). Differentially methylated CpG island within human XIST mediates alternative P2 transcription and YY1 binding. BMC. Genet. *15*, 89. https://doi.org/10.1186/s12863-014-0089-4.

Choi, J., Clement, K., Huebner, A.J., Webster, J., Rose, C.M., Brumbaugh, J., Walsh, R.M., Lee, S., Savol, A., Etchegaray, J.-P., et al. (2017). DUSP9 Modulates DNA Hypomethylation in Female Mouse Pluripotent Stem Cells. Cell Stem Cell *20*, 706–719.e7. https://doi.org/10.1016/j.stem.2017.03.002.

Cloutier, M., Kumar, S., Buttigieg, E., Keller, L., Lee, B., Williams, A., Mojica-Perez, S., Erliandri, I., Rocha, A.M.D., Cadigan, K., et al. (2022). Preventing erosion of X-chromosome inactivation in human embryonic stem cells. Nat. Commun. *13*, 2516. https://doi.org/10.1038/s41467-022-30259-x.

da Rocha, S.T., and Heard, E. (2017). Novel players in X inactivation: insights into Xist-mediated gene silencing and chromosome conformation. Nat. Struct. Mol. Biol. *24*, 197–204. https://doi.org/10.1038/nsmb.3370.

D'Antonio-Chronowska, A., Donovan, M.K.R., Young Greenwald, W.W., Nguyen, J.P., Fujita, K., Hashem, S., Matsui, H., Soncin, F., Parast, M., Ward, M.C., et al. (2019). Association of Human iPSC Gene Signatures and X Chromosome Dosage with Two Distinct Cardiac Differentiation Trajectories. Stem Cell Rep. *13*, 924–938. https://doi.org/10.1016/j.stemcr.2019.09.011.

Dobin, A., Davis, C.A., Schlesinger, F., Drenkow, J., Zaleski, C., Jha, S., Batut, P., Chaisson, M., and Gingeras, T.R. (2013). STAR: Ultrafast universal RNA-seq aligner. Bioinformatics *29*, 15–21. https://doi.org/10.1093/bioinformatics/bts635.

Dror, I., Chitiashvili, T., Tan, S.Y.X., Cano, C.T., Sahakyan, A., Markaki, Y., Chronis, C., Collier, A.J., Deng, W., Liang, G., et al. (2024). XIST directly regulates X-linked and autosomal genes in naive human pluripotent cells. Cellule *187*, 110–129.e31. https://doi.org/10.1016/j.cell.2023.11.033.

Fukuda, A., Hazelbaker, D.Z., Motosugi, N., Hao, J., Limone, F., Beccard, A., Mazzucato, P., Messana, A., Okada, C., San Juan, I.G., et al. (2021). De novo DNA methyltransferases DNMT3A and DNMT3B are essential for XIST silencing for erosion of dosage compensation in pluripotent stem cells. Stem Cell Rep. *16*, 2138–2148. https://doi.org/10.1016/j.stemcr.2021.07.015.

Geens, M., Seriola, A., Barbé, L., Santalo, J., Veiga, A., Dée, K., Van Haute, L., Sermon, K., and Spits, C. (2016). Female human pluripotent stem cells rapidly lose X chromosome inactivation marks and progress to a skewed methylation pattern during culture. Mol. Hum. Reprod. *22*, 285–298. https://doi.org/10.1093/molehr/gaw004.

Gomes, I., de Almeida, B.P., Dâmaso, S., Mansinho, A., Correia, I., Henriques, S., Cruz-Duarte, R., Vilhais, G., Félix, P., Alves, P., et al. (2020). Expression of receptor activator of NFkB (RANK) drives stemness and resistance to therapy in ER+HER2- breast cancer. Oncotarget *11*, 1714–1728. https://doi.org/10.18632/ONCOTARGET.27576.

Klobučar, T., Kreibich, E., Krueger, F., Arez, M., Pólvora-Brandão, D., von Meyenn, F., da Rocha, S.T., and Eckersley-Maslin, M. (2020). IMPLICON: an ultra-deep sequencing method to uncover DNA methylation at imprinted regions. Nucleic Acids Res. *48*, e92. https://doi.org/10.1093/nar/gkaa567.

Lian, X., Hsiao, C., Wilson, G., Zhu, K., Hazeltine, L.B., Azarin, S.M., Raval, K.K., Zhang, J., Kamp, T.J., and Palecek, S.P. (2012). Robust cardiomyocyte differentiation from human pluripotent stem cells via temporal modulation of canonical Wnt signaling. Proc. Natl. Acad. Sci. USA *109*, E1848–E1857. https://doi.org/10.1073/pnas.1200250109.

Loda, A., Collombet, S., and Heard, E. (2022). Gene regulation in time and space during X-chromosome inactivation. Nat. Rev. Mol. Cell. Biol. *23*, 231–249. https://doi.org/10.1038/s41580-021-00438-7.

Love, M.I., Huber, W., and Anders, S. (2014). Moderated estimation of fold change and dispersion for RNA-seq data with DESeq2. Genome Biol. *15*, 550. https://doi.org/10.1186/s13059-014-0550-8.

Makhlouf, M., Ouimette, J.-F., Oldfield, A., Navarro, P., Neuillet, D., and Rougeulle, C. (2014). A prominent and conserved role for YY1 in Xist transcriptional activation. Nat. Commun. *5*, 4878. https://doi.org/10.1038/ncomms5878.

Marahrens, Y., Panning, B., Dausman, J., Strauss, W., and Jaenisch, R. (1997). Xist-deficient mice are defective in dosage compensation

but not spermatogenesis. Genes Dev. *11*, 156–166. https://doi.org/10.1101/gad.11.2.156.

Martin, M. (2011). Cutadapt removes adapter sequences from high-throughput sequencing reads. EMBnet. j. *17*, 10–12. https://doi.org/10.14806/ej.17.1.200.

McLaren, W., Gil, L., Hunt, S.E., Riat, H.S., Ritchie, G.R.S., Thormann, A., Flicek, P., and Cunningham, F. (2016). The Ensembl Variant Effect Predictor. Genome Biol. *17*, 122. https://doi.org/10.1186/s13059-016-0974-4.

Mekhoubad, S., Bock, C., De Boer, A.S., Kiskinis, E., Meissner, A., and Eggan, K. (2012). Erosion of dosage compensation impacts human iPSC disease modeling. Cell Stem Cell *10*, 595–609. https://doi.org/10.1016/j.stem.2012.02.014.

Motosugi, N., Sugiyama, A., Okada, C., Otomo, A., Umezawa, A., Akutsu, H., Hadano, S., and Fukuda, A. (2022). De-erosion of X chromosome dosage compensation by the editing of XIST regulatory regions restores the differentiation potential in hPSCs. Cell Rep. Methods *2*, 100352. https://doi.org/10.1016/j.crmeth.2022.100352.

Nazor, K.L., Altun, G., Lynch, C., Tran, H., Harness, J.V., Slavin, I., Garitaonandia, I., Müller, F.J., Wang, Y.C., Boscolo, F.S., et al. (2012). Recurrent variations in DNA methylation in human pluripotent stem cells and their differentiated derivatives. Cell Stem Cell *10*, 620–634. https://doi.org/10.1016/j.stem.2012.02.013.

Patel, S., Bonora, G., Sahakyan, A., Kim, R., Chronis, C., Langerman, J., Fitz-Gibbon, S., Rubbi, L., Skelton, R.J.P., Ardehali, R., et al. (2017). Human Embryonic Stem Cells Do Not Change Their X Inactivation Status during Differentiation. Cell Rep. *18*, 54–67. https://doi.org/10.1016/j.celrep.2016.11.054.

Patrat, C., Ouimette, J.-F., and Rougeulle, C. (2020). X chromosome inactivation in human development. Dev. Camb. Engl. *147*, dev183095. https://doi.org/10.1242/dev.183095.

Penny, G.D., Kay, G.F., Sheardown, S.A., Rastan, S., and Brockdorff, N. (1996). Requirement for Xist in X chromosome inactivation. Nature *379*, 131–137. https://doi.org/10.1038/379131a0.

Pólvora-Brandão, D., Joaquim, M., Godinho, I., Aprile, D., Álvaro, A.R., Onofre, I., Raposo, A.C., Pereira de Almeida, L., Duarte, S.T., and da Rocha, S.T. (2018). Loss of hierarchical imprinting regulation at the Prader–Willi/Angelman syndrome locus in human iPSCs. Hum. Mol. Genet. *27*, 3999–4011. https://doi.org/10.1093/hmg/ddy274.

Poplin, R., Ruano-Rubio, V., DePristo, M.A., Fennell, T.J., Carneiro, M.O., Van der Auwera, G.A., Kling, D.E., Gauthier, L.D., Levy-Moonshine, A., Roazen, D., et al. (2018). Scaling accurate genetic variant discovery to tens of thousands of samples. Preprint at bioRxiv. https://doi.org/10.1101/201178.

Quinlan, A.R., and Hall, I.M. (2010). {BEDTools}: a flexible suite of utilities for comparing genomic features. Bioinformatics *26*, 841–842. https://doi.org/10.1093/bioinformatics/btq033.

Ramírez, F., Dündar, F., Diehl, S., Grüning, B.A., and Manke, T. (2014). deepTools: a flexible platform for exploring deep-sequencing data. Nucleic Acids Res. *42*, W187–W191. https://doi.org/10.1093/nar/gku365.

Raposo, A.C., Casanova, M., Gendrel, A.-V., and da Rocha, S.T. (2021). The tandem repeat modules of Xist lncRNA: a swiss army knife for the control of X-chromosome inactivation. Biochem. Soc. Trans. *49*, 2549–2560. https://doi.org/10.1042/BST20210253.

Richart, L., Picod-Chedotel, M.L., Wassef, M., Macario, M., Aflaki, S., Salvador, M.A., Héry, T., Dauphin, A., Wicinski, J., Chevrier, V., et al. (2022). XIST loss impairs mammary stem cell differentiation and increases tumorigenicity through Mediator hyperactivation. Cell *185*, 2164–2183.e25. https://doi.org/10.1016/j.cell.2022.04.034.

Rosspopoff, O., Cazottes, E., Huret, C., Loda, A., Collier, A.J., Casanova, M., Rugg-Gunn, P.J., Heard, E., Ouimette, J.-F., and Rougeulle, C. (2023). Species-specific regulation of XIST by the JPX/FTX orthologs. Nucleic Acids Res. *51*, 2177–2194. https://doi.org/10.1093/nar/gkad029.

Sahakyan, A., Yang, Y., and Plath, K. (2018). The Role of Xist in X-Chromosome Dosage Compensation. Trends Cell Biol. *28*, 999–1013. https://doi.org/10.1016/j.tcb.2018.05.005.

Silva, S.S., Rowntree, R.K., Mekhoubad, S., and Lee, J.T. (2008). X-chromosome inactivation and epigenetic fluidity in human embryonic stem cells. Proc. Natl. Acad. Sci. USA *105*, 4820–4825. https://doi.org/10.1073/pnas.0712136105.

Silva, T.P., Bekman, E.P., Fernandes, T.G., Vaz, S.H., Rodrigues, C.A.V., Diogo, M.M., Cabral, J.M.S., and Carmo-Fonseca, M. (2020). Maturation of Human Pluripotent Stem Cell-Derived Cerebellar Neurons in the Absence of Co-culture. Front. Bioeng. Biotechnol. *8*, 70. https://doi.org/10.3389/fbioe.2020.00070.

Silva, T.P., Pereira, C.A., Oliveira, A.R., Raposo, A.C., Arez, M., Cabral, J.M.S., Milagre, I., Carmo-Fonseca, M., and da Rocha, S.T. (2021a). Generation and characterization of induced pluripotent stem cells from a family carrying the BRCA1 mutation c.3612delA. Stem Cell Res. *52*, 102242. https://doi.org/10.1016/j.scr.2021.102242.

Silva, T.P., Pereira, C.A., Raposo, A.C., Oliveira, A.R., Arez, M., Cabral, J.M.S., Milagre, I., Carmo-Fonseca, M., and Rocha, S.T.d. (2021b). Generation and characterization of induced pluripotent stem cells heterozygous for the Portuguese BRCA2 founder mutation. Stem Cell Res. *53*, 102364. https://doi.org/10.1016/j.scr.2021.102364.

Tchieu, J., Kuoy, E., Chin, M.H., Trinh, H., Patterson, M., Sherman, S.P., Aimiuwu, O., Lindgren, A., Hakimian, S., Zack, J.A., et al. (2010). Female human iPSCs retain an inactive X chromosome. Cell Stem Cell *7*, 329–342. https://doi.org/10.1016/j.stem.2010.06.024.

Theunissen, T.W., Friedli, M., He, Y., Planet, E., O'Neil, R.C., Markoulaki, S., Pontis, J., Wang, H., Iouranova, A., Imbeault, M., et al. (2016). Molecular Criteria for Defining the Naive Human Pluripotent State. Cell Stem Cell *19*, 502–515. https://doi.org/10.1016/j.stem.2016.06.011.

Topa, H., Benoit-Pilven, C., Tukiainen, T., and Pietiläinen, O. (2024). X-chromosome inactivation in human iPSCs provides insight into X-regulated gene expression in autosomes. Genome Biol. *25*, 144. https://doi.org/10.1186/s13059-024-03286-8.

Tukiainen, T., Villani, A.-C., Yen, A., Rivas, M.A., Marshall, J.L., Satija, R., Aguirre, M., Gauthier, L., Fleharty, M., Kirby, A., et al. (2017). Landscape of X chromosome inactivation across human tissues. Nature *550*, 244–248. https://doi.org/10.1038/nature24265.

Vallot, C., Huret, C., Lesecque, Y., Resch, A., Oudrhiri, N., Bennaceur-Griscelli, A., Duret, L., and Rougeulle, C. (2013). XACT, a long noncoding transcript coating the active X chromosome in human pluripotent cells. Nat. Genet. *45*, 239–241. https://doi.org/10.1038/ng.2530.

Vallot, C., Ouimette, J.F., Makhlouf, M., Féraud, O., Pontis, J., Côme, J., Martinat, C., Bennaceur-Griscelli, A., Lalande, M., and Rougeulle, C. (2015). Erosion of X chromosome inactivation in human pluripotent cells initiates with XACT coating and depends on a specific heterochromatin landscape. Cell Stem Cell *16*, 533–546. https://doi.org/10.1016/j.stem.2015.03.016.

Werner, J.M., Ballouz, S., Hover, J., and Gillis, J. (2022). Variability of cross-tissue X-chromosome inactivation characterizes timing of human embryonic lineage specification events. Dev. Cell. *57*, 1995–2008.e5. https://doi.org/10.1016/j.devcel.2022.07.007.

Yang, T., Ou, J., and Yildirim, E. (2022). Xist exerts gene-specific silencing during XCI maintenance and impacts lineage-specific cell differentiation and proliferation during hematopoiesis. Nat. Commun. *13*, 4464. https://doi.org/10.1038/s41467-022-32273-5.

Yokobayashi, S., Yabuta, Y., Nakagawa, M., Okita, K., Hu, B., Murase, Y., Nakamura, T., Bourque, G., Majewski, J., Yamamoto, T., and Saitou, M. (2021). Inherent genomic properties underlie the epigenomic heterogeneity of human induced pluripotent stem cells. Cell Rep. *37*, 109909. https://doi.org/10.1016/j.celrep.2021.109909.

Yu, B., Qi, Y., Li, R., Shi, Q., Satpathy, A.T., and Chang, H.Y. (2021). B cell-specific XIST complex enforces X-inactivation and restrains atypical B cells. Cell *184*, 1790–1803.e17. https://doi.org/10.1016/j.cell.2021.02.015.

Stem Cell Reports, Volume *20*

Supplemental Information

Gene reactivation upon erosion of X chromosome inactivation in female hiPSCs is predictable yet variable and persists through differentiation

Ana Cláudia Raposo, Paulo Caldas, Joana Jeremias, Maria Arez, Francisca Cazaux Mateus, Pedro Barbosa, Rui Sousa-Luís, Frederico Água, David Oxley, Annalisa Mupo, Melanie Eckersley-Maslin, Miguel Casanova, Ana Rita Grosso, and Simão Teixeira da Rocha

SUPPLEMENTARY FIGURE 1

A

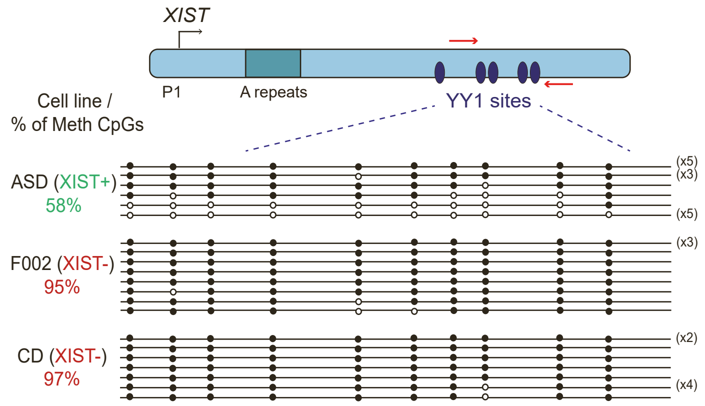

B

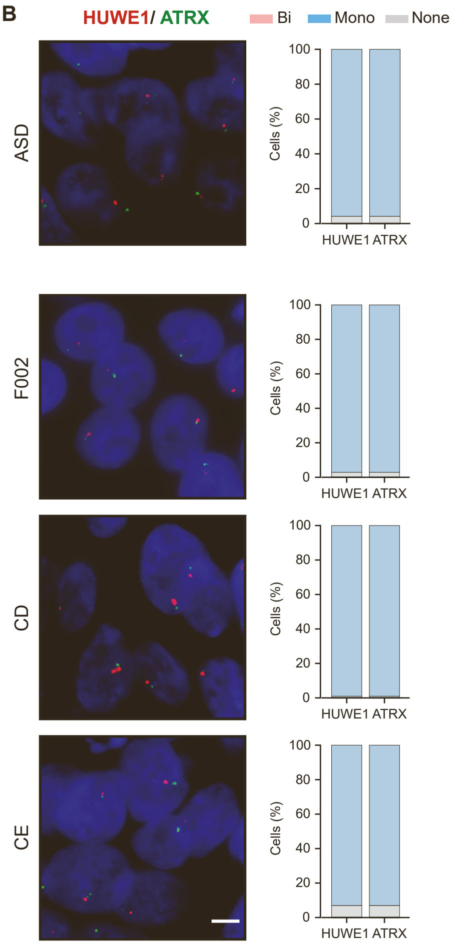

C

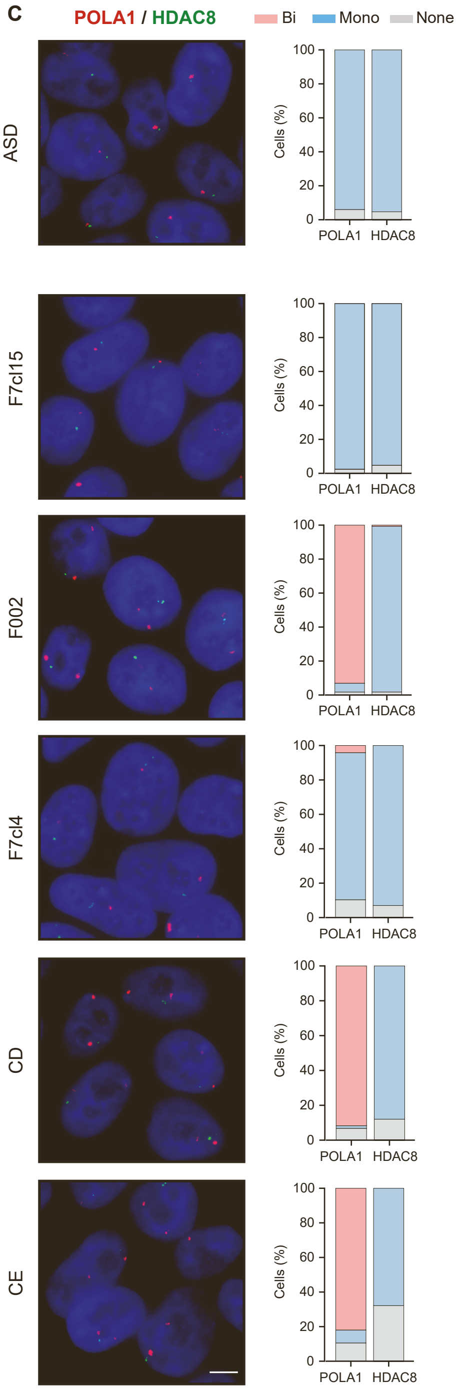

D

Cell lines	XCI status	DNA<br>*ATRX*<br>(rs3088074: C/G)
F7cl15	XIST+	C/G
ASD	XIST+	C/G
F002	XIST-	C/C
F7cl4	XIST-	C/G
CE	XIST-	C/G
CD	XIST-	C/G

E

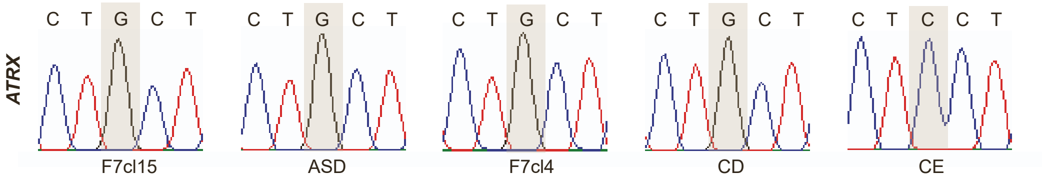

SUPPLEMENTARY FIGURE 2

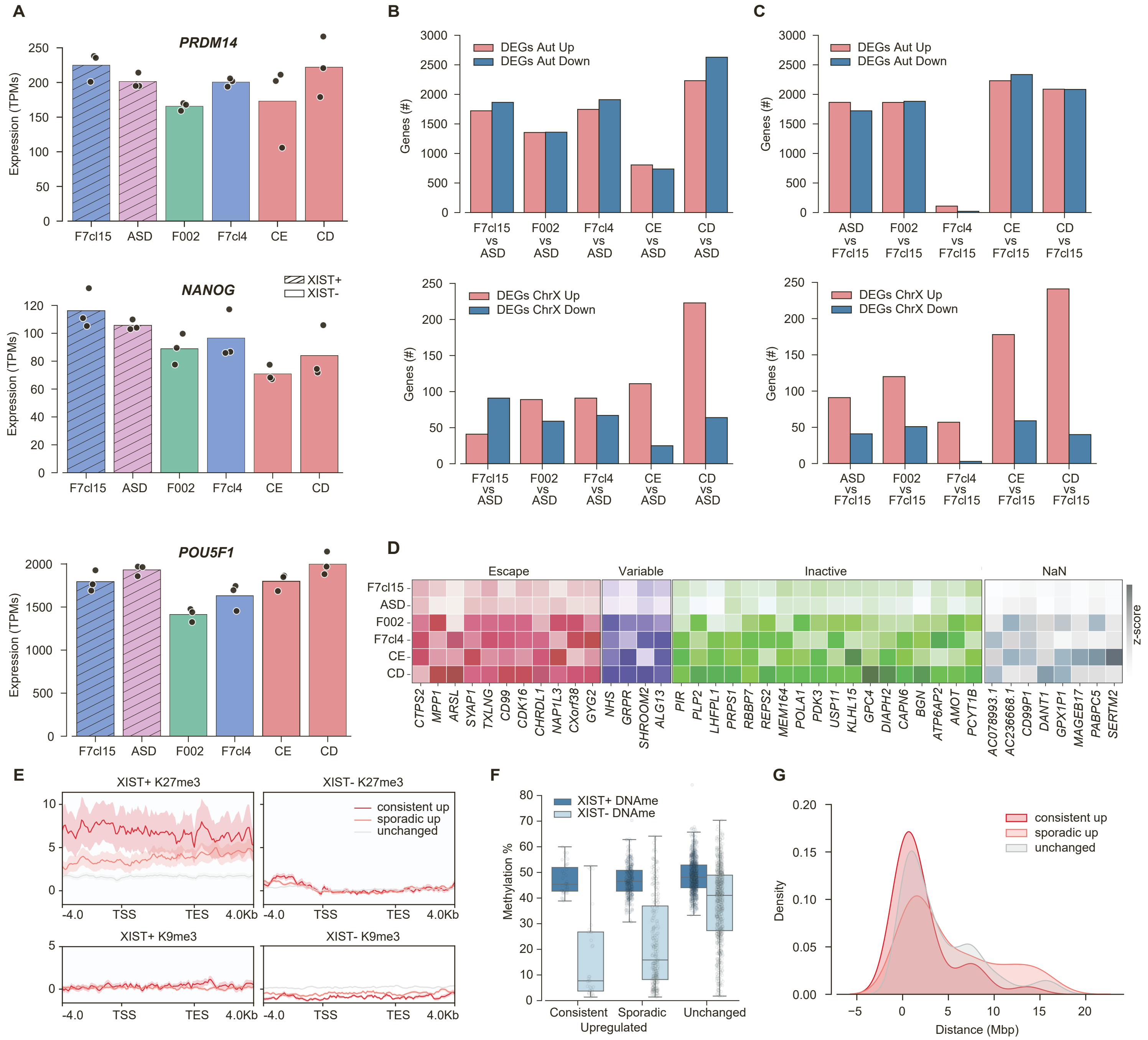

SUPPLEMENTARY FIGURE 3

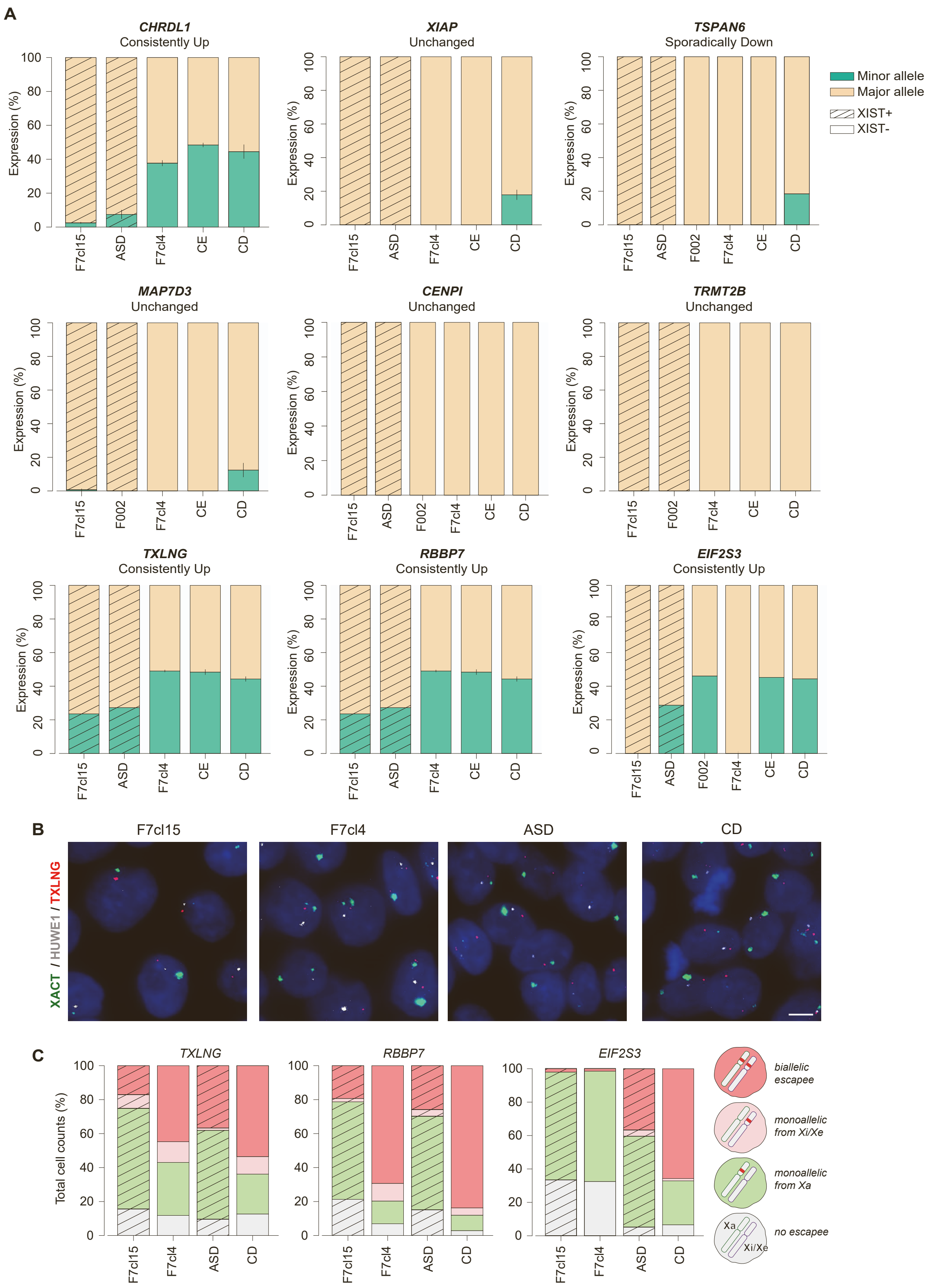

SUPPLEMENTARY FIGURE 4

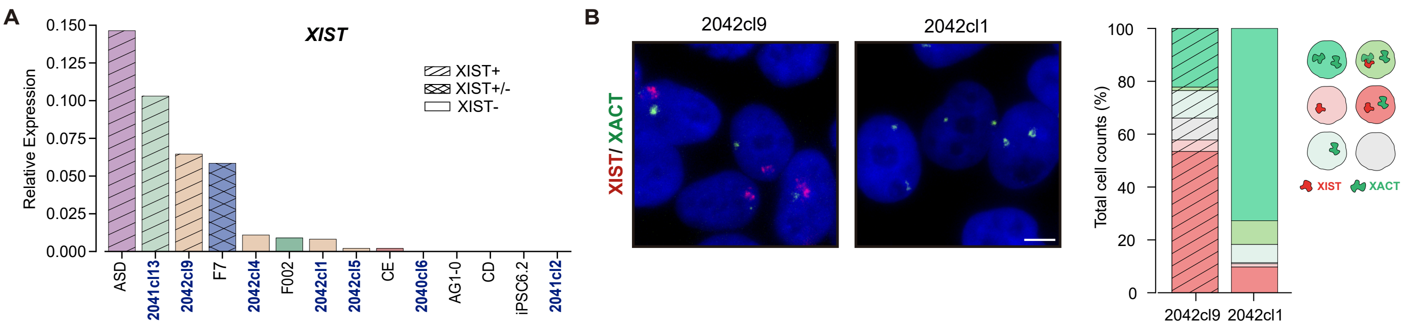

SUPPLEMENTARY FIGURE 5

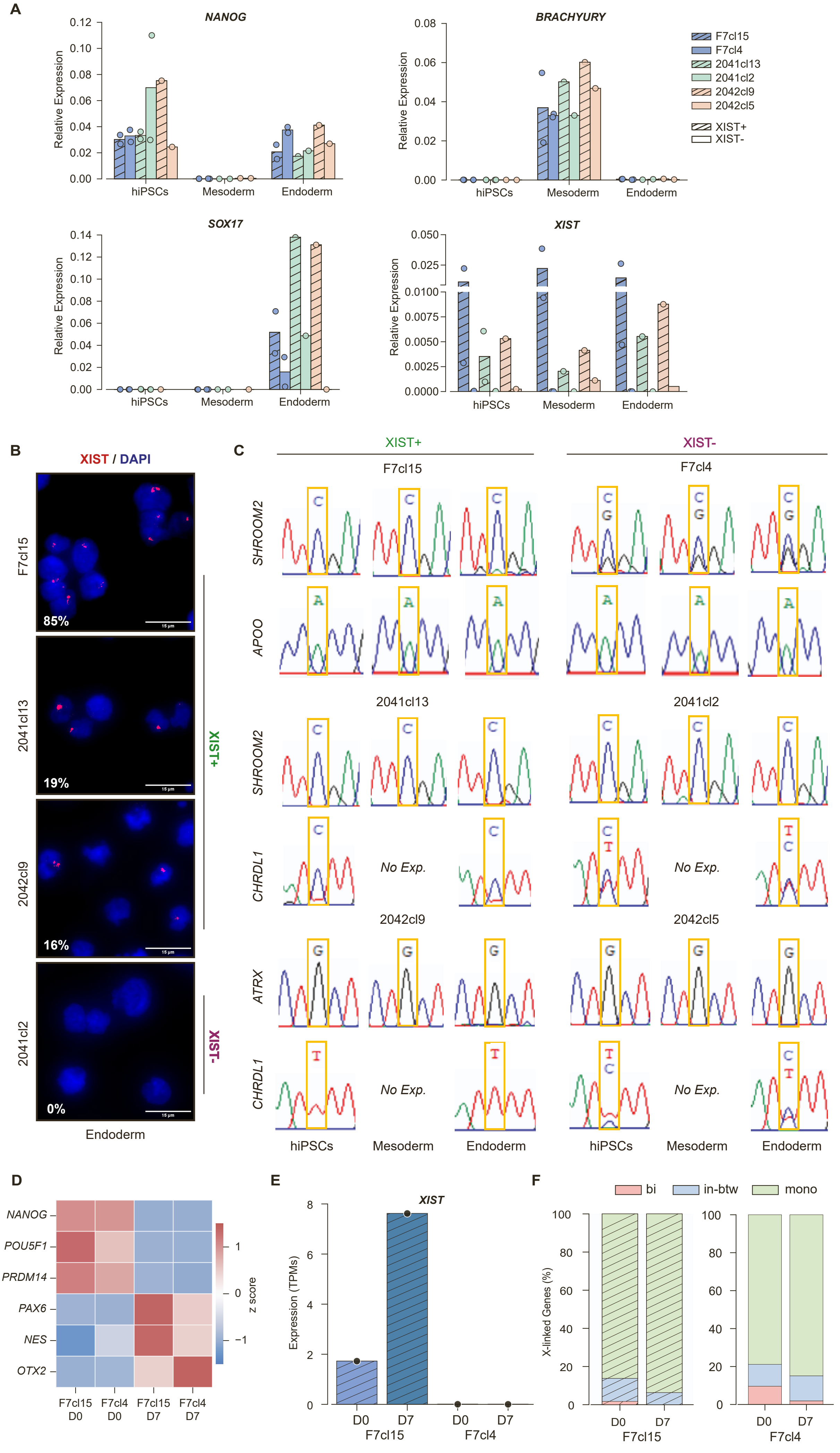

LIST OF SUPPLEMENTARY FIGURES

Fig. S1: Characterization of the XCI status in female hiPSCs

A. Schematic representation of YY1 binding sites within the *XIST* locus relative to A repeats motif. Bisulfite sequencing analysis within a region on *XIST* exon 1 containing YY1 binding sites in ASD, F002 and CD iPSCs. Each line represents the methylation profile of an independent PCR amplicon analyzed by Sanger sequencing and BiQ Analyser. White dots: unmethylated CpGs; black dots: methylated CpGs; % of methylated (Meth) CpGs was calculated as follows: average percentage of the number of methylated CpGs (black dots) / total number of CpGs (black + white dots) per cloned PCR product; the numbers in brackets represent the number of times the same amplicon was sequenced; the repetitive amplicons were only counted as one to determine the % of methylation. **B-C.** Representative RNA FISH images for *HUWE1* (red) and *ATRX* (green) in ASD, F002, CD and CE human hiPSCs (B) and for *POLA1* (red) and *HDAC8* (green) in isogenic F7cl15, ASD, F002, F7cl4, CD and CE iPSCs (C). DNA stained in blue by DAPI; scale bar: 5 µm; graphs represent % of cells with monoallelic (mono), biallelic (bi) or no expression (none). The values represent 1–2 independent experiments, where a minimum of 200 cells were counted per experiment. **D.** Summary table showing presence of heterozygosity at the SNP rs3088073 in the *ATRX* gene in F7cl15, ASD, F002, F7cl4, CE and CD human iPSCs. Bold letters refer to the presence of heterozygosity. **E.** Allelic expression of *ATRX* gene assayed by RT-PCR followed by Sanger sequencing. Chromatograms are shown for each cell line with the respective SNP highlighted in gray.

Fig. S2: Features of X-linked genes overexpressed in eroded hiPSCs

A. Expression analysis by RNAseq of *PRMD14*, *NANOG* and *POU5F1* pluripotent genes in F7cl15, ASD, F002, F7cl4, CE and CD hiPSCs. The graph shows the Transcripts per Million (TPMs) expression values from biological triplicates (black dots) of each sample. **B-C.** Barplots showing the number of upregulated (red) and downregulated (blue) Differentially Expressed Genes (DEGs) in autosomes (Aut) and X chromosome (ChrX) in F7cl15, F002, F7cl4, CE and CD hiPSCs compared with ASD XIST+ line (B) and with F7cl15 XIST+ line (C). **D.** Heatmap illustrating the expression levels of consistently upregulated genes categorized by their inactive/escape status (Escape, Inactive, Variable, and NaN) for multiple hiPSC lines (F7cl15, ASD, F002, F7cl4, CE, and CD). Each category has its genes color-coded to reflect expression levels, with darker shades representing higher expression levels. **E.** Metagene profiles illustrate the distribution of H3K27me3 and H3K9me3 signals in consistently upregulated, sporadically upregulated, and unchanged gene categories based on ChIP-seq data of XIST+ and XIST- hiPSCs from Yokobayashi et al., 2021 (see Materials and Methods for details). A two-sample t-test revealed significant differences for upregulated versus sporadically

upregulated genes and consistent upregulated versus unchanged genes (*p* << 0.01; Cohen's d > 1) for H3K27me3 in XIST+ cell lines. **F.** Boxplot showing the methylation percentage of promoter regions in consistently upregulated, sporadically upregulated, and unchanged genes in XIST+ and XIST- hiPSC lines from Bansal et al. 2021 (see Materials and Methods for details). Statistical comparisons were made using two-sample t-tests (*p* = 0.028 for consistently vs sporadic, *p* << 0.01 for sporadic vs unchanged) in cluster 3 (eroded state). **G.** Density plot showing the distribution of distances (in mega bases - Mbp) between each gene category (consistent up, sporadic up and unchanged) and their nearest upregulated constitutive escapee.

Fig. S3: RNAseq allele-specific expression analysis in female hiPSC lines

A. Allele-specific expression (ASE) based on RNAseq analysis from representative genes containing common SNPs across different hiPSCs. Bar plots displaying the average percentage of expression ± SEM of the major and minor alleles for the X-linked genes *SHROOM2*, *XIAP, TSPAN6*, *MEP7D3*, *CENPI*, *TRMT2B*, *TXLNG*, *RBPP7*, and *EIF2S3* in XIST+ (F7cl15, ASD) and XIST- (F002, F7cl4, CE and CD) hiPSCs. **B.** Representative RNA FISH images for *TXLNG* (red; escapee), XACT (green) and HUWE1 (white) in F7cl15 (XIST+), F7cl4 (XIST-), ASD (XIST+) and CD (XIST-) cell lines. DNA stained in blue by DAPI; scale bar: 5 µm; **C.** Quantification of single-cell expression levels for three escape genes (*TXLNG*, *RBBP7*, and *EIF2S3*) across these hiPSC lines. Bars indicate the proportion of cells expressing the escape gene from the active X chromosome (Xa, green), the inactive/eroded X chromosome (Xi/Xe, salmon), or both chromosomes (XaXi, red). Cells with no detectable expression are shown in gray. A minimum of 200 cells counted per experiment. Scale bar: 5 µm.

Fig S4: Analysis of XCI in the seven newly generated hiPSC lines during this study

A. Barplot showing RT-qPCR analysis of *XIST* expression normalized to *GAPDH* housekeeping gene in ASD, 2041cl13, 2042cl9, F7, 2042cl4, F002, 2042cl1, 2042cl5, CE, 2040cl6, AG1-0, CD, iPSC6.2 and 2041cl2 female hiPSCs. Bars represent the average *XIST/GAPDH* expression. n=1 for all iPSCs. **B.** Representative RNA FISH images for *XIST* (red) and *XACT* (green) in 2042cl9 and 2042cl1 hiPSCs; DNA stained in blue by DAPI; scale bar: 5 µm; graph represents % of cells with different expression profiles for XIST and XACT by RNA FISH as depicted in the legend on the left; a minimum of 200 cells were counted. Scale bar: 5 µm.

Fig S5: Erosion pattern persists upon mesodermal and endodermal commitment.

A. Barplots showing mean relative gene expression of *NANOG* (pluripotency marker), *BRACHYURY* (mesodermal marker), *SOX17* (endodermal marker) and *XIST* (Xi marker),

quantified by RT-qPCR upon normalization for *GAPDH* housekeeping gene in F7cl15, F7cl4, 2041cl13, 2041cl2, 2042cl9 and 2042cl5 hiPSCs, mesodermal and endodermal cells; n=2 for all samples, except for undifferentiated 2042 clones and differentiated (Endoderm and Mesoderm) 2041 and 2042 clones (n=1); Note that the barplots of *NANOG* and *XIST* for iPSCs are the same represented in Fig. 5B. **B.** Representative images of XIST RNA-FISH and respective percentages of cells expressing XIST (red dots) in F7cl15, 2041cl13, 2042cl9 and 2041cl2 at differentiated endodermal cells. The nuclei are counterstained with DAPI (blue). Scale bars represent 15 μm. Number of cells counted: F7cl15: 327, 2041cl13: 266, 2042cl9: 243, 2041cl2: 164. The values represent 1 independent experiment. **C.** Allelic expression assayed by RT-PCR followed by Sanger sequencing resourcing to informative SNPs to distinguish the two alleles. The chromatograms represent illustrative examples of the allelic expression of heterozygous X-linked genes for each XIST+/XIST- isogenic hiPSC pair in hiPSCs and after mesoderm and endoderm specification: *SHROOM2* and *APOO* gene for the F7cl15 and F7cl4, *SHROOM2* and *CHRDL1* gene for 2041cl13 and 2041cl2, and *ATRX* and *CHRDL1* gene for the 2042cl9 and 2042cl5. Note that chromatograms for iPSCs are the same as illustrated in Fig. 5D; **D.** Heatmap showing the RNAseq expression levels for pluripotent markers (*NANOG*, *POU5F1* and *PRDM14*) and ectoderm-specific markers (*PAX6*, *NES* and *OTX2* in F7cl15 (XIST+) and in F7cl4 (XIST-) cells before (D0) and after (D7) ectodermal differentiation. **E.** *XIST* expression data by RNAseq in F7cl15 and F7cl4 before (D0) and after (D7) ectodermal differentiation (D7) (n=1). The graph shows the Transcripts per Million (TPMs) expression value. **F.** Percentage of genes classified as biallelic, intermediate, or monoallelic in F7cl15 (XIST+) and F7cl4 (XIST-) cells before (D0) and after (D7) ectodermal differentiation. Classes were defined based on the minor allele frequency (Methods for details).

<u>LIST OF SUPPLEMENTARY TABLES</u>

Table S1: Description of original Cell Lines used in this study

Table S2: Differential Gene Expression Analysis (DGEA) of X-linked Genes in all cell lines

Table S3: Allelic Specific Expression (ASE) of X-linked Genes in all cell lines

Table S4: Description of Cell Lines generated by reprogramming fibroblasts

Table S5: Methylation analysis of 7 imprinted locus

Table S6: Informative SNPs found for the different isogenic pairs

Table S7: Allelic Specific Expression (ASE) of X-linked Genes in Cardiac Cells

Table S8: Primers and Conditions

EXPANDED MATERIAL AND METHODS

Ethics

hiPSC lines used in this study were either purchased or previously generated by us (Pólvora-Brandão et al., 2018; Silva et al., 2021b, 2021a) (Table S1; Table S4). Written informed consents were obtained by the donor or their legal guardian and ethically approved by the Ethics committee of the Lisbon Academic Medical Center, Lisbon, Portugal (approval numbers: 535/12 and 170/18).

Stem Cell Culture

All the hiPSC lines used in this study were cultured with mTeSR™ Plus medium (#05825, Stem Cell Technologies) in 6-well plates previously coated with Matrigel™ (#354230, Corning). Medium was changed every 24/48 hours (hrs) and the cells were routinely passed using 0.5 mM EDTA (#15575020, Invitrogen) in 1x Phosphate-Buffered Saline (PBS; #21600-044, Gibco). Cells were grown at 37ºC and kept in a humidified 5% CO_2 incubator in normoxia conditions.

To freeze, hiPSCs were dissociated with 0.5 mM EDTA/1x PBS and collected with Washing medium (Dulbecco's Modified Eagle Medium/Nutrient Mixture F-12 - DMEM-F12 #11320-033, Gibco, 10% KnockOut™ Serum Replacement - KSR, #10828-028, Gibco, 1% MEM Non-essential Amino Acid Solution 100x - NEAA, #11140-050, Gibco), 1 mM L-Glutamine (#25030081, Thermo Fisher Scientific), 0.1 mM β-mercaptoethanol, (#31350-010, Gibco). After 3 minutes at 1000 rotations per minute (rpm) of centrifugation, the cells pellet was resuspended with a freezing medium composed of 90% KSR and 10% of Dimethyl Sulfoxide (DMSO, #D2438, Merck). Cell vials were stored in a liquid nitrogen tank.

iPSC cultures were regularly scanned for mycoplasma contamination using the qPCR Mycoplasma Test (Mycoplasmacheck, Eurofins Genomics), following the manufacturer's instructions.

RT-qPCR

Total RNA was isolated from all hiPSCs lines using NZYol™ RNA Isolation Reagent (#MB18501, NZYTech) and then treated with DNaseI (#04716728001, Roche) to remove contaminating DNA and according to manufacturer's instructions. DNaseI-treated RNA (500 ng) was reverse-transcribed using random primers and a High-Capacity cDNA Reverse Transcription Kit (#4368814, Applied Biosystems) according to the manufacturer's instructions. Reverse Transcriptase quantitative PCR (RT-qPCR) was performed using NZYSpeedy qPCR Green Master Mix ROX (#MB22302, NZYTech) or NZYSpeedy qPCR Green Master Mix ROX Plus (#MB22202, NZYTech) in StepOne™ or ViiA™ 7 Real-Time PCR

Systems (Applied Biosystems). All PCR reactions were done with technical duplicates or triplicates and then normalized to the *GAPDH* housekeeping gene. The primers used are listed in Table S8. The results were analyzed with the StepOne™ or the QuantStudio™ RT-PCR softwares. The relative expression of each gene was determined using the $2^{-\Delta\Delta CT}$ method.

PCR/RT-PCR followed by Sanger Sequencing

To verify the presence of a specific SNP in ASD, F7, F002, CD, CE, 2041cl13, 2041cl2 and 2042cl9, 2042cl5 hiPSCs, genomic DNA isolated using conventional phenol:chloroform:isoamyl alcohol (#15593-031, Invitrogen) extraction was amplified using primers in the PCR section of Table S8. To analyze relative allelic expression of X-linked genes in F7cl15, F7cl4, 2041cl13, 2041cl2, 2042cl9, 2042cl5, ASD, F002, CD and CE hiPSCs and their ectodermal, mesodermal or endodermal derivatives, cDNA synthesized as described in RT-qPCR section was amplified by PCR using the primers in Table S8. Both PCR products (DNA or cDNA) were cleaned using the NZYGelpure kit (#MB01102, NzyTech) and sent for Sanger sequencing to STABVIDA with data visualized and analyzed using Chromas v2.6.2 software.

RNA FISH

The templates used for probe production were the following: XIST, a plasmid containing the 10Kb exon 5-6 plasmid (Rosspopoff et al., 2023); *XACT*: RP11-35D3 Bacterial Artificial Chromosome (BAC); *ATRX*: RP11-42M11 BAC; *HUWE1*: RP11-155O24 BAC; *HDAC8*: RP11-1021B19 BAC; *POLA1*: RP11-1104L9 BAC; *EIF2S3*: WI2-1347O20 fosmid; *TXLNG*: WI2-1095J6 fosmid; *RBBP7*: WI2-648C17 fosmid. Plasmid, fosmid or BAC probes were prepared using the Nick translation DNA labeling system 2.0 (#ENZ-GEN111-0050, Enzo) with red, green or Cy5 dUTPs (red: #ENZ-42844L-0050, green: #ENZ-42831L-0050, Enzo; Cy5: PA55022, Cytiva). RNA FISH was performed according to previously published protocol (Bousard et al., 2019). For probe preparation, 4 µl of probe was precipitated using 1/10 3M NaAc (#S2889, Sigma-Aldrich), sheared salmon sperm DNA (#AM9680, Invitrogen), human *COT1* DNA (#15279011, Invitrogen) and 3 volumes of ethanol (#10000652, Fisher Chemical). The pellet was resuspended in 6 µl of deionized formamide (#F9037, Sigma) and dissolved for 15 min at 37ºC with agitation. Then, the probes were denatured at 75ºC for 7 min and incubated at 37ºC for 30 min to prevent non-specific hybridization by *COT1* DNA. The probes were co-hybridized in hybridization buffer (20% dextran sulfate (#42867-5G, Sigma), 2x saline-sodium citrate (SSC; #S6639, Sigma-Aldrich), 1 µg/µl BSA (#R396A, Promega), 10 mM vanadyl-ribonucleoside (VRC; #S1402S, New England Biolabs) overnight at 37ºC. For the experiments on Fig.1B-C, Fig.S1B-C and Fig. S4B), hiPSCs were grown on matrigel-coated coverslips, while hiPSCs and ectodermal and endodermal differentiated cells in Fig. 5C and

Fig. S5B were dissociated with Accutase^{TM} (#A6964, Merck) and incubated onto poly-L-lysine (#P4832, Sigma)-coated 22x22 mm coverslips (#0101050, Marienfeld) for 5 min before RNA FISH procedure. Then, cells were fixed in 3% paraformaldehyde (PFA; #043368.9M, Thermo Fisher Scientific) for 10 min at room temperature (RT) and permeabilized for 5 min in 0.5% Triton X-100 (#T9284, Sigma) with 2 mM VRC diluted in PBS on ice. Cells were then dehydrated through 3 min incubations in 70%, 80%, 95% and 100% ethanol solutions and air-dried for 10 min before hybridization with probes. Coverslips were hybridized with fluorescent-labeled probes overnight at 37ºC in a humid chamber with FA/SSC solution (50% deionized formamide, 2x SSC). On the next day, washes were carried out using FA/SSC solution, three times for 7 min at 42ºC, and then with 2x SSC, three times for 5 min at 42ºC. Nuclei were stained with 1:10.000 dilution of DAPI 0.2 mg/mL (#D9542, Sigma-Aldrich) in 2x SSC for 3 min at RT. Coverslips were then mounted on slides with mounting media. Cells were observed with the widefield fluorescence microscope Zeiss Axio Observer (Carl Zeiss MicroImaging) using a 63x oil objective and filter sets FS43HE, FS38HE, FS49 and FS50. More than 200 cells were counted per experiment. For the RNA FISH analysis of escapees (*TLXNG, RBBP7 and EIS2F3*) in Fig. S3B, only cells positive for *HUWE1* and *XACT* RNA FISH signals were considered for counting.

Bisulfite sequencing

Genomic DNA was purified using conventional phenol:chloroform:isoamyl alcohol extraction. Bisulfite treatment was performed using the EZ DNA Methylation Gold Kit (#D5006, Zymo Research) following manufacturer's guidelines. Bisulfite-treated DNA was amplified by PCR for the YY1 binding sites within exon 1 of *XIST* (Fig. S1A) using the primers and conditions summarized in Table S8. PCR products were cloned into the pGEM-T Easy vector (#A1360, Promega) and at least 10 clones from each sample were sequenced. Methylation analysis was performed using BiQ Analyser v2.02 (Bock et al., 2005).

Whole Exome-sequencing (WES)

Genomic DNA from ASD, F7, F002 and CD were purified using conventional phenol:chloroform:isoamyl alcohol extraction. Genomic DNA (1.5µg) was sent to NOVOGENE that conducted whole-exome sequencing (WES). Briefly, genomic DNA was fragmented into 180–280 bp by sonication and subjected to library preparation using the Agilent SureSelect Human All Exon V6 Kit (#5190-8864, Agilent Technologies). The enriched libraries underwent paired-end 150bp sequencing on the Illumina HiSeq 2000 platform.

Raw WES data from hiPCS cell lines was preprocessed with TrimGalore v0.4.4 (Martin, 2011) to remove possible sequencing adapters and filter sequences by Phred quality scores ("-q 20 –length 75 --stringency 5 --trim-n --max_n 2). Reads were further aligned with bwa mem

(v0.7.15-r1140) (Li, 2013) against the GRCh38 genome assembly and duplicates were flagged with GATK4 MarkDuplicatesSpark (McKenna et al., 2010). Base scores were recalibrated (GATK4 Base Quality Score Recalibration), and a germline joint calling approach was performed with GATK4 HaplotypeCaller (Poplin et al., 2018) and GenotypeGVCFs to generate raw genotype calls. Variant calling was restricted to regions covered by Agilent v6 kit. Raw variant filtering was done with GATK4 VariantFiltration by employing hard filters based on several annotations (default values following GATK recommendations). All GATK4-based analyses were executed using the GATK version 4.1.2.0. Further processing of the call set was performed with bcftools v1.9 (Li, 2011) (multiallelic sites split, indel normalization and quality filtering), where thresholds of GQ > 30, DP > 20 reads and a minimum of 7 reads of the least covered allele were required (MIN(FMT/AD > 7). Filtered variants were annotated with Ensembl VEP (McLaren et al., 2016) using the 96 release. Chromosome X variants were further selected for downstream analysis.

RNA-sequencing (RNA-seq) library preparation and analysis

Triplicates of F7cl15, ASD, F002, F7cl4, CE and CD hiPSC lines as well as one replicate of an ectodermal differentiation series for the F7cl15 and F7cl4 hiPSC pair (F7cl4 D0 and D7 & F7cl15 D0 and D7) were used for RNA-seq. Total RNA was isolated using NYZol and then DNase I-treatment was performed to remove contaminating DNA following the manufacturer's recommendations. RNA (1 µg) was sent to NOVOGENE where quality of the samples was verified on a 2100 Agilent Bioanalyser system. Only samples with RIN score above 9 were processed. RNA was used for 250–300 bp insert cDNA library following manufacturer's recommendations and libraries were sequenced with NovaSeq 6000 platform using paired-end 150-bp mode.

To quantify gene expression from our bulk RNAseq data (paired-ended strand-specific), we mapped the reads to the reference genome (GRCh38 assembly; release 37, GRCh38.p13) using STAR (v2.7.8a) (Dobin et al., 2013) using the *--quantMode GeneCounts* option.

The number of raw reads mapping to the X chromosome (relative to the total number of reads) was used as a proxy for erosion (Fig 2B). We calculated Transcripts per Million (TPMs) for all genes in all samples directly from the read count matrix using a custom python script.

$$TPM = A \frac{1}{\sum A} \times 10^6$$

$$\text{where } A = \frac{Total\ read\ mapped\ to\ gene \times 10^3}{gene\ length\ in\ bp}$$

This TPM matrix was then used to show *XIST* expression levels across samples and perform hierarchical clustering.

Differential gene expression was assessed using the DESeq2 (v 1.40.2) R package (Love et al., 2014). We compared each XIST- hiPSC against each XIST+ hiPSC line and filtered differentially expressed genes (DEGs) by establishing a threshold of |log2FC| = 0.33 and an adjusted p-value < 0.05. This threshold was chosen to encompass most genes that reactivate from the Xi regardless of whether they are inactive, variably expressed, or inactive genes, with an expected increase in bulk expression ranging from 1.25 to 2 times. We divided X-linked genes into five different categories according to their behavior in each comparison: consistently upregulated/downregulated (if the gene was upregulated/downregulated in three or four cell lines when compared against both ASD and F7cl15 lines); sporadically upregulated/downregulated (if the gene was upregulated/downregulated only in one or two cell lines when compared against both XIST+ controls) or unchanged (if they did not meet any of the previous criteria: e.g. gene was considered upregulated against F7cl15, but not against ASD; gene was upregulated against ASD, but downregulated against F7cl15).

Furthermore, we categorized X-linked genes according to their XCI status as inactive, variable and escapees following the classification by Tukiainen et al 2017 and then reviewed by Werner et al., 2022 (Tukiainen et al., 2017; Werner et al., 2022). We consider the classes reviewed by Werner *et al.* as reference and the classification from Tukiainen *et al*. 2017 for the remaining genes not classified by Werner *et al*., 2022.

Allele-Specific Expression (ASE) Analysis

After conducting WES and storing gene sequence variations in a Variant Call Format (VCF), we used phASER (v.0.9.9.4) (Castel et al., 2016) for RNAseq-based phasing, enabling the generation of gene-level haplotype expression data. Only reads uniquely mapped and with a base quality ≥10 were used for phasing. For our downstream analyses, we further discarded loci with a total read depth lower than 10. This limits the number of genes in our analysis but reduces the number of false positives due to biased RNA-seq read mapping or other technical artifacts. The effect size of allelic imbalance in expression for each gene in each sample was determined using the Minor Allele Frequency (MAF), calculated as the ratio of minor allele read counts (the least common allele) to the total read counts from both alleles. We defined genes with a MAF < 0.10 as fully monoallelic, genes with a MAF > 0.40 as fully biallelic, and the remaining genes as “intermediate”.

Re-analyzing Epigenomic datasets: ChIP-seq and DNA methylation arrays

We integrated data from two studies to visualize the positioning of H3K27me3, H3K9me3, and DNA methylation levels across the X chromosome, comparing eroded and non-eroded conditions. From Yokobayashi et al. (Yokobayashi et al., 2021) (GEO: GSE165869), we used ChIP-seq peaks (bigwig files) from two female samples (F1 and F3) showing the distribution

of H3K27me3 and H3K9me3 marks. F3 (XIST+, non-eroded) shows H3K27me3 and H3K9me3 distributed along the X chromosomes in a largely mutually exclusive manner, while F1 (XIST-, eroded) shows loss of H3K27me3 and enrichment of H3K9me3, but to confined/narrow regions.

To corroborate the enrichment of each histone mark in specific gene categories ('consistently' and 'sporadically' upregulated and unchanged), we generated metagene profiles to visualize the distribution of histone mark signals across key genomic regions. Using the *computeMatrix* tool from *deepTools* ((Ramírez et al., 2014); v3.5.1), we calculated normalized scores across genomic regions, enabling unbiased comparisons across genes. The scale-regions mode was used to standardize regions of interest by stretching or shrinking them to a uniform length. We set a bin size of 1 kbp and a body length of 5 kbp, ensuring consistent visualization of scores across regions of varying lengths. The resulting profiles were visualized using *deepTools plotProfile*.

DNA methylation data from Bansal et al. (2021) obtained from Illumina 450K and 850K Methylation Array data for primed human iPSC and ESC samples was used to examine variations in methylation levels throughout the progression of XCI erosion (Bansal et al., 2021). Probes with higher variance in female samples compared to male samples ($p \leq 0.01$) were selected for K-means clustering of female samples. This approach identified six distinct clusters, ordered by their average methylation levels, and revealed all differentially methylated probes (DMPs) between neighboring clusters. The analysis demonstrated a clear, systematic pattern of stepwise de-methylation changes, highlighting the progressive erosion of the Xi. We designated cluster A as the non-eroded ($XIST^{+}$) state and cluster C as the eroded ($XIST^{-}$) state, as the cluster C contained the biggest number of eroded hiPSCs. Clusters D, E and F, while more extensively eroded, cover a smaller number of hiPSCs and likely represent rarer states of erosion not phenocopied by our hiPSC lines.

To quantify the correlation between the location of upregulated genes and regions with highly variable changes in methylation levels, we compared the percentage of methylation in regions where upregulated genes were located across different categories (consistent up, sporadic up and unchanged). Specifically, we focused on methylation levels in the promoter regions of these genes. Statistical comparisons between groups within each cluster (cluster A and cluster C) were conducted using two-sample t-tests. The resulting p-values were assessed for statistical significance using a threshold of $p < 0.05$. Consistently upregulated genes are significantly different from sporadically upregulated genes ($p = 0.028$), and sporadically upregulated genes are significantly different from unchanged genes ($p < 0.001$) in cluster three (eroded state).

Distances to escapee genes

A comprehensive list of all genes was compiled, along with a second list containing only constitutively upregulated escapees. Using *bedtools closest* (Quinlan and Hall, 2010); v.2.30.0), the closest escapee - whether upstream or downstream and regardless of strand orientation - was identified for each gene in the first list, and the distance between them was recorded. The average distances were then calculated for consistently upregulated, sporadically upregulated, unchanged, and downregulated genes, and the results were visualized in a plot. Statistical analysis using the Mann-Whitney test revealed that consistently upregulated genes were significantly different from all other categories ($p<0.01$), while sporadically upregulated and unchanged genes were not significantly different from each other ($p=0.12$).

5mC/5hmC measurements by Liquid Chromatography-Mass Spectrometry (LC-MS)

Genomic DNA from hiPSCs was purified using conventional phenol:chloroform:isoamyl alcohol extraction and digested using DNA Degradase Plus (#E2020, Zymo Research) according to the manufacturer's instructions. Nucleosides were analyzed by LC-MS/MS on a Q-Exactive mass spectrometer (Thermo Scientific) fitted with a nanoelectrospray ion-source (Proxeon). All samples and standards had a heavy isotope-labeled nucleoside mix added prior to mass spectral analysis (2′-deoxycytidine-$^{13}C_1$, $^{15}N_2$ (#SC-214045, Santa Cruz), 5-(methyl-$^{2}H_3$)-2′-deoxycytidine (#SC-217100, Santa Cruz), 5-(hydroxymethyl)-2′-deoxycytidine-$^{2}H_3$ (#H946632, Toronto Research Chemicals). MS2 data for 5hmC, 5mC and C were acquired with both the endogenous and corresponding heavy-labeled nucleoside parent ions simultaneously selected for fragmentation using a 5 Th isolation window with a 1.5 Th offset. Parent ions were fragmented by Higher-energy Collisional Dissociation (HCD) with a relative collision energy of 10%, and a resolution setting of 70,000 for MS2 spectra. Peak areas from extracted ion chromatograms of the relevant fragment ions, relative to their corresponding heavy isotope-labeled internal standards, were quantified against a six-point serial 2-fold dilution calibration curve, with triplicate runs for all samples and standards.

IMPLICON Library Preparation and Analysis

IMPLICON was performed as previously described (Klobučar et al., 2020) in F7cl15, F7cl4, CD, CE, 2040cl6, 2042cl1 and 2042cl9 hiPSCs, and, 2040 and 2042 fibroblasts (2040 Fib, 2042 Fib). Briefly, following bisulfite conversion, a first PCR amplifies each region per sample in individual reactions, adding adapter sequences, as well as 8 random nucleotides (N8) for subsequent data deduplication. PCR conditions and primers for this first step are listed in Table S8. After pooling amplicons for each biological sample and clean-up using AMPure XP magnetic beads (#A63880, Beckman Coulter), a second PCR completes a sequence-ready

library with sample-barcodes for multiplexing. In this PCR reaction, barcoded Illumina adapters are attached to the pooled PCR samples ensuring that each sample pool receives a unique reverse barcoded adapter. Libraries were verified by running 1:30 dilutions on an Agilent bioanalyzer and then sequenced using the Illumina MiSeq platform to generate paired-end 250 bp reads using the indexing primer with the following sequence, 5'-AAGAGCGGTTCAGCAGGAATGCCGAGACCGATCTC-3' and 10% PhIX spike-in as the libraries are of low complexity.

IMPLICON bioinformatics analysis was also performed as described (Klobučar et al., 2020), following the step-by-step guide of data processing analysis. Briefly, data was processed using standard Illumina base-calling pipelines. As the first step in the processing, the first 8 bp of Read 2 were removed and written into the readID of both reads as an in-line barcode, or Unique Molecular Identifier (UMI). This UMI was then later used during the deduplication step with "deduplicate bismark–barcode mapped_file.bam". Raw sequence reads were then trimmed to remove both poor quality calls and adapters using Trim Galore v0.5.0 (Martin, 2011). Trimmed reads were aligned to the human reference genome in paired-end mode. Alignments were carried out with Bismark v0.20.0 and deduplication was then carried out with *deduplicate_bismark*, using the–barcode option to take UMIs into accountCpG methylation calls were extracted from the mapping output using the Bismark methylation extractor. Coverage files were imported into Seqmonk software v1.47 (Babraham Bioinformatics; RRID: SCR_001913) for all downstream analysis. Probes were made for each CpG contained within the amplicon and quantified using the DNA methylation pipeline or total read count options.

Trilineage Specification

Trilineage differentiation of F7cl15, F7cl4, 2041cl13, 2041cl2, 2042cl9 and 2042cl5 hiPSCs was performed using STEMdiff^{TM} Trilineage Differentiation Kit (#05230, Stem Cell Technologies) to differentiate into ectodermal, mesodermal and endodermal lineages according to manufacturer's instructions. For ASD, F002, CE and CE hiPSCs, ectodermal differentiation was also performed. These experiments were conducted with at least one or two replicates (Fig. 5; Fig. S5). Briefly, a density of 200,000 (mesoderm lineage) or 800,000 (endoderm and/or ectoderm lineages) of cells were plated in 12-well plates on day 0. The medium was changed daily until day 5 (mesoderm and endoderm lineages) or day 7 (ectoderm lineage). After trilineage commitment, the cells were collected with NZYol reagent to perform RNA extraction followed by RT-qPCR or RNA-seq (for F7cl15/F7cl4 ectoderm differentiation) or dissociated with accutase to perform RNA FISH and RT-qPCR experiments. The primers used are listed in Table S8.

Cardiac Differentiation

The cardiac differentiation of ASD, F002, CD and CE hiPSCs was performed following a published protocol (Lian et al., 2012). Briefly, the hiPSCs were initially cultured in matrigel-coated plates in mTeSR1 medium (# 85850, STEMCELL Technologies) until full confluency. The differentiation was initiated by removing mTeSR1 medium and adding RPMI/B-27 without insulin (#A1895601, Thermo Fisher Scientific) and containing 6 µM of CHIR99021 (#04-0004, Stemgent), a GSK3 inhibitor. At day 3 (D3), to induce the cardiac fate of the mesendoderm progenitor cells, inhibition of canonical Wnt signaling is performed using IWP-4 (#72552, STEMCELL Technologies) a Wnt signaling inhibitor. Cardiac cells spontaneously develop into contracting cardiomyocytes when cultured in RPMI/B-27 medium and are left until day 30 of differentiation. At day 30 of differentiation (D30), the cardiomyocytes were collected with NZYol reagent to perform RNA extraction followed by RT-qPCR and RNA-AMP-seq using the primers listed in Table S8.

RNA AMPLICON-sequencing (RNA-AMP-seq) Library Preparation and Analysis

Total RNA was isolated, DNaseI treated and reverse-transcribed as described in the RT-qPCR section of the Methods. RNA-AMP-seq was performed for the samples before (ASD, F002, CD and CE hiPSCs) and after cardiac differentiation (ASD, F002, CD, CE hiPSC-CM) using a similar procedure previously employed for IMPLICON. Briefly, a first PCR amplifies each region per sample in individual reactions, adding adapter sequences, as well as 8 random nucleotides (N8) for subsequent data deduplication. PCR conditions and primers for this first step are listed in Table S8. After pooling amplicons for each biological sample and clean-up using AMPure XP magnetic beads, a second PCR completes a sequence-ready library with sample-barcodes for multiplexing. In this PCR reaction, barcoded Illumina adapters are attached to the pooled PCR samples ensuring that each sample pool receives a unique reverse barcoded adapter. Libraries were verified by running 1:30 dilutions on an Agilent bioanalyzer and then sequenced using the Illumina MiSeq platform to generate paired-end 250 bp reads using the indexing primer with the following sequence, 5'-AAGAGCGGTTCAGCAGGAATGCCGAGACCGATCTC-3’ and 10% PhIX spike-in as the libraries are of low complexity.

RNA-AMP-seq data was first processed using standard Illumina base-calling pipelines. Briefly, the first 8 bp of Read 2 were removed and written into the readID of both reads as an in-line barcode, or Unique Molecular Identifier (UMI). This UMI was then later used during the deduplication step with “umi_tools dedup --umi-separator=':' -I "mapped_file.bam" --paired”. Raw sequence reads were then trimmed to remove both poor quality calls and adapters using Trim Galore v0.5.0 (doi: 10.5281/zenodo.5127899, Cutadapt version 1.15, parameters:–

paired). Trimmed reads were aligned to the human reference genome in paired-end mode. Alignments were carried out with STAR v2.7.11a. Deduplication was then carried out with UMI-tools v1.1 (see above). Aligned read (.bam) files were analyzed with phASER v.0.9.9.4 (Castel et al., 2016) for allelic expression quantification using the "phaser" and "phaser_gene_ae" commands.Minor Allelic Frequency (MAF) was then calculated as described above for the ASE analysis.

Statistics and Reproducibility

The statistical methods employed in each analysis are described in their respective sections. All these statistical tests were conducted using dedicated Python packages tailored to each specific analysis.

REFERENCES

- Bansal, P., Ahern, D.T., Kondaveeti, Y., Qiu, C.W., Pinter, S.F., 2021. Contiguous erosion of the inactive X in human pluripotency concludes with global DNA hypomethylation. Cell Rep. 35, 109215. https://doi.org/10.1016/j.celrep.2021.109215
- Bock, C., Reither, S., Mikeska, T., Paulsen, M., Walter, J., Lengauer, T., 2005. BiQ Analyzer: visualization and quality control for DNA methylation data from bisulfite sequencing. Bioinformatics 21, 4067–4068. https://doi.org/10.1093/bioinformatics/bti652
- Castel, S.E., Mohammadi, P., Chung, W.K., Shen, Y., Lappalainen, T., 2016. Rare variant phasing and haplotypic expression from RNA sequencing with phASER. Nat. Commun. 7, 12817. https://doi.org/10.1038/ncomms12817
- Dobin, A., Davis, C.A., Schlesinger, F., Drenkow, J., Zaleski, C., Jha, S., Batut, P., Chaisson, M., Gingeras, T.R., 2013. STAR: Ultrafast universal RNA-seq aligner. Bioinformatics 29, 15–21. https://doi.org/10.1093/bioinformatics/bts635
- Klobučar, T., Kreibich, E., Krueger, F., Arez, M., Pólvora-Brandão, D., von Meyenn, F., da Rocha, S.T., Eckersley-Maslin, M., 2020. IMPLICON: an ultra-deep sequencing method to uncover DNA methylation at imprinted regions. Nucleic Acids Res. 48, e92–e92. https://doi.org/10.1093/nar/gkaa567
- Lian, X., Hsiao, C., Wilson, G., Zhu, K., Hazeltine, L.B., Azarin, S.M., Raval, K.K., Zhang, J., Kamp, T.J., Palecek, S.P., 2012. Robust cardiomyocyte differentiation from human pluripotent stem cells via temporal modulation of canonical Wnt signaling. Proc. Natl. Acad. Sci. U. S. A. 109, E1848-1857. https://doi.org/10.1073/pnas.1200250109
- Love, M.I., Huber, W., Anders, S., 2014. Moderated estimation of fold change and dispersion for RNA-seq data with DESeq2. Genome Biol. 15, 550. https://doi.org/10.1186/s13059-014-0550-8
- McKenna, A., Hanna, M., Banks, E., Sivachenko, A., Cibulskis, K., Kernytsky, A., Garimella, K., Altshuler, D., Gabriel, S., Daly, M., DePristo, M.A., 2010. The Genome Analysis Toolkit: a MapReduce framework for analyzing next-generation DNA sequencing data. Genome Res. 20, 1297–1303. https://doi.org/10.1101/gr.107524.110
- Pólvora-Brandão, D., Joaquim, M., Godinho, I., Aprile, D., Álvaro, A.R., Onofre, I., Raposo, A.C., Pereira de Almeida, L., Duarte, S.T., da Rocha, S.T., 2018. Loss of hierarchical

imprinting regulation at the Prader–Willi/Angelman syndrome locus in human iPSCs. Hum. Mol. Genet. 27, 3999–4011. https://doi.org/10.1093/hmg/ddy274

- Poplin, R., Ruano-Rubio, V., DePristo, M.A., Fennell, T.J., Carneiro, M.O., Auwera, G.A.V. der, Kling, D.E., Gauthier, L.D., Levy-Moonshine, A., Roazen, D., Shakir, K., Thibault, J., Chandran, S., Whelan, C., Lek, M., Gabriel, S., Daly, M.J., Neale, B., MacArthur, D.G., Banks, E., 2018. Scaling accurate genetic variant discovery to tens of thousands of samples. https://doi.org/10.1101/201178
- Ramírez, F., Dündar, F., Diehl, S., Grüning, B.A., Manke, T., 2014. deepTools: a flexible platform for exploring deep-sequencing data. Nucleic Acids Res. 42, W187–W191. https://doi.org/10.1093/nar/gku365
- Rosspopoff, O., Cazottes, E., Huret, C., Loda, A., Collier, A.J., Casanova, M., Rugg-Gunn, P.J., Heard, E., Ouimette, J.-F., Rougeulle, C., 2023. Species-specific regulation of XIST by the JPX/FTX orthologs. Nucleic Acids Res. 51, 2177–2194. https://doi.org/10.1093/nar/gkad029
- Silva, T.P., Pereira, C.A., Oliveira, A.R., Raposo, A.C., Arez, M., Cabral, J.M.S., Milagre, I., Carmo-Fonseca, M., da Rocha, S.T., 2021a. Generation and characterization of induced pluripotent stem cells from a family carrying the BRCA1 mutation c.3612delA. Stem Cell Res. 52, 102242. https://doi.org/10.1016/j.scr.2021.102242
- Silva, T.P., Pereira, C.A., Raposo, A.C., Oliveira, A.R., Arez, M., Cabral, J.M.S., Milagre, I., Carmo-Fonseca, M., Rocha, S.T. da, 2021b. Generation and characterization of induced pluripotent stem cells heterozygous for the Portuguese BRCA2 founder mutation. Stem Cell Res. 53, 102364. https://doi.org/10.1016/j.scr.2021.102364
- Tukiainen, T., Villani, A.-C., Yen, A., Rivas, M.A., Marshall, J.L., Satija, R., Aguirre, M., Gauthier, L., Fleharty, M., Kirby, A., Cummings, B.B., Castel, S.E., Karczewski, K.J., Aguet, F., Byrnes, A., (…), Lappalainen, T., Regev, A., Ardlie, K.G., Hacohen, N., MacArthur, D.G., 2017. Landscape of X chromosome inactivation across human tissues. Nature 550, 244–248. https://doi.org/10.1038/nature24265
- Werner, J.M., Ballouz, S., Hover, J., Gillis, J., 2022. Variability of cross-tissue X-chromosome inactivation characterizes timing of human embryonic lineage specification events. Dev. Cell 57, 1995-2008.e5. https://doi.org/10.1016/j.devcel.2022.07.007
- Yokobayashi, S., Yabuta, Y., Nakagawa, M., Okita, K., Hu, B., Murase, Y., Nakamura, T., Bourque, G., Majewski, J., Yamamoto, T., Saitou, M., 2021. Inherent genomic properties underlie the epigenomic heterogeneity of human induced pluripotent stem cells. Cell Rep. 37, 109909. https://doi.org/10.1016/j.celrep.2021.109909
